# Co-expression modules of NF1, PTEN and sprouty enable distinction of adult diffuse gliomas according to pathway activities of receptor tyrosine kinases

**DOI:** 10.18632/oncotarget.10359

**Published:** 2016-07-01

**Authors:** Wanyu Zhang, Yuhong Lv, Yang Xue, Chenxing Wu, Kun Yao, Chuanbao Zhang, Qiang Jin, Rong Huang, Jiuyi Li, Yingyu Sun, Xiaodong Su, Tao Jiang, Xiaolong Fan

**Affiliations:** ^1^ Laboratory of Neuroscience and Brain Development, Beijing Key Laboratory of Gene Resources and Molecular Development, Beijing Normal University, Beijing, China; ^2^ Department of Neurosurgery, Beijing Sanbo Brain Hospital, Capital Medical University, Beijing, China; ^3^ Department of Pathology, Beijing Sanbo Brain Hospital, Capital Medical University, Beijing, China; ^4^ Beijing Neurosurgical Institute, Capital Medical University, Beijing, China; ^5^ Department of Neurosurgery, Beijing Tiantan Hospital, Capital Medical University, Beijing, China; ^6^ Biodynamic Optical Imaging Center (BIOPIC), School of Life Sciences, Peking University, Beijing, China; ^7^ Current address: Department of Cell Biology, Hebei Medical University, Hebei, Shijiazhuang, China

**Keywords:** glioma, molecular classification, receptor tyrosine kinase

## Abstract

Inter-individual variability causing elevated signaling of receptor tyrosine kinases (RTK) may have hampered the efficacy of targeted therapies. We developed a molecular signature for clustering adult diffuse gliomas based on the extent of RTK pathway activities. Glioma gene modules co-expressed with NF1 (NF1-M), Sprouty (SPRY-M) and PTEN (PTEN-M) were identified, their signatures enabled robust clustering of adult diffuse gliomas of WHO grades II-IV from five independent data sets into two subtypes with distinct activities of RAS-RAF-MEK-MAPK cascade and PI3K-AKT pathway (named RMPA^high^ and RMPA^low^ subtypes) in a morphology-independent manner. The RMPA^high^ gliomas were associated with poor prognosis compared to the RMPA^low^ gliomas. The RMPA^high^ and RMPA^low^ glioma subtypes harbored unique sets of genomic alterations in the RTK signaling-related genes. The RMPA^high^ gliomas were enriched in immature vessel cells and tumor associated macrophages, and both cell types expressed high levels of pro-angiogenic RTKs including MET, VEGFR1, KDR, EPHB4 and NRP1. In gliomas with major genomic lesions unrelated to RTK pathway, high RMPA signature was associated with short survival. Thus, the RMPA signatures capture RTK activities in both glioma cells and glioma microenvironment, and RTK signaling in the glioma microenvironment contributes to glioma progression.

## INTRODUCTION

Gliomas are the most common primary tumors in the adult central nervous system [1]. Despite considerable efforts to search for the etiology and to explore targeted therapies, the majority of the grade IV glioma (glioblastoma, GBM) patients die within 1 to 2 years of diagnosis [2]. Low-grade gliomas will eventually progress to the GBM stage and this is followed by a rapid fatal outcome for patients. New therapeutic approaches are therefore needed for combating gliomas. Elevated signaling activities of receptor tyrosine kinases (RTK) constitutes one of the known core signaling pathways identified in GBMs and in a fraction of low-grade gliomas [3–5]. Animal models of glioma show that RTK-related signaling may play a crucial role in the pathogenesis of the disease [6, 7]. A large number of studies have investigated the mechanism of enhanced RTK activities in gliomas and explored the option of using the RTK signaling pathways as therapeutic targets [8, 9]. However, the resulting therapeutic benefits to glioma patients have been minimal [10].

Several mechanisms can contribute to elevated RTK signaling activities in gliomas [3, 4, 8]. Somatically occurring genomic alterations in RTK or RTK signaling-related molecules can cause elevated RTK signaling [8]. *EGFR*, *PDGFRA*, *FGFR1*, *FGFR2*, *FGFR4* and *MET* are frequently amplified, mutated, or fused in high-grade gliomas or in secondary GBMs [3, 4, 11–14]. Co-activation of multiple RTKs, and mosaic amplification of *EGFR* and *PDGFRA* have been observed in a small subset of GBMs [15–17].

RTK signaling is mediated by the RAS-RAF-MEK-MAPK cascade and the phosphatidylinositol 3-kinase (PI3K)-AKT pathway, which in turn are controlled by negative feedback loops. Along the RAS-RAF-MEK-MAPK cascade, somatic mutations which inactivate or delete *NF1*, the RAS-GTPase inhibitor, are found in about 10% of the GBMs. A non overlapping minor subset of GBMs harbor the *BRAF* V600E mutation [3, 4]. Both of these alterations can result in activation of the RAS-RAF-MEK-MAPK cascade. In the PI3K-AKT pathway, mutations, including deletions, in *PTEN*, and amplification or activating mutations in the catalytic component p110α or regulatory component p85α of PI3K, occur in GBMs in a mutually exclusively manner. These result in the activation of the PI3K-AKT pathway in 90% of the GBMs [4]. Downstream of the RAS-RAF-MEK-MAPK cascade and PI3K-AKT pathway, sprouty (SPRY) proteins represent a major class of ligand-inducible inhibitors of RTK-dependent signaling pathways. The SPRY proteins are key negative regulators that limit the strength, duration and range of activation of RTKs, counteracting both RAS-RAF-MEK-MAPK and PI3K-AKT signaling pathways [18].

The interplay between the various cell types in the glioma microenvironment may also contribute to elevated RTK signaling [19, 20]. GBMs with poor prognosis contain high numbers of tumor-associated macrophages (TAM), recruited to the glioma microenvironment by CSF1 expressed by the glioma cells [21]. High level expression of PDGF and EGF in TAMs supports tumour cell growth and triggers angiogenesis in the glioma environment [22]. EGFR signaling in glioma cells regulates the expression of the angiogenic factor VEGF [23, 24]. However, glioma microenvironment-derived RTK signature has not so far been clearly defined.

Elevated RTK signaling in individual gliomas may therefore be brought about by a variety of mechanisms. The inter-individual variability in the mechanisms of RTK activation, and the heterogeneity within morphologically diagnosed glioma subgroups may have compromised the understanding of glioma pathobiology, and thus the design and assessment of the therapies targeting RTK-related signaling [10]. The grouping of gliomas according to their underlying pathogenic mechanisms may enable the identification of glioma molecular subtypes with unique etiology and thereby facilitate the design and assessment of therapies [25]. Several molecular classification schemes for gliomas have been developed [26–32]. These classification schemes were either restricted to low- or high-grade gliomas [5, 26–28, 33], or based on prognosis-related gene expression signatures [27, 32], or used unbiased analysis of altered genomic patterns, or in the profiles of transcriptome or DNA methylome [28, 30, 31, 33, 34]. Based on the gene co-expression modules around EGFR or PDGFRA, we have previously established the EM/PM classification scheme that classifies all adult diffuse gliomas into three major subtypes with distinct prognosis, unique patterns of genomic alterations and association to cell linage and differentiation stages in neural development [35]. These classification schemes however all fail to distinguish adult diffuse gliomas according to their extents of RTK pathway activities, and to assess the contribution of the glioma microenvironment.

Here, we have developed an alternative approach to cluster adult diffuse gliomas, WHO grades II-IV, according to the overall activities of RTK-related signaling. We identified gene co-expression modules around NF1 (NF1-M), SPRY 1, 2 and 4 (SPRY-M), or PTEN (PTEN-M) in adult diffuse gliomas. The signatures of these co-expression modules enabled robust clustering of adult diffuse gliomas into two subtypes with high or low activities in the RAS-RAF-MEK-MAPK cascade and the PI3K-AKT pathway (we refer to these as RMPA^high^ or RMPA^low^ gliomas). The RMPA clustering is independent of tumor morphology, captures the integral RTK signaling activities in glioma, and demonstrates the contribution of microenvironment-derived RMPA signature to glioma progression. The two RMPA subtypes were associated with distinct prognoses, harbored unique sets of somatic copy number alterations (SCNA) in RTK-related genes, and contained different extents of angiogenic activities and infiltrating immune cells.

## RESULTS

### Clinical impact of RMPA clustering in adult diffuse gliomas

Using Pearson correlation coefficient analysis, we identified gene co-expression modules around SPRY, NF1 and PTEN in the database GSE4290 which includes the transcriptome data from 157 adult diffuse gliomas WHO grades II-IV and 23 epileptic brain samples as controls [36]. Among the top 100 probe sets most closely correlated to SPRY1 (212558_at), 48 common probe sets encoding 26 genes also closely co-expressed with SPRY2 (204011_at) and SPRY4 (221489_s_at) were defined as the SPRY-module (SPRY-M). Similarly, the top 100 most closely co-expressed probe sets to NF1 (212678-at) and PTEN (225363-at), corresponding to 85 and 79 genes respectively, were defined as the NF1-M and PTEN-M (Supplementary Table S1).

**Figure 1:**
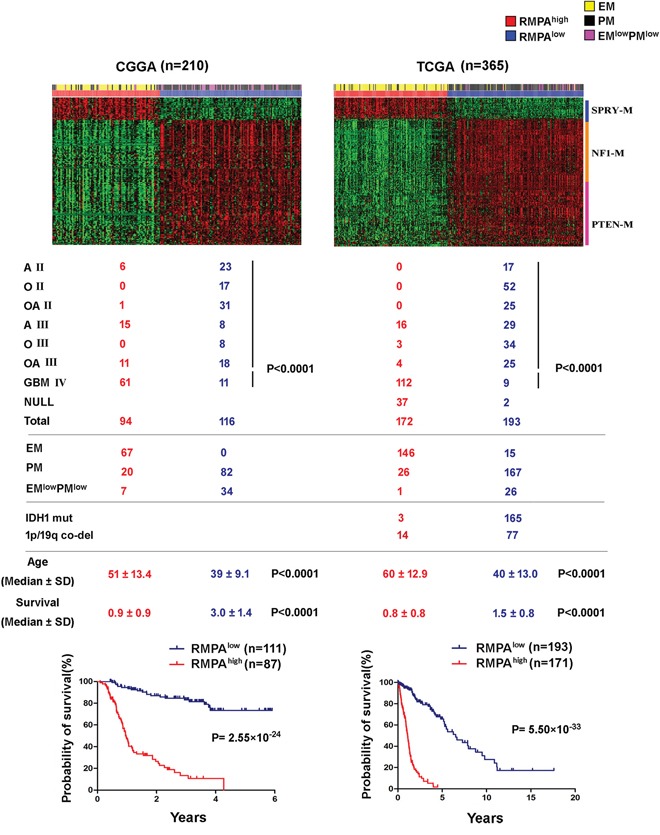
RMPA^high^ and RMPA^low^ gliomas were associated with distinct survival and ages at diagnosis Based on the signatures of SPRY-M, NF1-M and PTEN-M, 575 adult diffuse gliomas of WHO grades II-IV from the CGGA and TCGA mRNA-seq data sets were clustered using NMF. Distribution of morphologically diagnosed glioma subgroups among the RMPA glioma subtypes was analyzed using the Pearson *χ2* test. Patients with RMPA^low^ gliomas showed better survival and younger ages at diagnosis compared to patients with RMPA^high^ gliomas. A II, O II, OA II, A III, O III, OA III and GBM are the abbreviations of morphologically diagnosed astrocytoma grade II, oligodendroglioma grade II, oligoastrocytoma grade II, astrocytoma grade III, oligodendroglioma grade III, oligoastrocytoma grade III and glioblastoma, respectively. Samples with no information of morphological diagnosis from the source data are indicated with NULL.

Based on the signature of these three modules, we used non-negative matrix factorization (NMF [37]) to cluster 1552 adult diffuse gliomas of WHO grades II-IV from five independent data sets. These data sets were based on Agilent, Affymetrix or mRNA-seq platforms and were generated using patient materials from China, the Netherlands and the USA (Supplementary Table S2). Across the data sets, cophenetic coefficient analyses showed that irrespective of the morphological diagnosis, glioma samples were stably and reproducibly clustered into the RMPA^high^ subtype which has high expression of SPRY-M and low expression of NF1-M and PTEN-M, and the RMPA^low^ subtype with a reversed expression pattern of the three modules (Figure 1, Supplementary Figures S1, S2).

All morphological subgroups of adult diffuse gliomas were found in both RMPA^high^ and RMPA^low^ subtypes. There was a significant trend of more GBMs in the RMPA^high^ subtype and more low-grade gliomas in the RMPA^low^ subtype (Figure 1 and Supplementary Figure S2). However, the RMPA^low^ subtype in the REMBRANDT data set contained 29% of the total GBMs (Supplementary Figure S2). In the GSE16011 and the REMBRNDT databases, epileptic brain samples also showed a RMPA^low^ signature. Compared to the RMPA^low^ gliomas, these non-tumor samples showed significantly higher PTEN-M expression and weak SPRY-M expression (Supplementary Table S3), but no measurable difference in NF1-M expression. In three of the four databases with available data on the ages at the time of diagnosis, RMPA^low^ subtype was associated with younger ages (median age < 50 y) compared to the RMPA^high^ subtype. In all data sets analyzed, patients with the RMPA^low^ subtype were associated with a highly significantly longer survival compared to the patients with the RMPA^high^ subtype (Figure 1 and Supplementary Figure S2). Compared with our previous EM/PM classification scheme for adult diffuse gliomas [35], the majority of the EM gliomas and a small fraction of PM or EM^low^PM^low^gliomas showed RMPA^high^ signature, whereas the majority of PM and EM^low^PM^low^ gliomas showed the RMPA^low^ signature.

Using morphological criteria for diagnosis, patients with high-grade gliomas are often associated with poor survival compared with patients with low-grade gliomas, but there is considerable heterogeneity in the survival time of both high and low grade gliomas [1, 2, 28]. In three out of five data sets where reasonable group sizes were available, we found that patients with grade III or IV gliomas of the RMPA^low^ type survived significantly longer compared to patients with grade III or IV gliomas of the RMPA^high^ signature (Supplementary Figure S3).

**Figure 2:**
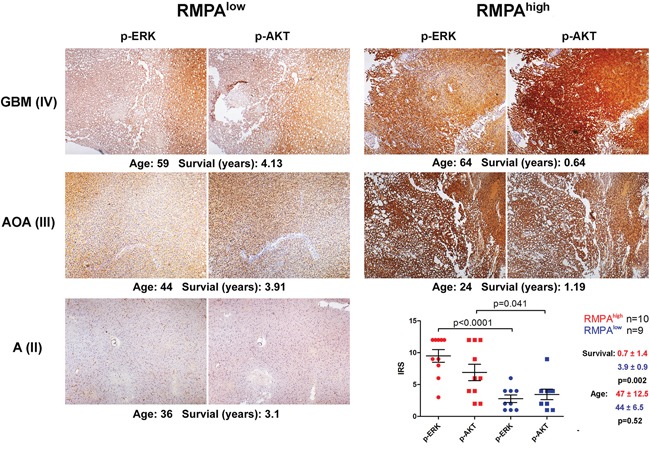
Distinct overall activities in RAS-RAF-MEK-MAPK cascade and PI3K-AKT pathway in RMPA glioma subtypes Formalin-fixed and paraffin-embedded RMPA^high^ (n = 10) and RMPA^low^ (n = 9) glioma samples of the CGGA mRNA-seq database were sectioned and stained for p-ERK and p-AKT. Representative staining results of p-ERK and p-AKT (at a magnification of x40), the staining results summarized as immunoreactive score (IRS), the age at diagnosis and survival of the corresponding patients are shown.

By staining phosphorylated ERK (p-ERK) and phosphorylated AKT (p-AKT) in representative samples of the CGGA mRNAseq data set (n = 19), we confirmed that the RMPA signatures were closely correlated with the striking differences in the activity of the RAS-RAF-MEK-MAPK cascade and the PI3K-AKT pathway. Weak and region-dependent staining of p-ERK and p-AKT was observed in the RMPA^low^ gliomas, whereas RMPA^high^ gliomas showed wide-spread and more intense staining of p-ERK and p-AKT, irrespective of their morphological diagnosis. Within this subset of gliomas, patients with RMPA^high^ gliomas showed significantly shorter survival compared to the patients with RMPA^low^ gliomas (Figure 2).

These findings together suggest that independent of the morphological diagnosis, gliomas with RMPA^high^ or RMPA^low^ signature showed distinct overall RTK signaling activities. Clinically, these two subtypes were associated with distinct patient prognosis and age at the time of diagnosis.

### Distinct genomic alterations in RMPA^high^ and RMPA^low^ gliomas

**Figure 3:**
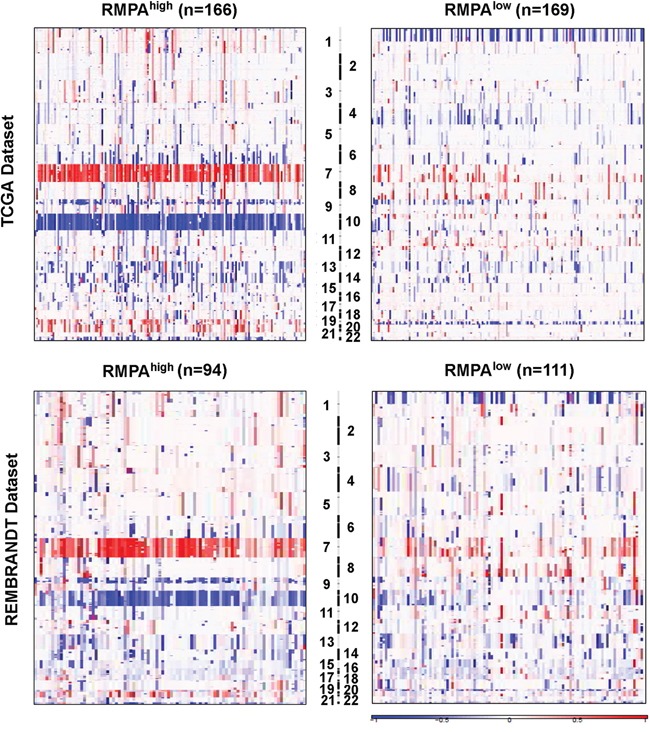
Distinct chromosomal alterations in RMPA^high^ and RMPA^low^ gliomas The SNP6.0 data from 334 gliomas in the TCGA mRNA-seq data set and the 50k *HindIII* SNP array data from 205 gliomas in the Rembrandt data set were analyzed using GISTIC2.0 at an amplitude threshold of ± 0.2. The arm-level chromosomal gaines or losses in the RMPA^high^ and RMPA^low^ gliomas are depicted. In the TCGA mRNA-seq dataset, ~80% of the RMPA^high^ gliomas showed amplification of Chr 7 accompanied with loss of chr 10, and ~50% of the RMPA^low^ gliomas showed co-deletion of chr 1p and chr 19q. A similar trend was observed in the Rembrandt data set.

Based on the GISTIC analysis of the SNP data from 334 gliomas in the TCGA mRNA-seq cohort and 205 gliomas in the REMBRANDT database [4, 35, 38, 39], we characterized SCNAs in the RMPA^high^ and RMPA^low^ gliomas. Arm-level alterations showed that ~80% of the RMPA^high^ gliomas harbored amplification of chromosome 7 coupled with loss of chromosome 10, whereas ~50% RMPA^low^ gliomas harbored co-deletions of chromosome 1p and 19q (Figure 3, Supplementary Tables S4 and S5). According to the residual *q* value of the regional alterations, we identified the top 20 most significantly amplified or deleted peaks in RMPA^high^ and RMPA^low^ gliomas. Regions harboring *EGFR* (7p11.2) or *CDKN2A* (9p21.3) were the most frequently amplified or deleted region in the RMPA^high^ gliomas (Supplementary Tables S4 and S5). The other regions were also distinct or not overlapping between the RMPA^high^ and RMPA^low^ gliomas (Supplementary Table S4 and S5). Thus, gliomas with RMPA^high^ or RMPA^low^ phenotype were associated with distinct patterns of genomic alterations.

To search for genomic alterations potentially causal for the RMPA^high^ signature, we analyzed the SCNA and mutations in the RTKs/RTK ligands, and in the key members of the RAS-RAF-MEK-MAPK cascade and the PI3K-AKT pathway in the TCGA mRNA-seq database (Supplementary Table S6). EGFR is the most frequently altered RTK detected in GBMs [4]. Among the 166 RMPA^high^ gliomas, 88 harbored focal amplification of *EGFR*, and 35 of these *EGFR* amplifications also harbored mutations in *EGFR*; an additional 7 RMPA^high^ gliomas harbored EGFR mutations without detectable focal amplification (Supplementary Table S6). Compared with those RMPA^high^ gliomas without *EGFR* alterations, RMPA^high^ gliomas with *EGFR* alterations showed higher EGFR expression (Supplementary Figure S4). However, unsupervised principle component analysis of the global transcriptome data showed similar transcriptomic profiles between the RMPA^high^ gliomas with or without *EGFR* alterations (Supplementary Figure S5). This demonstrates the important point that alternative mechanisms, which are independent of alterations in *EGFR*, can contribute to the RMPA^high^ signature in those gliomas. Amplifications and mutations of *PDGFRA*, *KIT* and *KDR* were found in 8-15% of RMPA^high^ gliomas, followed by *EPHB3* (7.7%), *FGFR1* (5.9%), *FGFR3* (5.9%) and *MET* (4.1%) (Supplementary Table S6). The RTK ligands including *EFNB2*, *SEMA3D*, *PDGFA*, *SEMA3A* and *FGF14* were amplified in ~5% of the RMPA^high^ gliomas. Losses and mutations of *NF1* and *PTEN*, as well as amplification and mutation of *PIK3CA* were preferentially found in the RMPA^high^ gliomas. Gene-dosage dependent expression was found in association with SCNAs in *EGFR*, *PDGFRA*, *KIT*, *MET*, *NF1*, *PIK3CA* and *PTEN* (Supplementary Table S6).

A tendency towards mutual exclusivity between the loss or mutation of *NF1* and amplification or mutation of *EGFR* was observed using Fisher's exact tests (Supplementary Table S7), indicating that both types of alteration may be sufficient to activate the RAS-RAF-MEK-MAPK cascade in RMPA^high^ gliomas. Co-occurrence between amplification of *EPHB3* and alterations in *PIK3CA* (both at chromosome 3q26-3q27), and amplifications in *PDGFRA*, *KIT* and *KDR* (all at 4q12) were also found in a subset of RMPA^high^ gliomas (Supplementary Table S7), due to their localization in the common amplicons (Supplementary Figure S6). No other mutual exclusivity or co-occurrence was found between the SCNA/mutations analyzed here, indicating independent occurrence of the somatic genomic alterations in other RTK signaling related genes (Supplementary Table S7).

In the RMPA^low^ gliomas, a different set of RTK signaling-related genes including *MET* (8.9%), *EPHA1* (8.9%), *EPHB6* (8.9%), *EPHB4* (8.3%), *BRAF* (8.9%), *FGF6* (11.9%), *FGF23* (11.9%), *NTF3* (11.9%), and *ANGPT1* (9.5%) were amplified (Supplementary Table S6). Using Fisher's exact tests, we found statistically significant co-occurrences of focal amplifications between *MET*, *EPHB4*, *EPHA1*, *EPHB6* and *BRAF* (all at chromosome 7q), between *NTRK1*, *INSRR*, and *SEMA4A* (all at chromosome 1q2), or between *FGF6*, *FGF23*, *NTF3* (all at chromosome 12p13), and *ANGPT1* (at chromosome 8p23.1) (Supplementary Table S8). Further, IGV analysis of the focal amplifications between *MET*, *EPHB4*, *EPHA1*, *EPHB6* and *BRAF* indicate that these amplifications were located in a common amplicon (Supplementary Figure S6). The amplifications of *BRAF*, *EPHB4*, *FGF23* and *INSRR* were associated with gene-dosage related changes in expression (Supplementary Table S6).

Genes co-expressed in the same module tend to be co-regulated by common mechanisms [40, 41]. We found that 32 of 79 PTEN-M members are located at chromosome 10, their loss in 86%-90% of the RMPA^high^ gliomas caused a significant gene dosage-dependent reduction in gene expression and potential modulating effect of the entire PTEN-M expression. In particular, *PTEN* locus was lost in 147 of the 166 RMPA^high^ gliomas, resulted in a highly significant gene dosage-dependent expression (Supplementary Table S1). Further, in 30 RMPA^high^ gliomas with heterozygous *PTEN* loss, the remaining allele was also mutated. Similarly, 16 of the 85 NF1-M members located at chromosome 10 were lost at similar frequencies as the loss of PTEN-M members in the RMPA^high^ gliomas, resulting in gene dosage-dependent down-regulation of expression with a significant potential to influence the expression level of the entire NF1-M. Further, 21 and 10 RMPA^high^ gliomas harbored heterozygous and homozygous loss of *NF1*, respectively; 10 additional RMPA^high^ gliomas harbored *NF1* mutation without loss of *NF1* locus. Seven additional PTEN-M genes and 12 additional NF1-M genes are located in frequently lost chromosomal regions (13q, 14q and 22q, Supplementary Table S1). Thus, recurrent loss of chromosomal regions and frequent mutations in *PTEN* and *NF1* strongly contributed to the weak expression and functional loss of NF1-M and PTEN-M in the RMPA^high^ glioma subtype. SPRY-M activity appeared mainly to be a consequence of an active feedback to elevated MAPK activity in gliomas, as the high SPRY-M signature was seen both in gliomas where members of the SPRY-M were amplified as well as in those in which there was loss of these genes (Supplementary Table S1). For example, in 54 of 166 RMPA^high^ and in 30 of 168 RMPA^low^ gliomas from the TCGA mRNA-seq data set one genomic copy of *SPRY2* was lost. Nevertheless, the expression of SPRY2 in these 54 RMPA^high^ gliomas was on average 2-fold higher than in the 30 RMPA^low^ gliomas (p =3.2 × 10^−6^, *t* test). This is significant since the SPRY-M members are involved at various levels in MAPK activity, cell proliferation, cell adhesion, Rho protein signaling transduction and signaling in angiogenesis or apoptosis.

In addition, the vast majority of the gliomas with *IDH1* mutation were found in the RMPA^low^ subtype. Further analyses of the GBM samples reported by Brennan *et al.* [4] showed that all classical and mesenchymal GBMs, and the majority of neural or proneural GBMs showed RMPA^high^ phenotype. A small proportion (<15%) of the neural or proneural GBMs showed RMPA^low^ phenotype. These results together show that, unique sets of RTKs and RTK-signaling genes were recurrently altered in the RMPA^high^ and RMPA^low^ gliomas. Mutual exclusivity was found between the alterations in *EGFR* and *NF1*, co-occurrences of SCNAs in RTK-signaling genes were due to their location in the common amplicons. Further, recurrent genomic losses in the members in PTEN-M and NF1-M directly caused their weak expression in the RMPA^high^ gliomas.

### Contribution of glioma microenvironment to RMPA signature

Previous characterizations of dysregulated RTK pathway activities in gliomas have predominantly focused on the genomic alterations in RTK pathway genes [3, 4]. By comparing the expression pattern of all 63 RTKs and 82 RTK ligands in the human genome between the gliomas with RMPA^high^ or RMPA^low^ phenotype, we analyzed the expression of RTKs and their ligands in a manner irrespective of their genomic alterations. At a statistical significance of p = 10^−6^ and q values ranging between 2.0 × 10^−6^ and 3.9 × 10^−7^ (and concordant expression pattern in at least four data of the five data sets analyzed), the ligands to EGFR (AREG with > 10-fold higher expression in the RMPA^high^ gliomas, and EGF) were found enriched in the RMPA^high^ gliomas, which may have contributed to enhanced EGFR signaling in RMPA^high^ gliomas irrespective of *EGFR* alteration. Besides VEGF-related pro-angiogenic factors (VEGFA with ~ 9-fold higher expression in the RMPA^high^ gliomas, and VEGF co-receptor NRP1 and NRP2), high expression of additional pro-angiogenic factors including ANGPT1 and ANGPT2, EPHA2/EPHB2, EFNB2/EPHB4, PDGFA/PDGFD/PDGFRB and SEMA3A/SEMA3F/PLXNA3/PLXNB2 [42] was found in RMPA^high^ gliomas. RMPA^high^ gliomas were also enriched in the expression of ERBB2, FGFR1 and MET (and its ligand HGF), DDR2 (a receptor for activated collagen fibers [43]), as well as the WNT co-receptors ROR1 and RYK [44]. Unlike the RMPA^high^ gliomas, the RMPA^low^ gliomas showed enriched expression of ERBB3, ERBB4, FGF9, FGF12, FGF13, FGF14, NTRK2, NTRK3, together with a different set of ephrins and Eph receptors (EFNA3/EPHA10, EFNB3/EPHB6, EPHB1), as well as members of SEMA3/SEMA4 family and PLXNB family (Supplementary Figure S7 and Supplementary Table S9). At a relatively lower statistical significance (p and q values at the range of 1.0 × 10^−3^, and concordant expression pattern in at least three data sets), CSF1 (important for differentiation and survival of TAM [21]) and KDR (VEGFRII) were found enriched in the RMPA^high^ gliomas; and FGFR2 and PDGFRA in RMPA^low^ gliomas.

We next aimed to map the RTK expression to the major cell types in glioma. Twenty-two fresh glioma samples were profiled for their transcriptome using human gene 1.0 ST array (GSE74462) and in parallel prepared as single living cells for assessing cell surface RTK expression. Consistent with the findings in the other data sets, RMPA^high^ and RMPA^low^ gliomas were identified based on the signature of SPRY-M, NF1-M and PTEN-M. Though the cohort size was limited, patients with gliomas of RMPA^high^ signature were associated with poor survival compared with patients with RMPA^low^ gliomas (Supplementary Figure S8).

**Figure 4:**
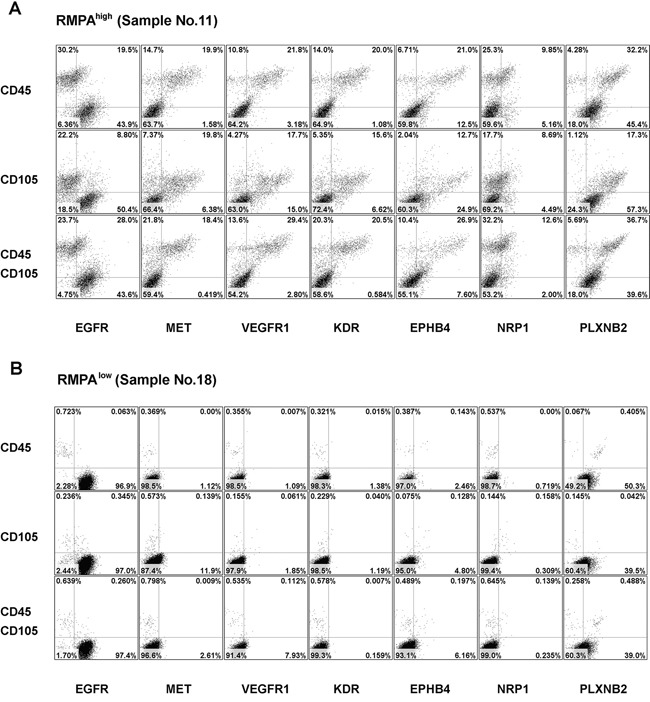
Enriched expression of angiogenic RTKs in vessel endothelial cells and infiltrating immune cells in RMPA^high^ gliomas Single cells from RMPA^high^ or RMPA^low^ gliomas were co-stained with APC-conjugated anti-CD45 or anti-CD105 mAbs, in combination with one of the PE-conjugated anti-RTK mAbs, or with isotype-matched control antibodies. Dot plots of the bottom rows were the results of co-staining of APC-conjugated anti-CD45 and anti-CD105 mAbs together with one of the indicated PE-conjugated anti-RTK mAb. Living cells excluding 7-AAD staining were gated and analyzed for the co-expression of CD45, CD105 and RTKs. Dot-plots of representative RMPA^high^ **A.** or RMPA^low^ glioma sample **B.** are presented. The expression of EGFR and PLXNB2 was seen in CD45^−^CD105^−^ cells in both RMPA^high^ or RMPA^low^ gliomas. The expression of MET, VEGFR1, KDR, EPHB4 and NRP1 was observed in both CD45^+^ and CD105^+^ cells, but not in CD45^−^CD105^−^ glioma cells. Results of isotype control and other control stainings are depicted in Supplementary Figure S10.

Cells from these RMPA^high^ or RMPA^low^ gliomas were co-stained with APC-conjugated mAb against CD45 (for labeling infiltrating immune cells [45]) or CD105 (for labeling immature proliferating vessel endothelial cells [46]), and one of the PE-conjugated mAb for RTK. Glioma cells were enriched in the cells with CD45^−^CD105^−^ immunophenotype. High levels of EGFR (positive in > 10% of the total living cells) in the CD45^−^CD105^−^ cells was detected in 6 of 6 RMPA^high^ and 13 of 16 RMPA^low^ gliomas analyzed; and high PLXNB2 expression in the CD45^−^CD105^−^ cells was detected in 5 of 6 RMPA^high^ and 11 of 16 RMPA^low^ gliomas analyzed. In gliomas with the RMPA^high^ signature, numerous CD45^+^ immune cells and CD105^+^ immature endothelial cells were found (Supplementary Table S10, Supplementary Figure S9). In these RMPA^high^ gliomas, angiogenic related RTKs including MET, VEGFR1, KDR, EPHB4, NRP1 were frequently and concomitantly expressed in CD45^+^ immune cells and CD105^+^ endothelial cells. This is in agreement with a previous report on enriched expression of angiogenesis promoting molecules in TAMs [47]. MET expression in the CD45^−^CD105^−^ cells was detected in 1 of the 6 RMPA^high^ gliomas analyzed. Heatmap in Supplementary Figure S8 showed that this sample (N9) was not a borderline sample between the RMPA^high^ and RMPA^low^ subtypes. Further, N9 showed high expression of EGFR, MET, VEGFR1, KDR, EPHB4, NRP1, PLXNB2 in CD105^+^ cells (Supplementary Figure S9B). Though gliomas with RMPA^low^ signature contained fewer CD45^+^ cells and CD105^+^ cells, CD45^+^ cells in these gliomas also expressed VEGFR1, KDR, EPHB4 and PLXNB2 (Figure 4, Supplementary Figures S9B, S10 and Supplementary Table S11).

**Figure 5:**
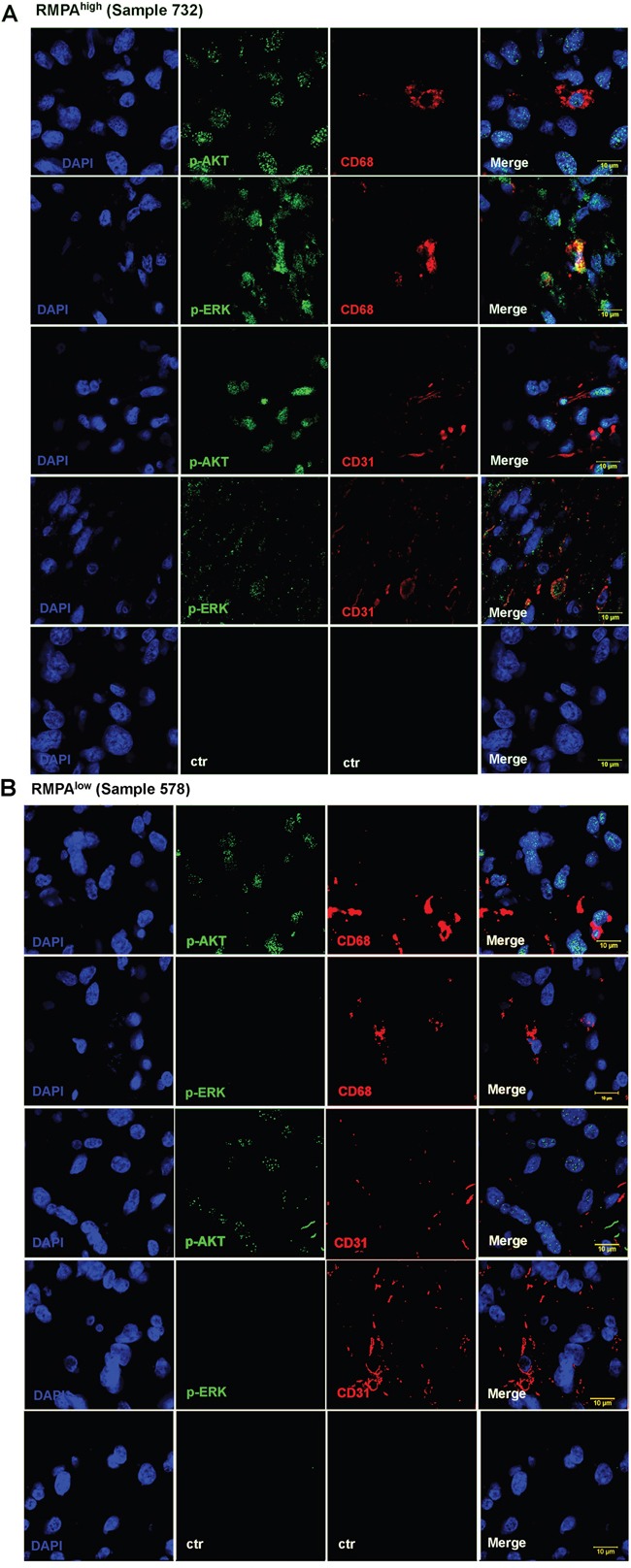
Activation of RTK signaling in vessel endothelial cells and TAMs in RMPA^high^ gliomas Sections of the classified RMPA^high^ or RMPA^low^ gliomas were co-stained with mAbs against CD31 or CD68 in combination with mAbs towards p-AKT or p-ERK. The staining of anti-CD31 or anti-CD68 was detected with Alexa Fluor^®^ 555 conjugated secondary antibody, and anti-p-AKT or anti-p-ERK with Alexa Fluor^®^ 647 conjugated secondary antibody. Sections were further stainined with DAPI and evaluated using a confocal microscope (ZEISS LSM 700). Images of representative RMPA^high^ **A.** or RMPA^low^. **B.** gliomas are shown. The control stainings were performed in the absence of the primary antibody. Staining results of other RMPA classified or non-classified samples are summarized in Supplementary Figure S11.

We also co-stained sections of RMPA^high^ or RMPA^low^ gliomas with CD31 (a marker for vessel endothelial cells) or CD68 (a marker for TAM) with antibody to p-ERK or p-AKT. Confocal analyses of the co-staining showed that in addition to glioma cells, high RTK signaling activities were detected in vessel cells and infiltrating TAMs in RMPA^high^ gliomas; weak or barely detectable p-AKT activity in CD68^+^ or CD31^+^ cells was observed in RMPA^low^ gliomas (Figure 5, Supplementary Figure S11). These results together show that RMPA signature is an integral output of the RTK pathway activities derived from glioma cells, vessel cells and infiltrating immune cells in glioma microenvironment.

### Impact of RMPA signature in glioma progression

**Figure 6:**
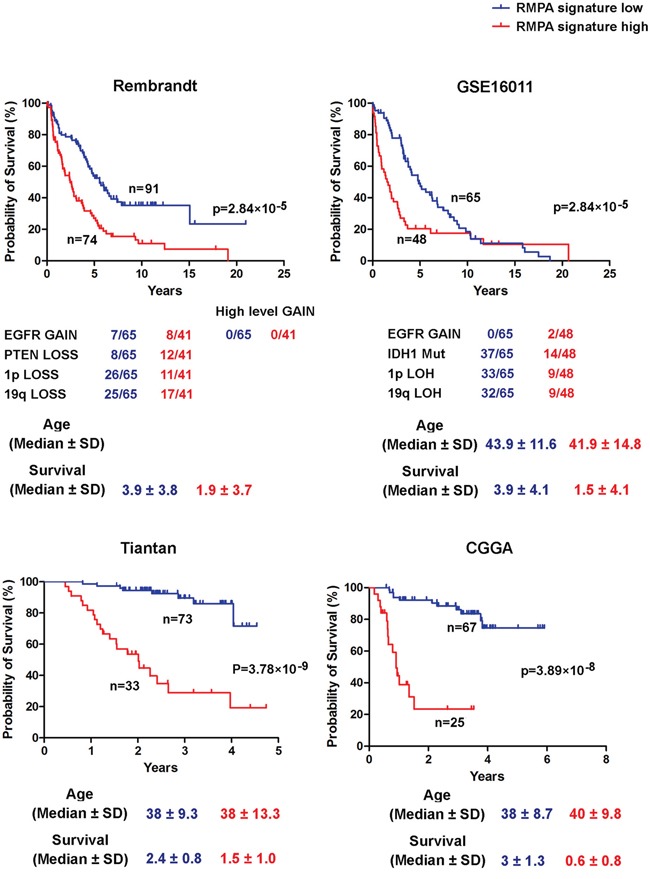
Poor prognosis of PM gliomas with high RMPA signature Unsupervised hierarchical clustering between RMPA classifiers and PM gliomas was performed on all data sets analyzed. The data on copy number alterations of *EGFR* and *PTEN*, co-deletion of 1p and 19q, *IDH1* mutation, and the age at the diagnosis are presented. In four indicated data sets, PM gliomas with a high RMPA signature showed poor overall survival compared with PM gliomas with a low RMPA signature.

We further assessed the impact of RTK signaling derived from vessel cells and infiltrating TAM in our previously defined PM gliomas [35]. IDH1 mutation and co-deletion of chromosome 1p19q, but not the alterations in RTK pathway genes, are the predominant form of somatic genomic alterations in PM gliomas [35]. In four data sets, unsupervised hierarchical clustering ordered PM gliomas into two subgroups, one with a relatively high and the other with a relatively low RMPA signature (Supplementary Figure S12). Significantly poorer prognosis was reproducibly found in patients with gliomas of relatively higher RMPA signature (Figure 6). Copy number analysis of the 100 K SNP data in the REMBRANDT dataset detected only low-level amplification of *EGFR* in both subsets of PM gliomas and almost no PM gliomas in GSE16011 harbored *EGFR* amplification. Thus, a relatively high RMPA signature in PM gliomas may most likely originate from cells infiltrating the tumor microenvironment. These findings show that accessory cell-derived RTK signaling correlated with accelerated progression of glioma.

## DISCUSSION

Since cellular morphology is predominantly controlled by the Rho GTPase dependent actin cytoskeleton remodeling [48], each of the morphologically defined glioma subgroups may contain more than one molecular subtype. The unsatisfactory efficacies of RTK targeting therapies may have been caused by factors including treatment of unselected glioma patients, co-activation of multiple RTKs or alternative pathways for elevated RTK-related signaling [4, 10, 15–17]. Unlike previously established glioma classification schemes, in which RTK-related genomic alterations could not be analyzed in a glioma molecular subtype-dependent manner [5, 27, 29–31, 33, 34], or showed preferential, yet overlapping enrichment between the subtypes [26], unique sets of genomic alterations in RTK signaling-related genes were found in the RMPA^high^ and RMPA^low^ gliomas. The genomic alterations in *EGFR*, *PDGFRA*, *NF1*, *PTEN* and *PIK3CA* were nearly exclusively found in the RMPA^high^ gliomas. Due to an amplicon at chromosome 7q, about 9% of the RMPA^low^ gliomas harbored amplifications of *MET, EPHA1, EPHB4, EPHB6*, and *BRAF.* Another amplicon at 12p13 in 12% of the RMPA^low^ gliomas resulted in co-amplifications of *FGF6*, *FGF23* and *NTF3*. A third amplicon at 1q22 to 1q23 resulted in co-amplification of *NTRK1, INSRR* and SEMA4A. These three amplicons occurred independently in the RMPA^low^ gliomas and did not enhance the activities of RAS-RAF-MEK-MAPK cascade or PI3K-AKT pathway to levels similar to those seen in the RMPA^high^ gliomas. Irrespective of these inter-individual variabilities in the genomic alterations among the RTKs and RTK signaling genes, low NF1-M expression and up-regulated SPRY-M expression reflected elevated Ras-MAPK signaling, and loss of PTEN-M expression indicated the activation of PI3K-AKT pathway; these three signatures in combination enabled the distinction of adult diffuse gliomas into the RMPA^high^ and RMPA^low^ subtypes according to the overall activity of RAS-RAF-MEK-MAPK cascade and PI3K-AKT pathway in a morphology independent manner.

In addition to the high frequencies of genomic alterations in RTKs and RTK signaling genes in the RMPA^high^ gliomas, our findings suggest that the overall RTK-signaling activity in gliomas is not necessarily tumor cell autonomous but rather represents an integrated output generated by all of the cell types within the glioma niche. Previous studies have reported that gliomas with poor prognosis contain a high content of infiltrating TAM [21]. A paracrine CSF1-EGF signaling loop implicated in glioma invasion has been established between glioma cells and TAMs [19]. While glioma cell-derived CSF1 plays a critical role for the differentiation and survival of TAMs, TAM derived EGF may contribute to the high EGFR signaling in the gliomas cells, even in the absence of mutations within the *EGFR* gene or amplification of this region. This agrees with our findings that the global transcriptomic profiles between RMPA^high^ gliomas with or without *EGFR* alteration were highly similar, and that EGFR ligand AREG and EGF were enriched in the RMPA^high^ gliomas. TAMs are enriched in the RMPA^high^ gliomas and they show high surface expression of MET, VEGFR1, KDR, EPHB4 and NRP1. The RMPA^high^ gliomas contain high numbers of CD105^+^ immature angiogenic vessel cells. In line with the RMPA^high^ signature, the pro-angiogenic RTKs detected on the surface of TAMs were also highly expressed in CD105^+^ immature vessel cells. The high level expression of these RTKs and their ligands, together with the results of nuclear staining of p–ERK and p-AKT in CD68- or CD105-positive cells, suggest concomitant activation of RAS-RAF-MEK-MAPK cascade and PI3K-AKT pathway in both TAMs and immature vessel endothelial cells in RMPA^high^ gliomas.

Our findings suggest that the cellular origin of elevated RTK signaling should be taken into consideration in the application of therapies targeting RTK-related signaling. For example, previous studies have suggested co-activation of RTKs including EGFR and MET in GBM samples [17], we also see high MET expression in RMPA^high^ gliomas, with about 5% of the RMPA^high^ gliomas harboring *MET* amplification. However, when the surface expression of MET was assessed at the single cell level, we observed a promiscuous pattern of MET expression in CD45^+^, CD105^+^ and CD45^−^CD105^−^ cell populations. Cell surface MET protein was more frequently expressed in the infiltrating immune cells and vessel endothelial cells than in glioma cells. These findings indicate that in considering therapeutic options it will be necessary to determine the relative contribution to tumor growth of the glioma cells on the one hand and the infiltrating TAMs and vessel cells on the other, as shown in our analyses of the PM gliomas [35], RTK signaling in the accessory cells may drive the progression of gliomas in which alterations in the RTK-related genes are not predominant genomic alterations.

In summary, we have identified gene signatures robustly co-expressed around NF1, SPRY or PTEN, enabling distinction of adult diffuse gliomas of WHO grades II-IV into RMPA^high^ and RMPA^low^ subtypes. Identification of RMPA^high^ and RMPA^low^ gliomas may facilitate the design and assessment of RTK signaling-related therapies directed at glioma cells or at the important accessory cells promoting glioma growth and progression. This may not only help to clarify the pathobiology of gliomas but also provide patient-personalised, rational criteria for identifying possible therapeutic interventions.

## MATERIALS AND METHODS

### Identification of co-expression modules, clustering of gliomas and survival analysis of patients with RMPA^high^ or RMPA^low^ gliomas

Qlucore Omics Explorer 3.0 (Qlucore AB, Lund, Sweden) was used for gene co-expression module construction and differential gene expression analysis. Using Pearson correlation coefficient analysis in glioma gene expression database GSE4290 including the transcriptome data from 157 adult diffuse gliomas WHO grades II-IV and 23 epileptic brain samples [36], we first identified the top 100 most correlated probe sets to SPRY1 (212558_at), SPRY2 (204011_at) or SPRY4 (221489_s_at). Forty-eight common probe sets encoding 26 genes were defined as SPRY-M. Similarly, the top 100 most closely co-expressed probe sets to NF1 (212678-at) and PTEN (225363-at), corresponding to 85 and 79 genes, respectively, were identified as NF1-M and PTEN-M (Supplementary Table S1). The list of RTK and RTK ligands were downloaded from KEGG.

We used non-negative matrix factorization (NMF [37]) to analyze the expression signature of NF1-M, PTEN-M and SPRY-M in glioma data sets. At k = 2, stable clusters with high cophenetic coefficient were reproducibly obtained across the data sets. The Kaplan-Meier survival curves for the overall survival time were generated and analyzed with the log-rank test using Prism 5.0 software.

### Immunohistochemical staining

Five μm sections were prepared from formalin fixed and paraffin embedded glioma samples. Following de-waxing and hydration, the sections were pretreated for 10 min at room temperature with 3% H_2_O_2_ to block endogenous peroxidase. Antigen retrieval was performed by microwave boiling of the samples for 15 min in 0.1 M sodium citrate buffer at pH6.0. Incubation with phospho-p44/42 Erk1/2 (Thr202/Tyr204) rabbit mAb (20G11, 1:400 dilution; Cell Signaling) and phospho-Akt (Ser473) rabbit mAb (D9E, 1:50 dilution; Cell Signaling) was performed in PBS at 37°C for 1 hr. The Power-Stain 2.0 Kit Poly HRP (Genemed) was used as the secondary reagent. Staining was developed with DAB. Slides were counterstained with heamatoxylin and mounted.

The staining was evaluated by two independent observers. All cells on the section were considered to determine the immunoreactive score (IRS). The percentages of positive cells ranged from 1 to 4 (1: <10%; 2: 10-50%; 3: 51-80%; 4: >80%) and the staining intensity was scored from grade 0 to 3 (0: negative; 1: weak; 2: moderate; 3: strong staining). The product of the percentage score and intensity score gave the IRS (0-12). Unpaired *t* test was used to compare the IRS values between the RMPA^high^ and RMPA^low^ gliomas.

### Immunoflourescence staining

Five μm sections were prepared from formalin fixed and paraffin embedded glioma samples. After de-paraffinized, the slides were subjected to heat-induced antigen retrieval using citrate buffer (0.1 M, pH6.0) in a pressure cooker for 2 min [49]. The slides were co-stained with rabbit anti-p-ERK (Thr202/Tyr204, D13.14.4E, 1:200 dilution; Cell Signaling) and mouse anti-CD31 (89C2, 1:3200 dilution; Cell Signaling), or anti-p-ERK and mouse anti-CD68 (PG-M1, 1:100 dilution; Dako), or anti-p-AKT (Ser473, D9E, 1:200 dilution; Cell Signaling) and anti-CD31, or anti-p-AKT and anti-CD68. Alexa Fluor^®^ 555 conjugated goat anti-rabbit IgG (Life Sciences) was used in detecting anti-p-ERK and anti-p-AKT, Alexa Fluor^®^ 647 conjugated Goat anti-Mouse IgG (life) was used in detecting anti-CD31 and anti-CD68. Sections were further stained with DAPI and evaluated with a confocal microscope (ZEISS LSM 700).

### Analyses of SCNA, gene dosage effect and co-occurrence or exclusivity between the SCNAs

The level III SNP6.0 data of 349 gliomas were downloaded from the TCGA. The normalized data from gliomas samples and their somatic controls were grouped according to the RMPA classification, and processed using GISTIC2.0 at an amplitude threshold t = 0.2 as implemented in the web application of Genepattern. The data of 100 K SNP array from the Rembrandt data set (*HindIII* part) were normalized and segmented as in our previous report [35], and analyzed in GISTIC2.0 at an amplitude threshold t = 0.2. To determine the level of copy number gain or loss in the subsets of PM gliomas shown in Figure 6, we also analyzed the raw array data using NEXUS COPY NUMBER™ 7.5 (Biodiscovery Inc.). The implemented call algorithm was SNP-FASST2 (Fast Adaptive States Segmentation Technique) segmentation. It first calculated the log_2_ ratio of each probe across the whole genome and then arranged the ratios according to the probe position along the chromosome for each sample. The FASST2 algorithm was used to segment the genomes into regions of uniform ratios. Finally the copy numbers of each region were inferred according to their log_2_ ratios. The log_2_ ratio threshold for gain, loss, high level gain and homozygous loss were 0.25, −0.2, 0.7 and −1.1, respectively. The gain and loss refer to single copy gain and single copy loss, respectively; while high level gain indicates gain of two or more copies.

The gene dosage effect on the expression of the members in NF1-M, PTEN-M and SPRY-M was analyzed in the TCGA mRNA-seq cohort in 169 RMPA^high^ and 169 RMPA^low^ gliomas. Spearman's rank correlation analysis on the data of gene expression and gene dosage was performed. Odds ratio and Fisher's exact test were used to analyze the correlation among the RTK signaling-related SCNAs.

### Measurement of transcriptome data and flow cytometry analysis for cell subtype-specific RTK expression in glioma samples

Glioma samples were divided into two portions, either snap-frozen in liquid nitrogen for transcriptome analysis or prepared as single cells for flow cytometry analysis. For transcriptome analysis with the Human Gene 1.0 ST array, the preparation of total RNA, synthesis of first-strand cDNA, second strand cDNA, cRNA and second cycle cDNA were performed using Ambion WT expression kit, the labeling and hybridization were performed using Affymetrix GeneChip WT terminal labeling kit. The data were analyzed with RMA using Affymetrix default analysis settings and global scaling as normalization method. The trimmed mean target intensity of each array was arbitrarily set to 100. The data have been submitted to NCBI Gene Expression Omnibus (GEO; http://www.ncbi.nih.gov/geo) under accession number GSE74462. The RMPA signature was analyzed using Qlucore software.

For single cell preparation, the portion of glioma specimens was cut finely into small pieces, treated in Iscove's Modified Dulbecco's Medium (IMDM) with 0.5 mg/ml collagenase (Sigma) and 25 mg/ml DNAse (Sigma) at 37°C for 40 minutes. Red cells were lysed with NH_4_Cl. Remaining cells were washed in PBS containing 2% FCS. If not directly analyzed by flow cytometry, cells were resuspended in freezing medium (IMDM containing 10% DMSO and 90% FCS) and stored in liquid nitrogen. About 5 to 10 x10^6^ fresh or thawed cells were first incubated with non-specific blocking mouse IgG1 at 50 mg/ml (clone MOPC 21, Sigma) and Fc Receptor Blocking Solution (Human TruStain FcX™, product 422302, Biolegend) at 4°C for 20 minutes. About 5×10^5^ cells were then stained with allophycocyanin (APC)-conjugated anti-CD45 (clone H130, Biolegend), or anti-CD105 (clone 43A3, Biolegend) monoclonal antibodies (mAb) in combination with a phycoerythrin (PE)-conjugated mAb against one of the RTKs including PDGFRA (clone 16A1, Biolegend), EGFR (clone AY13, Biolegend), EPHA2 (clone 371805, R&D), EPHB4 (clone 395810, R&D), MET (clone 95106, R&D), ERBB2 (clone Neu 24.7, BD), PLXNB2 (clone 537223, R&D), VEGFR1 (clone 49560, R&D), KDR (clone 89106, R&D), NRP1 (clone12C2, Biolegend), at saturating concentrations or the isotype-matched control mAbs at 4°C for 15 minutes. For characterizing CD45^+^ immune cells, cells were stained with APC-conjugated anti-CD45 in combination with a PE-conjugated mAb against CD14 (clone M5E2, Biolegend), or CD4 (clone RPA-T4, Biolegend), or CD8 (clone HIT8a, Biolegend), or CD19 (clone HIB19, Biolegend). Subsequently, cells were washed once with PBS and resuspended in 500 μl PBS supplemented with 2% FCS and 1.0 mg/ml 7-aminoactinomycin D (7-AAD, Sigma). At least 50 000 events were counted in a FACScalibur and cell surface expression was analyzed in 7-AAD negatively stained living cells using Flowjo program. The flow-cytometry analysis was replicated at least twice for each specimen. The use of glioma samples was approved by the Ethics Committee of Beijing Tiantan Hospital, and written informed consent was obtained from all patients.

## SUPPLEMENTARY FIGURES AND TABLES




