# Supplementary material for: Co-expression modules of NF1, PTEN and sprouty enable distinction of adult diffuse gliomas according to pathway activities of receptor tyrosine kinases
Source: Oncotarget. 2016 Jul 1;7(37):59098–114. doi: 10.18632/oncotarget.10359 (PMC5312298; doi:10.18632/oncotarget.10359)
Supplement: Supplementary file 1 [file oncotarget-07-59098-s001.pdf]

Co-expression modules of NF1, PTEN and sprouty enable distinction of adult diffuse gliomas according to pathway activities of receptor tyrosine kinases

SUPPLEMENTARY FIGURES AND TABLES

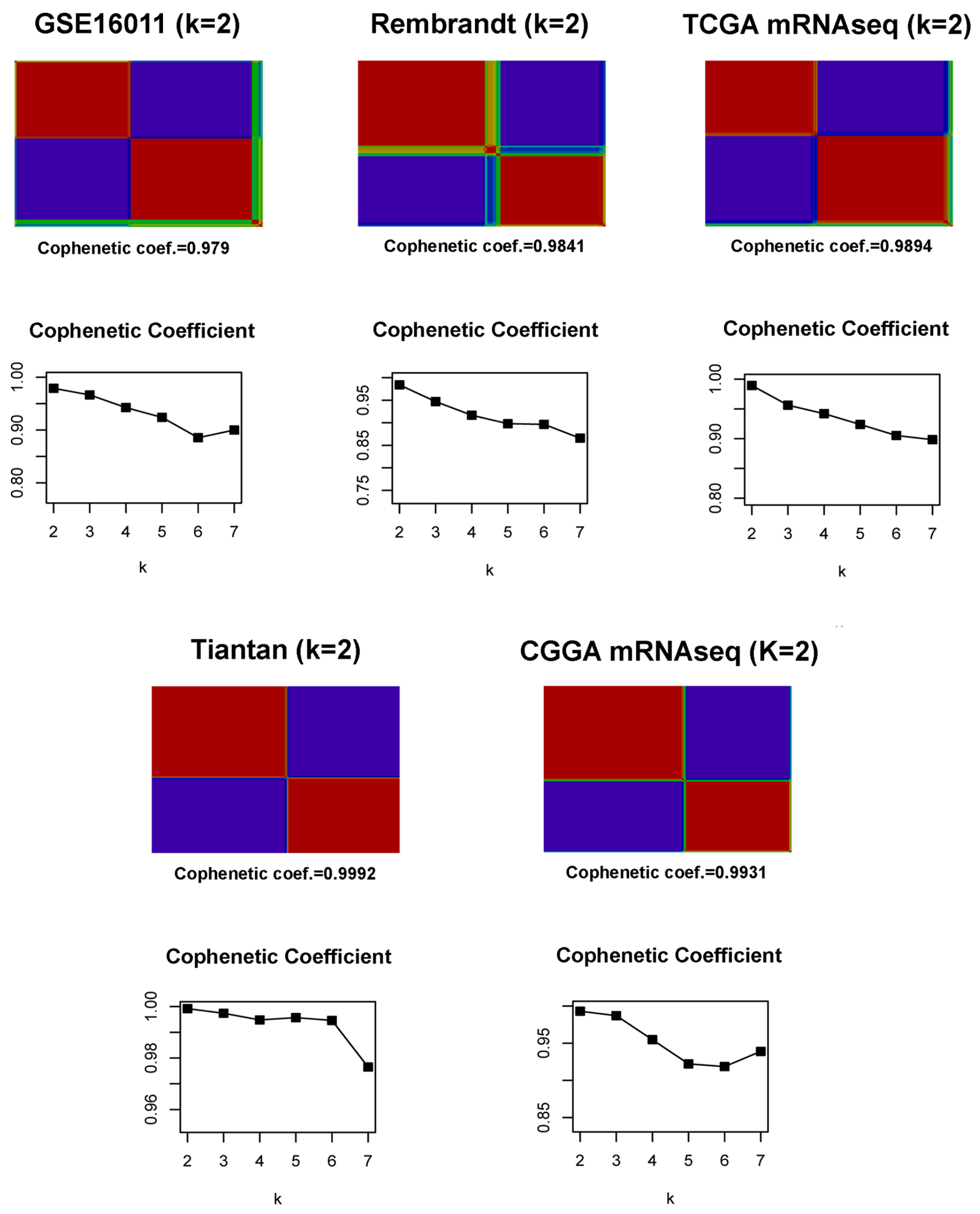

Supplementary Figure S1: Identification of RMPA^{high} and RMPA^{low} glioma molecular subtypes. Gliomas were clustered using NMF based on the expression signatures of NF1-M, PTEN-M and SPRY-M in each of the five databases. The matrix of consensus clustering and cophenetic coefficients for the clusters are shown. At K = 2, adult diffuse gliomas of WHO grades II-IV across these data sets were stably and reproducibly clustered into the RMPA^{high} and RMPA^{low} subtypes.

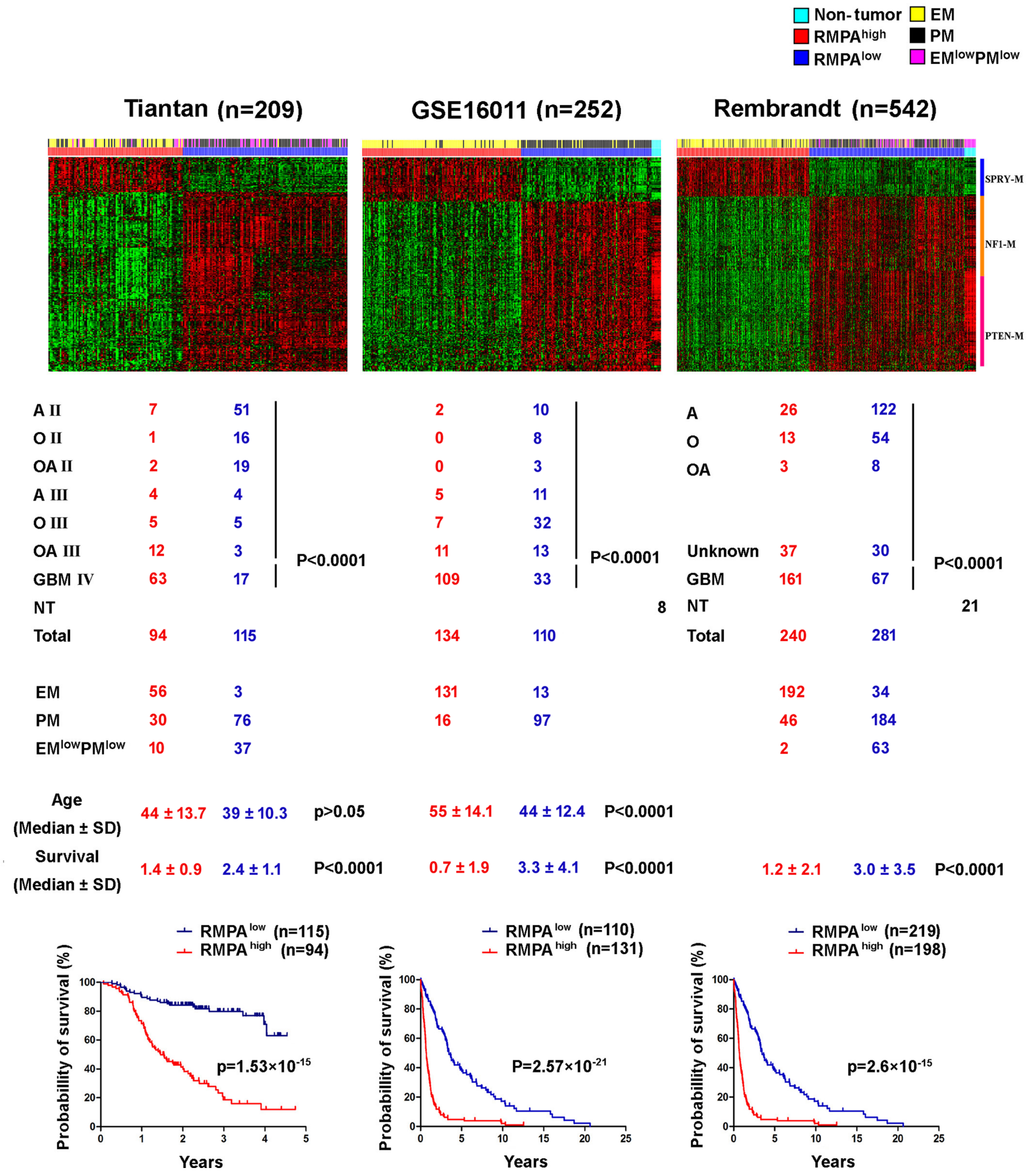

Supplementary Figure S2: RMPA^{high} and RMPA^{low} gliomas were associated with distinct survival and ages at diagnosis. Based on the signatures of NF1-M, PTEN-M and SPRY-M, 1003 adult diffuse gliomas of WHO grades II-IV from the indicated databases were classified using NMF. Non-tumor brain samples in the GSE16011 and the Rembrandt databases showed higher PTEN-M and weaker SPRY-M signatures compared with the RMPA^{low} gliomas. Distribution of morphological diagnosis among RMPA glioma subtypes was analyzed using the Pearson χ^2 test. All morphologically diagnosed glioma subgroups were identified in both RMPA^{high} and RMPA^{low} subtypes, with significantly more GBM in the RMPA^{high} subtype and more low-grade gliomas in the RMPA^{low} subtype. Patients with RMPA^{low} gliomas showed better survival compared to patients with RMPA^{high} gliomas. The data on the ages at the diagnosis was not available for the REMBRANDT data set. A II, O II, OA II, A III, O III, OA III and GBM are the abbreviations of the morphologically diagnosed astrocytoma grade II, oligodendroglioma grade II, oligoastrocytoma grade II, astrocytoma grade III, oligodendroglioma grade III, oligoastrocytoma grade III and glioblastoma, respectively. Non tumor brain samples are indicated as NT.

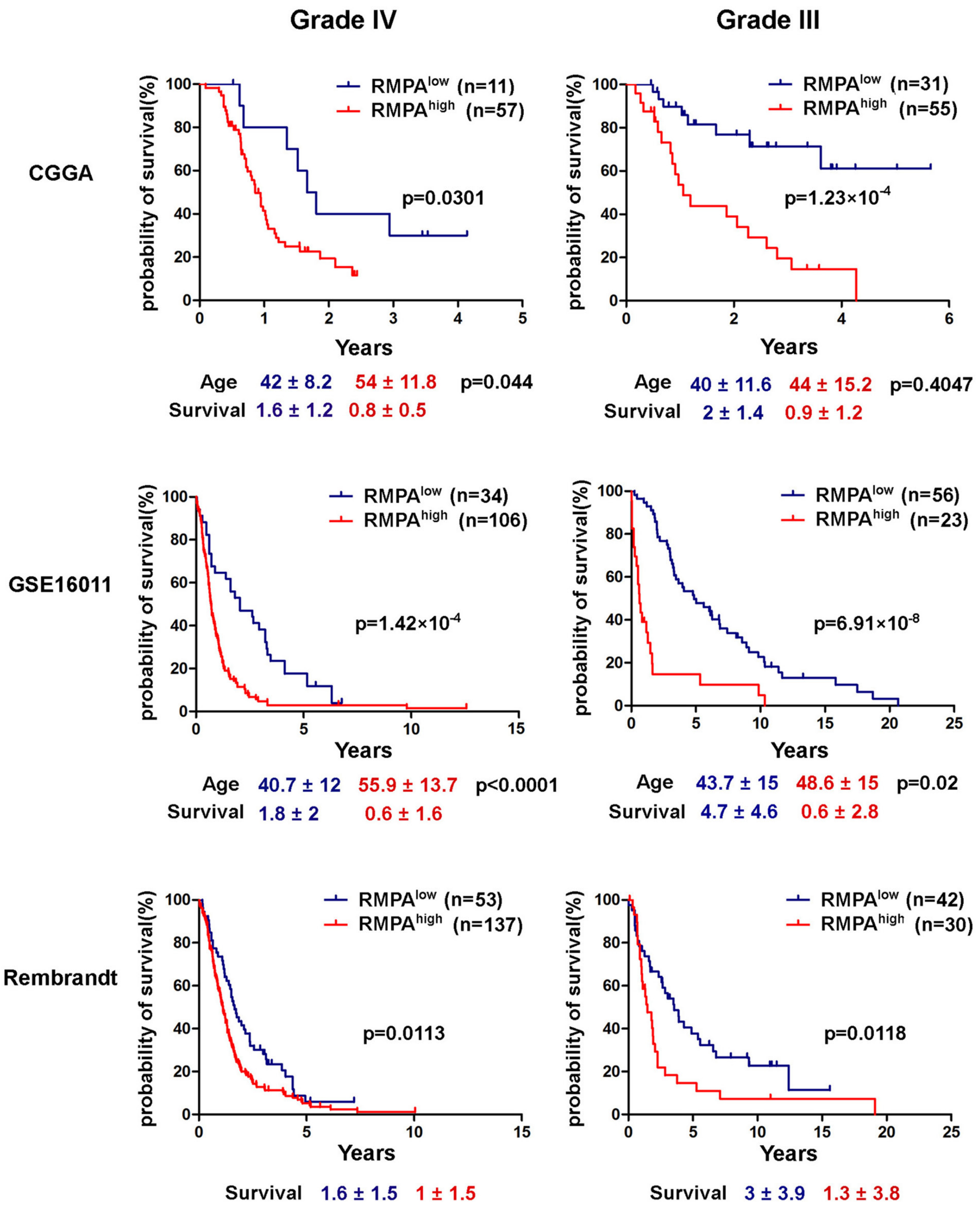

Supplementary Figure S3: High-grade gliomas with RMPA^{high} or RMPA^{low} signatures were associated with different survival outcome. The survival outcome of patients with grade III or IV gliomas was reanalyzed according to the RMPA clustering results shown in Figure 1 and and Supplementary Figure S2. In the CGGA mRNA seq, GSE16011 and Rembrandt data sets, patients with grade III or IV gliomas of RMPA^{low} signature showed a better survival compared to those with a RMPA^{high} signature. Similar findings were not observed in the TCGA mRNA-seq and Tiantan data sets.

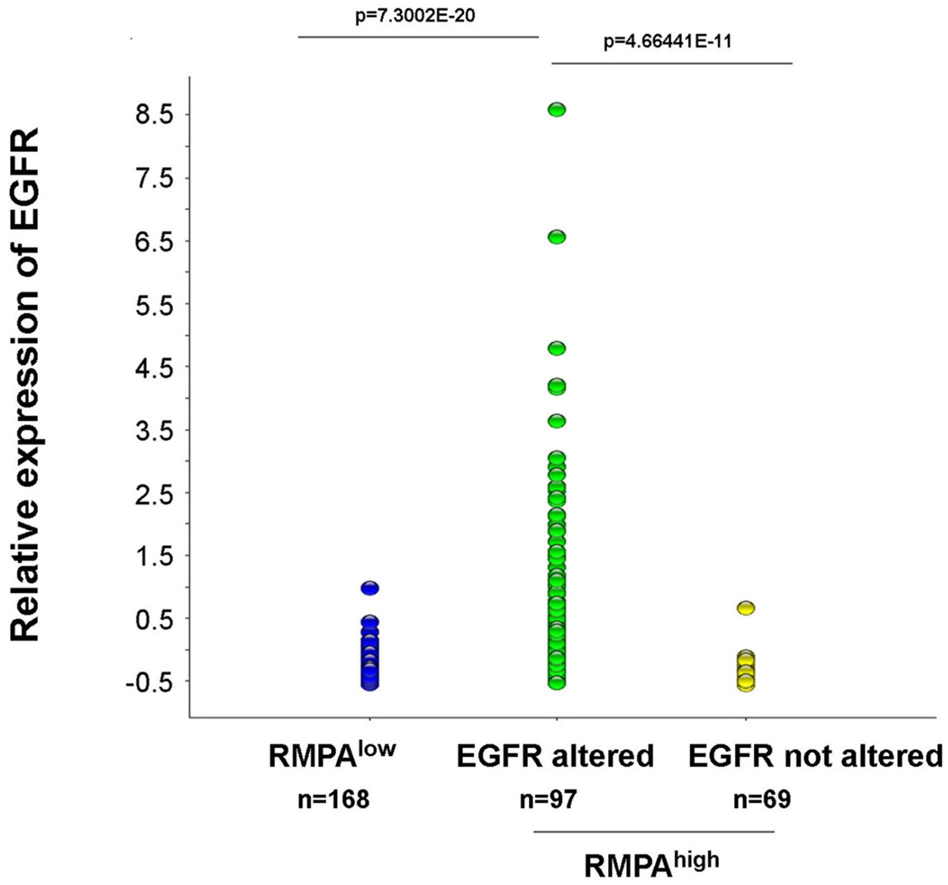

Supplementary Figure S4: Correlation between EGFR expression and *EGFR* genomic alterations in the RMPA^high gliomas. Relative expression levels of EGFR were analyzed in the TCGA mRNA-seq data set according to RMPA classification and the status of genomic alterations in *EGFR*. The mean expression levels of EGFR between the indicated subtypes were analyzed using *t* test.

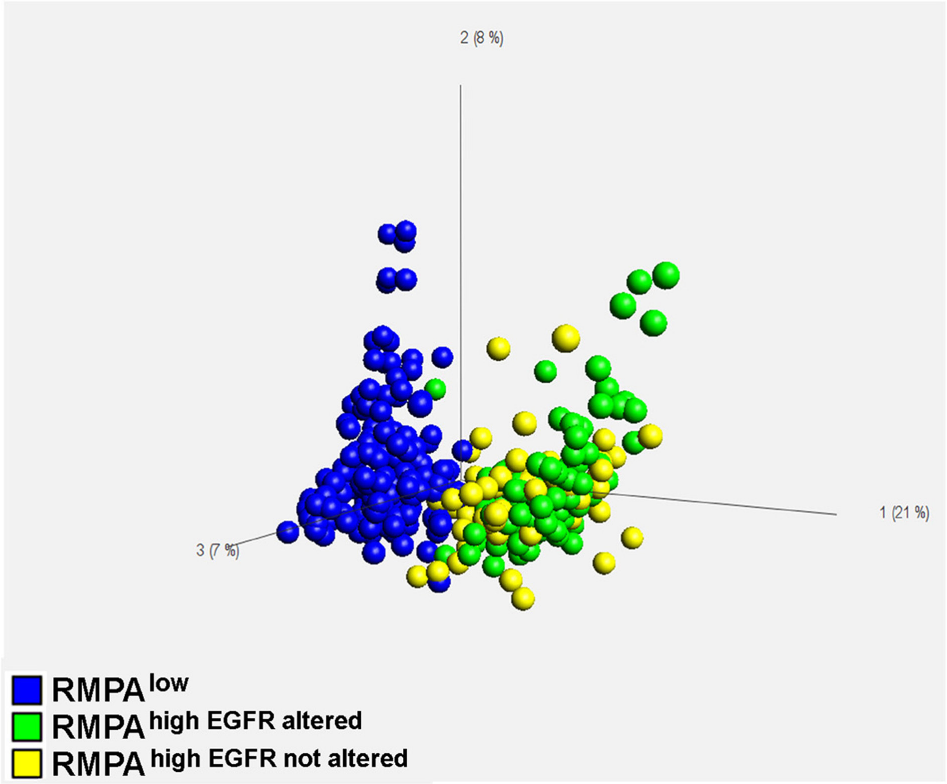

Supplementary Figure S5: Similar transcritome profiles between the RMPA^{high} gliomas with or without *EGFR* alterations in the TCGA mRNA-seq data set. Unsupervised principle component analysis of the whole transcriptome was performed. Under all conditions tested (as exemplified in the plot with 36% of the total variance including the expression of 12035 of 20502 genes), RMPA^{high} gliomas with or without *EGFR* alterations showed similar transcriptomic profiles.

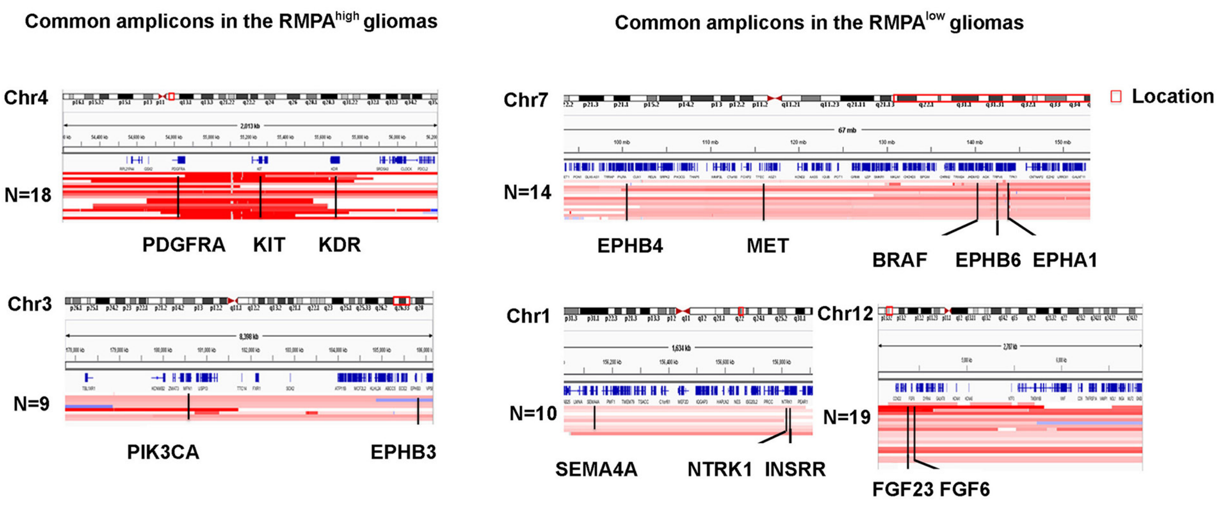

Supplementary Figure S6: Common amplicons in the RMPA^{high} and RMPA^{low} gliomas. The glad files for the TCGA mRNA-seq data set samples with the indicated SCNAs were analyzed using IGV. Chromosomal regions harboring common amplicons are depicted.

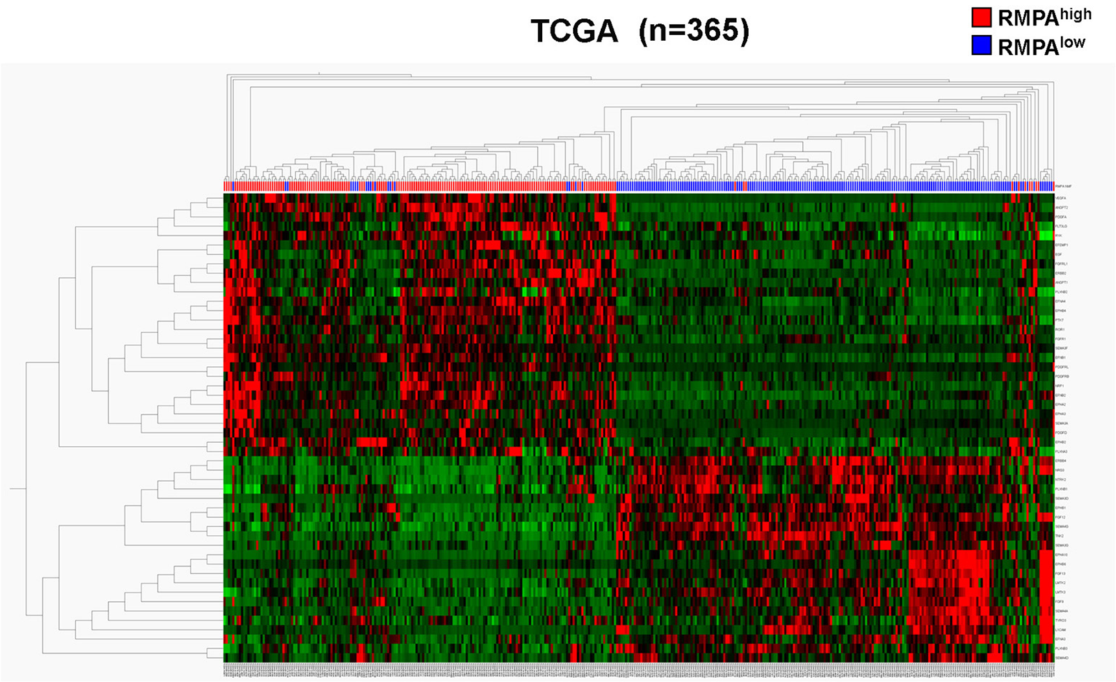

EGF, AREG
VEGFA/NRP1, NRP2
ANGPT1, ANGPT2
EFNA4/EPHA2, EPHA3
EFNB1, EFNB2/EPHB2, EPHB4
PDGFD/PDGFRB
SEMA3A/PLXNA3, NRP1
ERBB2
FGFR1
HGF/MET
DDR2
ROR1/RYK

NRG3/ERBB3, ERBB4
NTRK2, NTRK3
FGF9, FGF12, FGF13, FGF14
EFNB3/EPHB6, EPHA10
SEMA4D/PLXNB1, PLXNB3

RTKs and ligands with concordant expression pattern in ≥ 4 data sets (p=1e-6, q=2e-6 ~ 3.9e-7).

KDR
CSF1
VEGFR1

FGFR2
PDGFRA

Additional RTKs and ligands at p=1e-3, q=1e-3, concordant in ≥ 3 data sets.

Supplementary Figure S7: Enrichment of angiogenesis-related RTKs and their ligands in the RMPA^{high} gliomas. The expression pattern of RTKs and their ligands in the TCGA mRNA-seq data set was compared between the RMPA^{high} and RMPA^{low} gliomas using hierarchical clustering at a p value of $1x10^{-6}$ and q value of $2x10^{-6}$, or at p and q values at the range of $1x10^{-3}$. Enrichment of angiogenesis-related RTKs and their ligands in the RMPA^{high} gliomas was seen. Similar data were obtained from the CGGA mRNA-seq, Tiantan, GSE16011, Rembrandt data sets. Genes enriched to a similar extent in at least four of the five data sets are summarized in Supplementary Table S9.

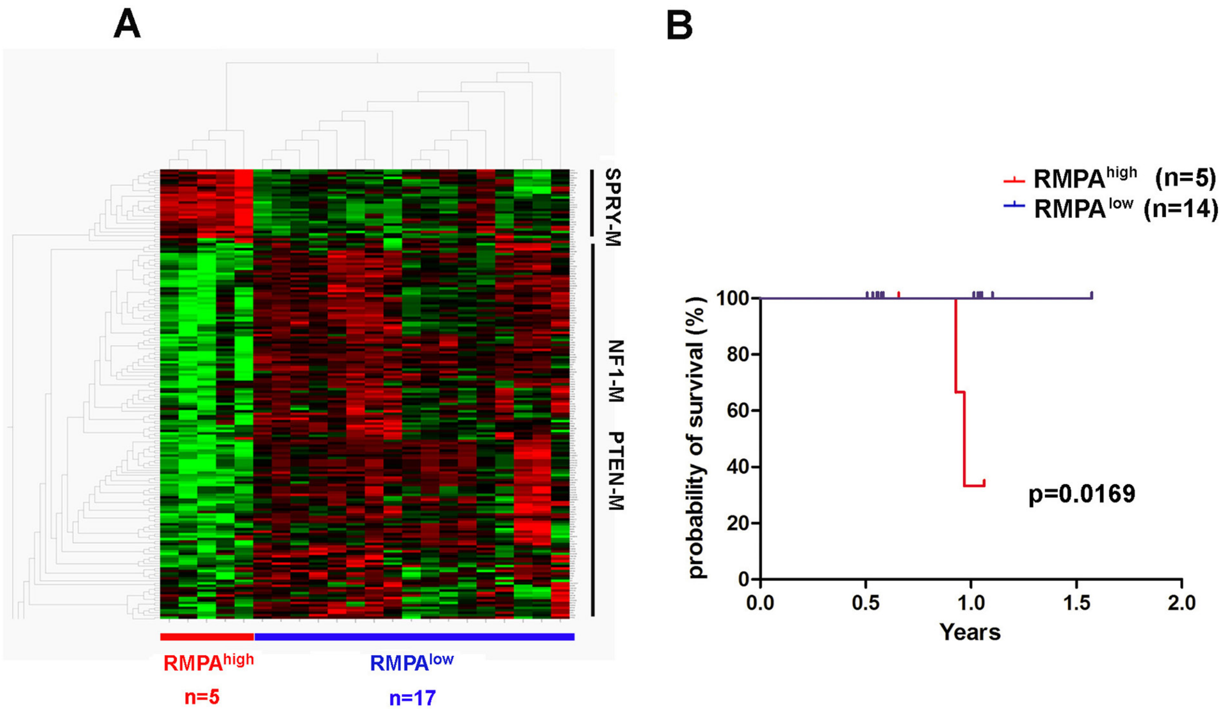

Supplementary Figure S8: Identification of RMPA^{high} and RMPA^{low} gliomas in twenty-two freshly isolated gliomas. Unsupervised hierarchical clustering of the expression data for the SPRY-M, NF1-M and PTEN-M was performed on the transcriptome data of 22 fresh gliomas. Five GBMs with RMPA^{high} and 17 gliomas (2 AII, 1 O II, 7 OA II, 4 OA III and 3 GBM) with RMPA^{low} signatures were identified. Patients with GBMs of RMPA^{high} signature showed poor survival compared to patients with gliomas of RMPA^{low} signature.

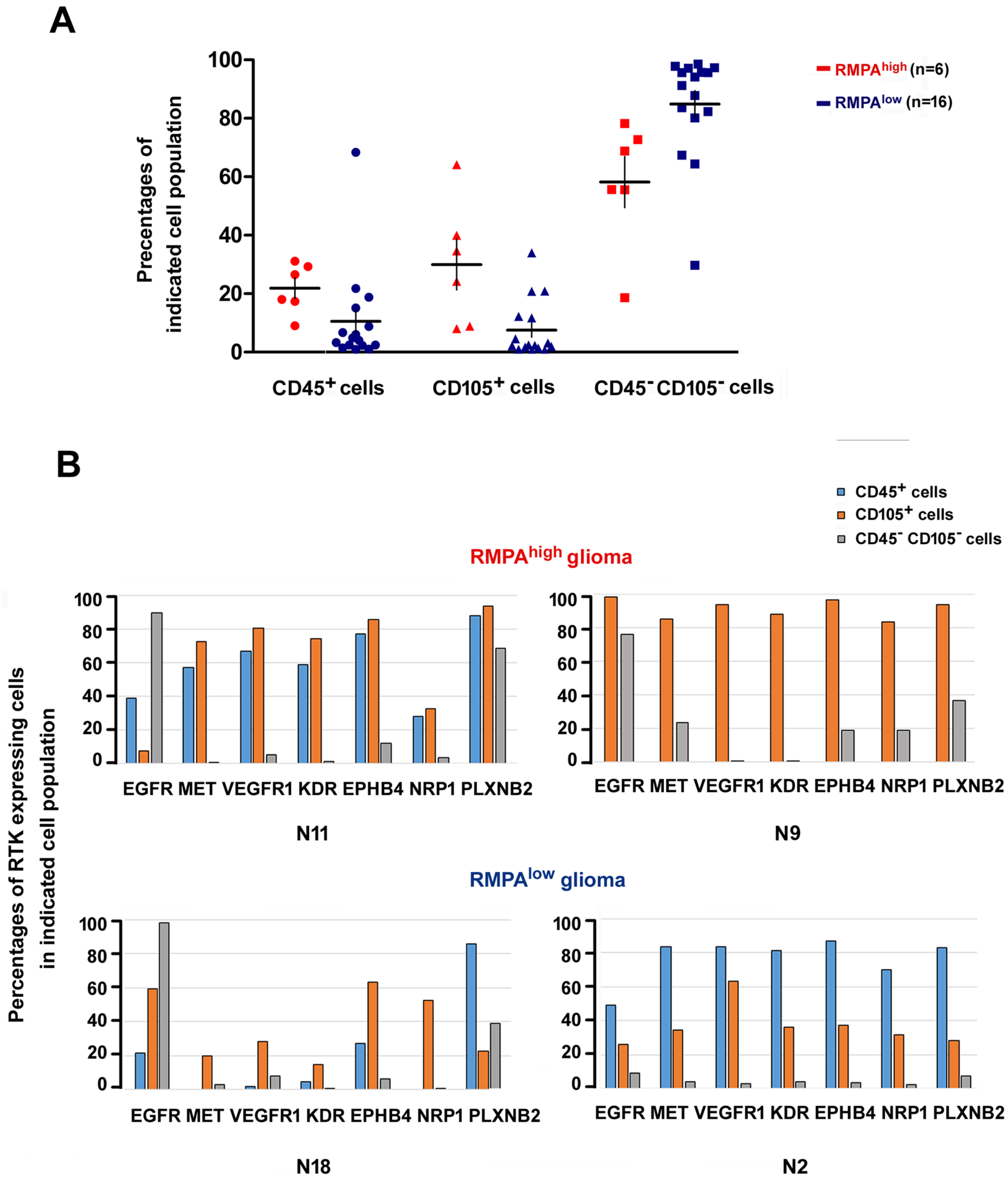

Supplementary Figure S9: Enriched expression of angiogenic RTKs in vessel endothelial cells and infiltrating immune cells in RMPA^{high} gliomas. Single cells from RMPA^{high} or RMPA^{low} gliomas were co-stained with APC-conjugated anti-CD45 or anti-CD105 mAbs in combination with one of the PE-conjugated anti-RTK mAbs, or with isotype-matched control antibodies. Living cells negatively stained with 7-AAD were analyzed for the co-expression between CD45, CD105 and RTKs as depicted in Figure 4. The percentages of CD45^{+} cells, CD105^{+} cells and CD45^{-}CD105^{-} cells in individual RMPA^{high} or RMPA^{low} gliomas are summarized **A.**, the bars indicate the median percentages of the indicated cell population in all 6 RMPA^{high} or 16 RMPA^{low} gliomas. Panel **B.** shows RTK expression within indicated cell populations of the representative RMPA^{high} or RMPA^{low} gliomas. RTK expression in the indicated cell populations calculated as a fraction of all living cells is summarized in Supplementary Table S11.

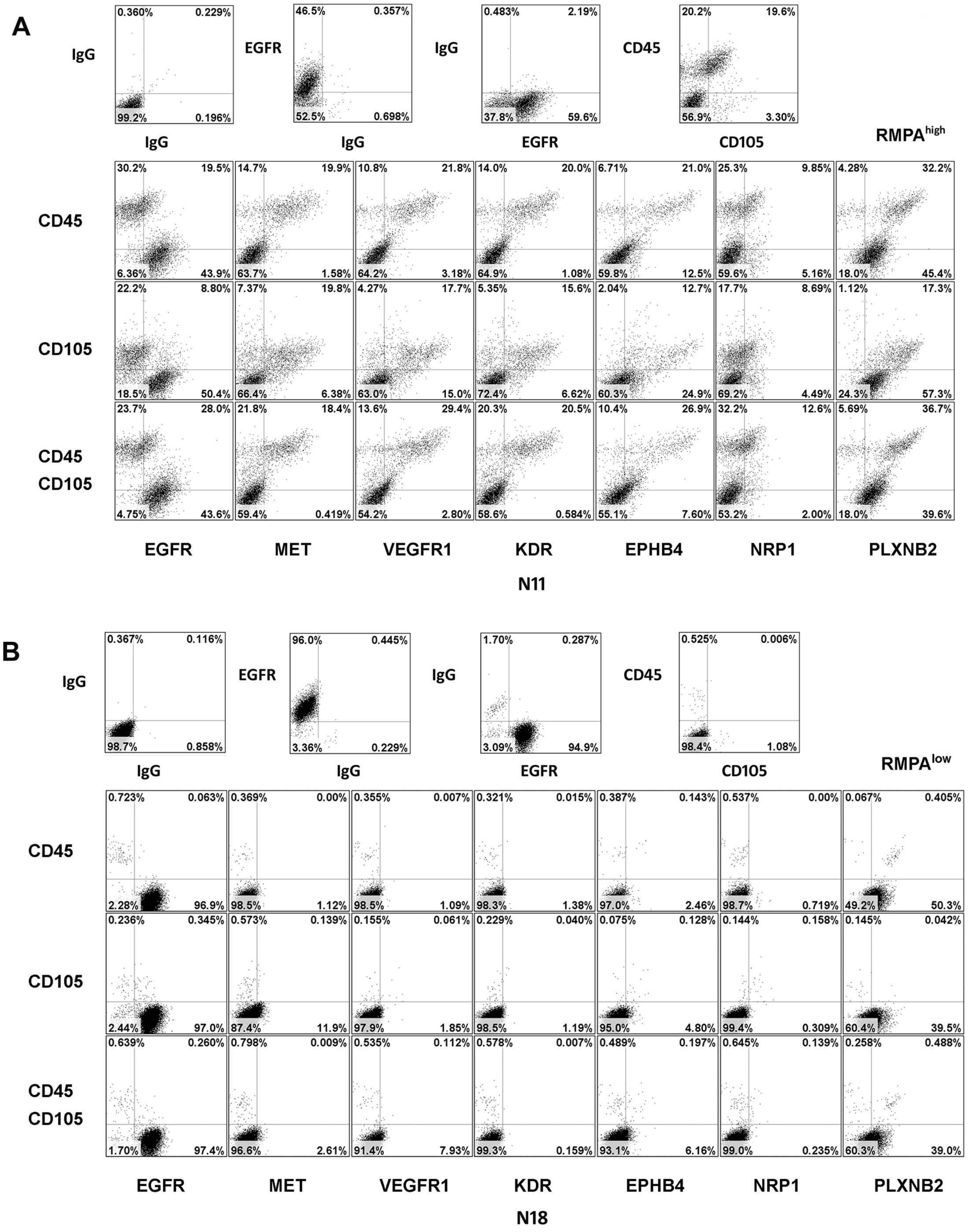

Supplementary Figure S10: Co-staining strategies for detection of RTK expression in cell subpopulations of RMPA^{high} and RMPA^{low} gliomas. Single cells from RMPA^{high} or RMPA^{low} gliomas were co-stained with APC-conjugated anti-CD45 or anti-CD105 mAbs, in combination with one of the PE-conjugated anti-RTK mAbs, or with isotype-matched control mAbs. Dot plots of the bottom rows were the results of co-staining of APC-conjugated anti-CD45 and anti-CD105 mAbs together with one of the indicated PE-conjugated anti-RTK mAb. Living cells excluding 7-AAD staining were gated and analyzed for the co-expression of CD45, CD105 and RTKs. Isotype control staining and the control stainings shown in the upper panels were used to set up the parameters for flow cytometry measurement, and also for the basal line intensities in the flow-jo analysis. In all dot-plots, the x axis indicates staining results of PE-conjugated mAbs, and y axis APC-conjugated mAbs.

RMPA^{high} (Sample 495)

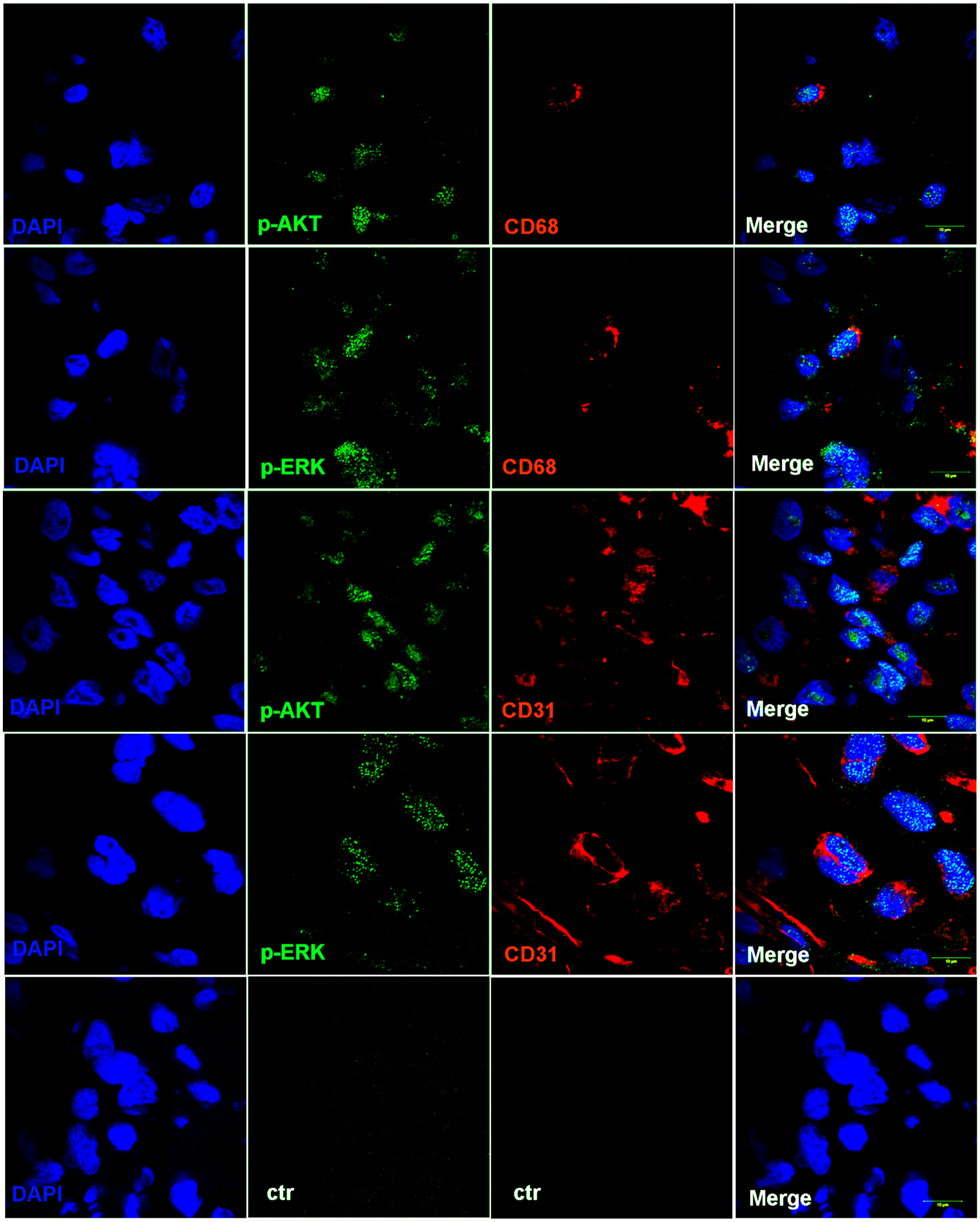

(*Continued*)

Grade IV (Sample 117117)

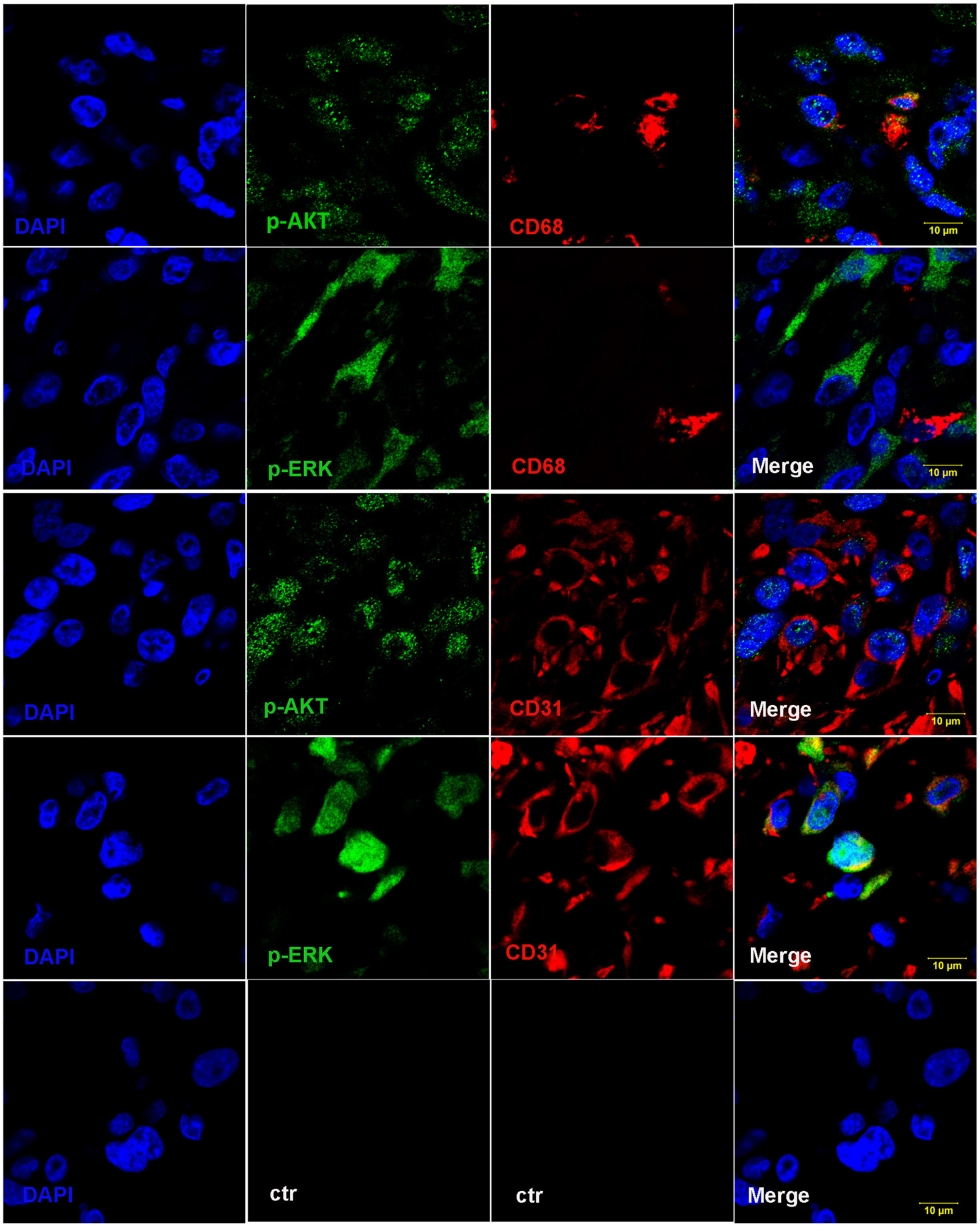

(*Continued*)

Grade IV (Sample 117055)

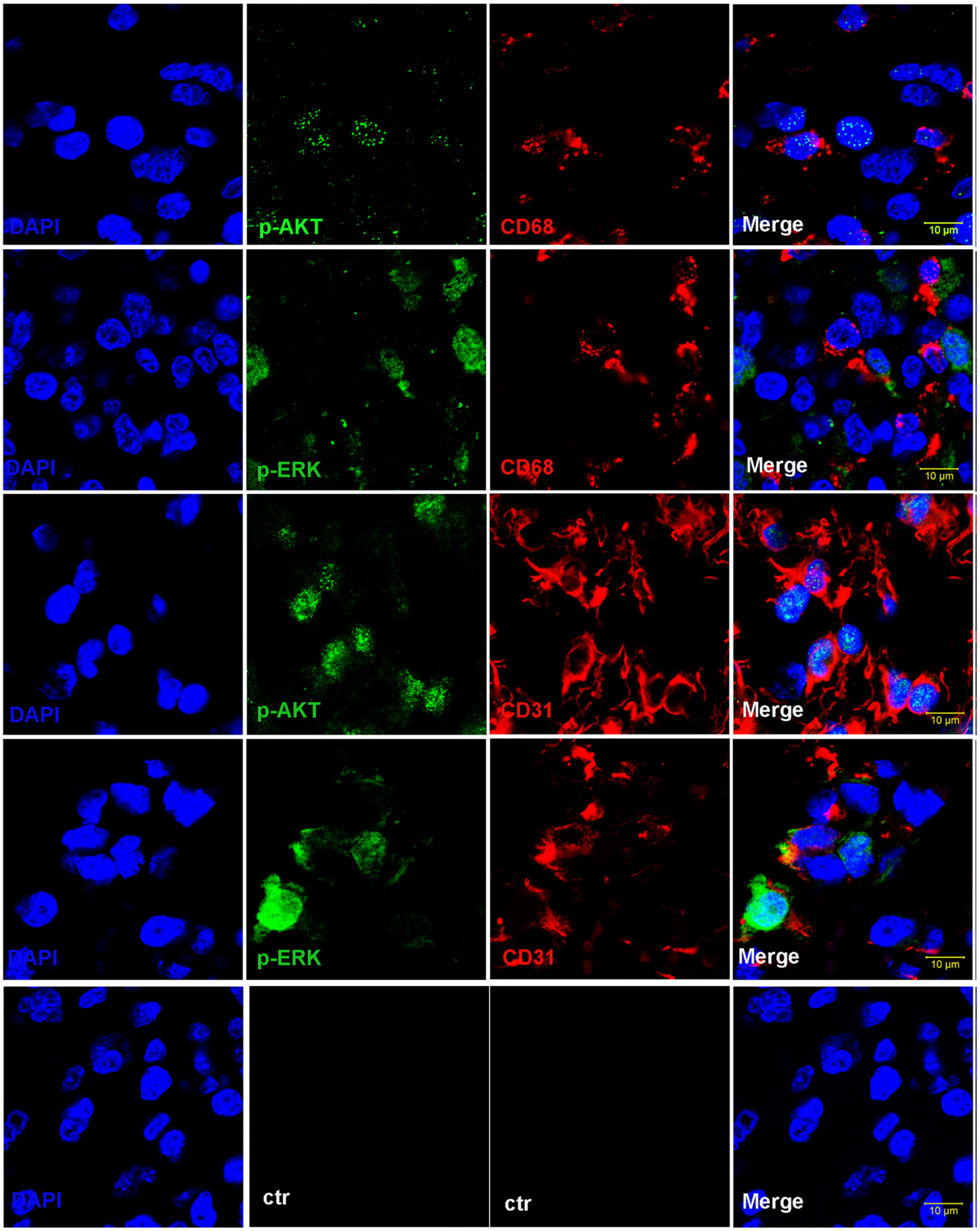

(*Continued*)

RMPA^{low} (Sample 598)

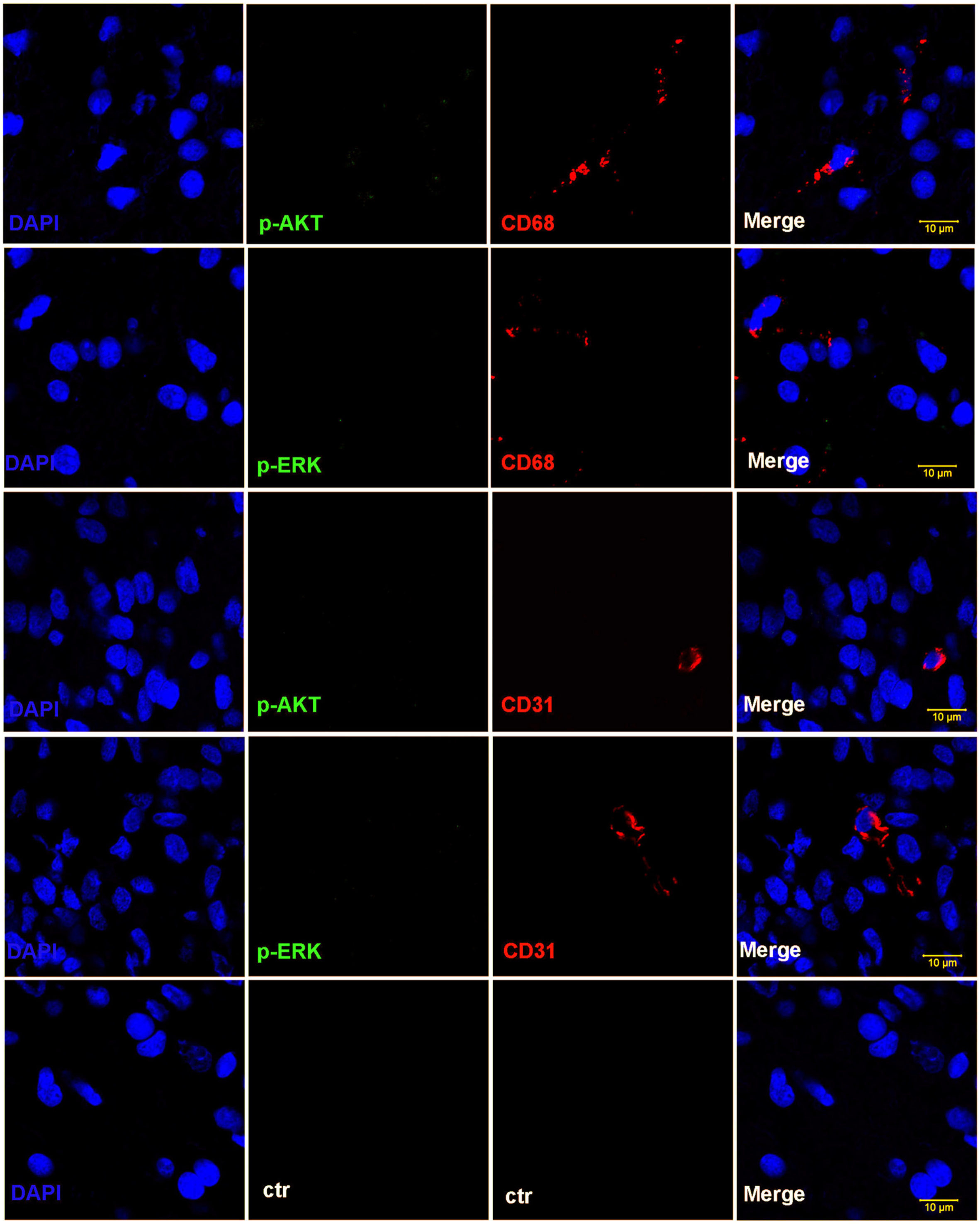

(Continued)

RMPA^{low} (Sample 1116)

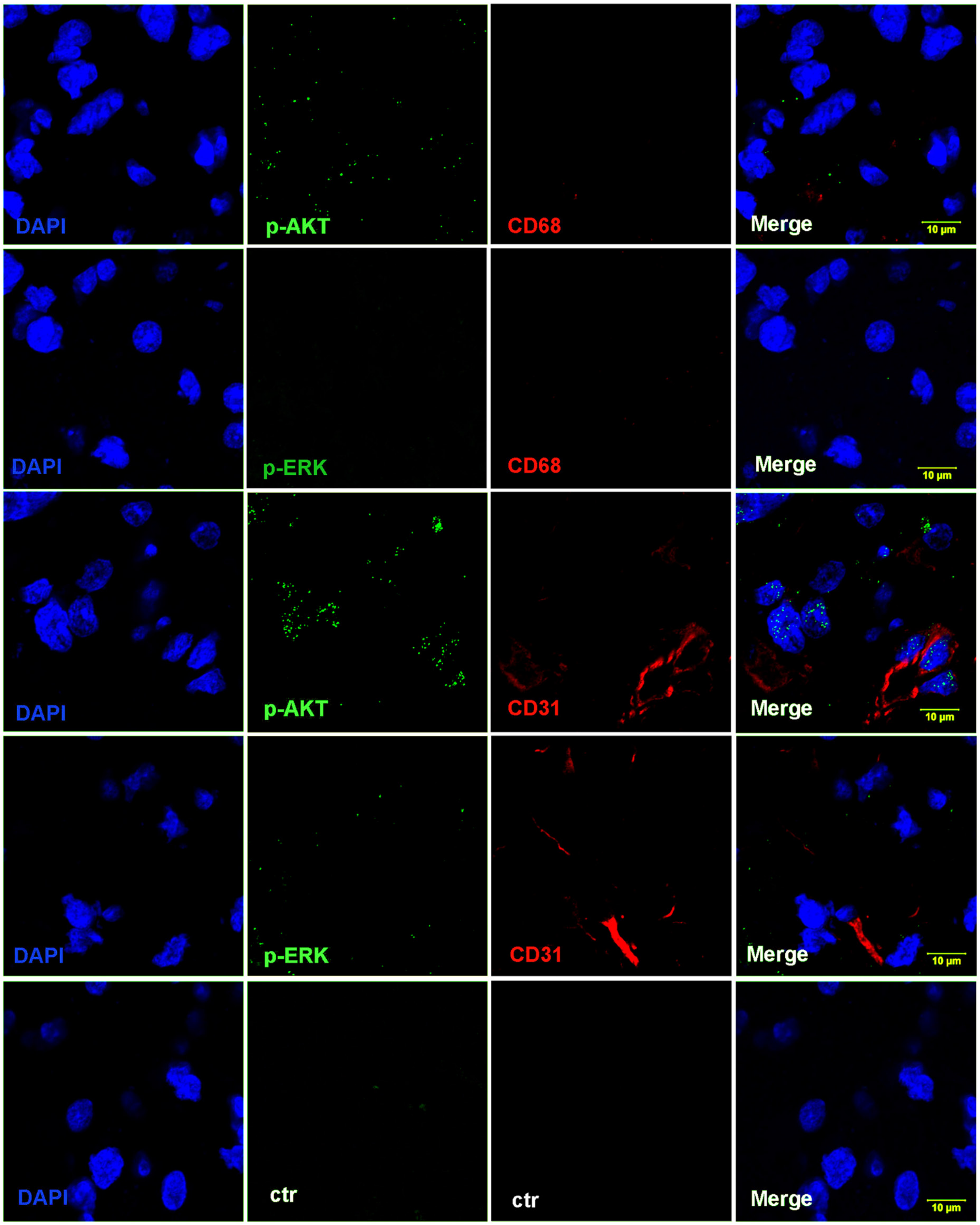

(*Continued*)

Grade II (Sample 116896)

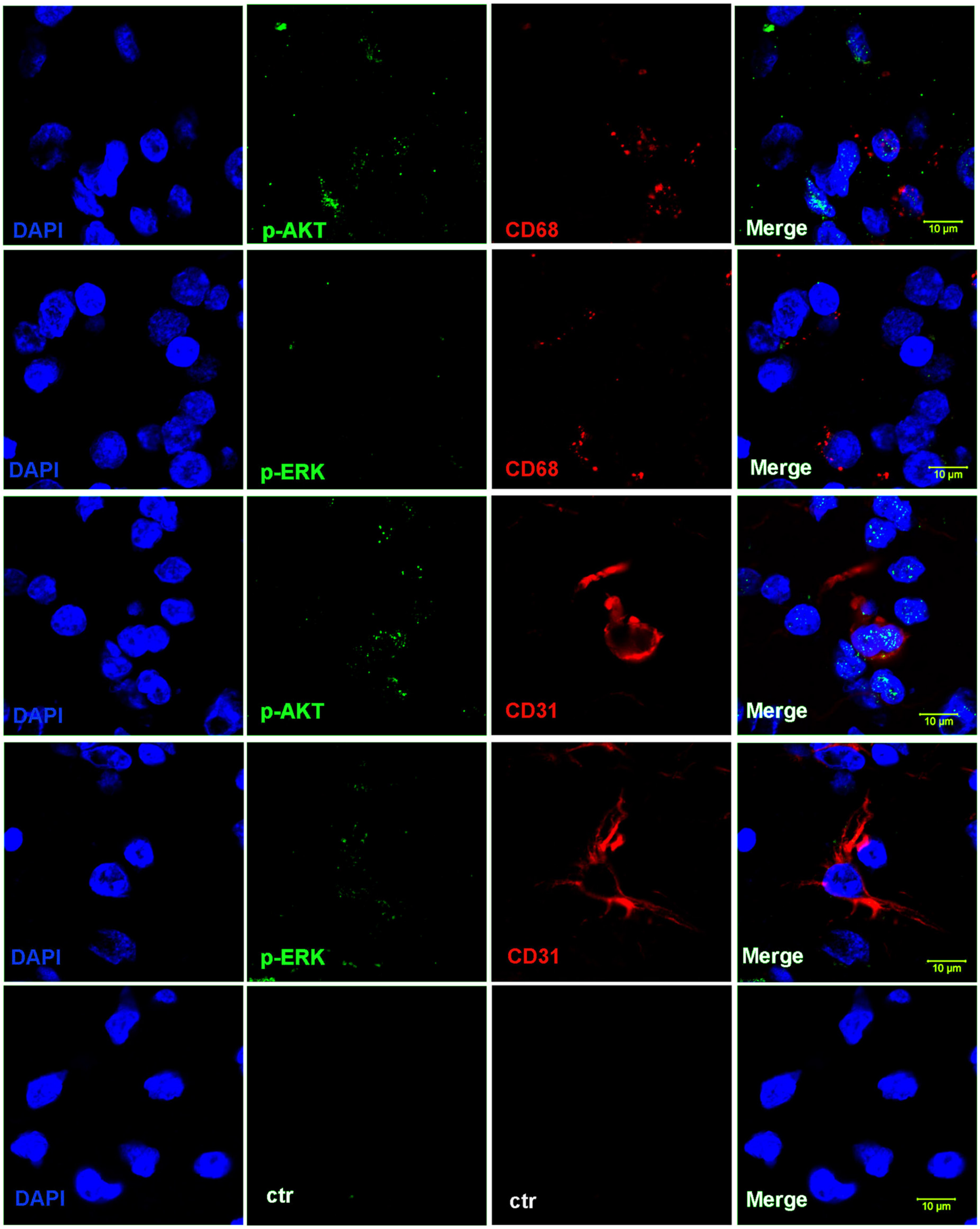

Supplementary Figure S11: Activation of RTK signaling in vessel endothelial cells and TAMs in RMPA^{high} gliomas. Sections of glioma samples were co-stained with mAbs against CD31 or CD68 in combination with mAbs towards p-AKT or p-ERK. The staining of anti-CD31 or anti-CD68 was detected with Alexa Fluor® 555 conjugated secondary antibody; and anti-p-AKT or anti-p-ERK with Alexa Fluor® 647 conjugated secondary antibody. Sections were further staininned with DAPI and evaluated using a confocal microscope (ZEISS LSM 700). Images of RMPA classified gliomas, or morphologically defined high-grade or low-grade gliomas are shown. Strong staining of p-AKT or p-ERK was detected in CD31-positive endothelial cells and CD68-positive TAM cells.

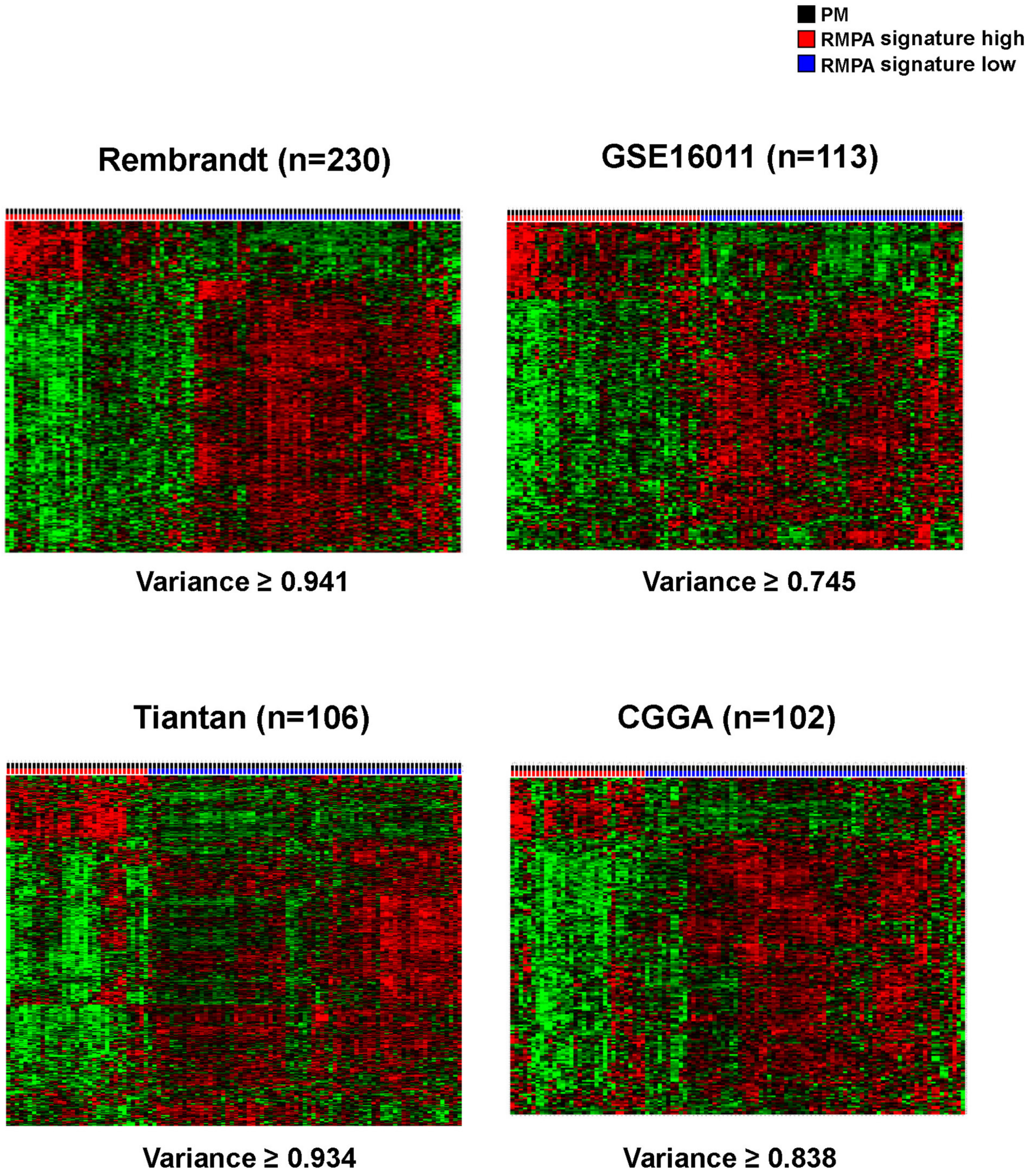

Supplementary Figure S12: Clustering of PM gliomas with RMPA signature. Unsupervised hierarchical clustering between the expression data of NF1-M, PTEN-M and SRY-M and the PM gliomas from the REMBRANDT, GSE16011, Tiantan and CGGA were performed. Genes with their standard deviation below 0.059, 0.255, 0.066, 0.162 in the REMBRANDT, GSE16011, Tiantan and CGGA data sets, respectively, were excluded. Heatmaps show that PM gliomas from all these data sets were separated into two subgroups with relative high or low RMPA signature.

Supplementary Table S1: Frequent losses of members of NF1-M, PTEN-M in gliomas with RMPA^{high} phenotype

See Supplementary File 1

Supplementary Table S2: Glioma data sets used in this study

Data set	Platform	Composition	Country of Origin
1) Tiantan[1]	Agilent44K array	209gliomas, WHO grades II-IV	China
2) CGGA (GSE48865[2])	IlluminaHiSeq_RNASeq	210gliomas, WHO grades II-IV	China
3) GSE16011[1,3]	HG-U133 Plus 2.0	247gliomas, WHO grades II-IV; 8normal controls	Holland
4) REMBRANDT[1,4]	HG-U133 Plus 2.0	521gliomas, WHO grades II-IV; 21 epilepsy	USA
5) TCGA mRNA-seq[1]	IlluminaHiSeq_RNASeq	365gliomas, WHO grades II-IV	USA

*1. Sun, Y. et al. A glioma classification scheme based on coexpression modules of EGFR and PDGFRA. PNAS**111**, 3538-43 (2014).*
*2. Bao, Z.S. et al. RNA-seq of 272 gliomas revealed a novel, recurrent PTPRZ1-MET fusion transcript in secondary glioblastomas. Genome Res**24**, 1765-73 (2014).*
*3. Gravendeel, L.A. et al. Intrinsic gene expression profiles of gliomas are a better predictor of survival than histology. Cancer Res**69**, 9065-72 (2009).*
4. Madhavan, S. et al. Rembrandt: helping personalized medicine become a reality through integrative translational research. Mol Cancer Res7, 157-67 (2009).

Supplementary Table S3: Comparison of NF1-M, PTEN-M and SPRY-M expression between non-tumor and RMPA^{low} glioma in GSE16011 and Rembrandt datasets

Average expression	Rembrandt			GSE16011		
	RMPA^{low} gliomas n=281	Non-tumor gliomas n=21	t-test	RMPA^{low} gliomas n=110	Non-tumor gliomas n=8	t-test
**NF1-M**	901.7 ± 204.6	917.2 ± 111.9	P=0.730936	780.8 ± 168.3	736.7 ± 51.5	P=0.464108
**PTEN-M**	726.7 ± 201.6	1205.6 ±177.2	P=1.89516E-22	637.1 ± 148.8	1250.8 ± 178.7	P=5.50698E-20
**SPRY-M**	495.8 ± 146.6	418.6 ±50.7	P=0.01702	433.2 ± 163.3	266.0 ± 21.8	P=0.004704

Probe sets of the NF1-M, PTEN-M and SPRY-M with a differential expression level between epileptic brain tissues and RMPA^{low} gliomas at a p value < 0.001 were identified in the GSE16011 and REMBRANDT data sets. The average expression levels of these probe sets in the epileptic brain tissues or RMPA^{low} gliomas were calculated and subsequently compared using t test.

Supplementary Table S4: Regional chromosomal alteration in RMPA^{high} and RMPA^{low} gliomas from the TCGA mRNA-Seq data set

See Supplementary File 1

Supplementary Table S5: Regional chromosomal alteration in RMPA^{high} and RMPA^{low} gliomas from Rembrandts data set

See Supplementary File 1

Supplementary Table S6: RMPA^{high} and RMPA^{low} glioma-specific focal SCNAs of RTK signaling related genes in the TCGA mRNA-Seq data set

See Supplementary File 1

Supplementary Table S7: Odds ratio of alterations in RTK signaling pathway from RMPA^{high} gliomas

See Supplementary File 1

Supplementary Table S8: Odds ratio of alterations in RTK signaling pathway from RMPA^{low} gliomas

See Supplementary File 1

Supplementary Table S9: Differentially expressed RTKs and ligands between RMPA^{high} and RMPA^{low} gliomas in at least four glioma datasets

See Supplementary File 1

Supplementary Table S10: Percentages of CD45^{+}, CD105^{+} and CD45^{-}CD105^{-} cell populations in RMPA^{high} and RMPA^{low} gliomas

See Supplementary File 1

Supplementary Table S11: Cell population-based analysis of RTK expressing cells as a fraction of all living cells in glioma

See Supplementary File 1
