# Supplementary material for: Co-expression modules of NF1, PTEN and sprouty enable distinction of adult diffuse gliomas according to pathway activities of receptor tyrosine kinases
Source: Oncotarget. 2016 Jul 1;7(37):59098–114. doi: 10.18632/oncotarget.10359 (PMC5312298; doi:10.18632/oncotarget.10359)
Supplement: Supplementary file 2 [file oncotarget-07-59098-s002.docx]

|  |  |  |  |  |  | Page 1 |  |  |  |  |  | NF1-M | Table S1 |
| --- | --- | --- | --- | --- | --- | --- | --- | --- | --- | --- | --- | --- | --- |
| **Table S1. Frequent losses of members of NF1-M, PTEN-M in gliomas with RMPA^high^ phenotype.** | | | | | | | | | | | | | |
| **NF1-M (n=85)** | **Cytoband** | **RMPA^high^ (n=166)** | | | | | | **RMPA^low^(n=168)** | | | | | |
|  |  | **SCNA(alldata)** | | **Genedosage effect** | | **Modulating effect** | | **SCNA(alldata)** | | **Genedosage effect** | | **Modulating effect** | |
|  |  | **del** | **amp** | **R** | **P** | **β** | **P** | **del** | **amp** | **R** | **P** | **β** | **P** |
| ZRANB1 | 10q26.13 | 149 | 0 | 0.369 | p<0.0001 | 0.499 | p<0.0001 | 27 | 0 | 0.401 | p<0.0001 | 0.341 | p<0.0001 |
| HIF1AN | 10q24.31 | 148 | 0 | 0.521 | p<0.0001 | 0.31 | p<0.0001 | 18 | 0 | 0.312 | p<0.0001 | 0.283 | p<0.0001 |
| CPEB3 | 10q23.32 | 147 | 0 | 0.422 | p<0.0001 | 0.253 | p<0.0001 | 17 | 0 | 0.32 | p<0.0001 | 0.131 | p<0.0001 |
| ANKRD46 | 8q22.2 | 146 | 0 | No effect |  |  |  | 17 | 0 | 0.221 | 0.004 | 0.206 | p<0.0001 |
| RPP30 | 10q23.31 | 146 | 0 | 0.261 | 0.001 |  |  | 17 | 0 | No effect |  |  |  |
| ARL3 | 10q24.32 | 146 | 0 | 0.227 | 0.003 |  |  | 17 | 1 | 0.187 | 0.015 | 0.155 | 0.002 |
| TRIM8 | 10q24.32 | 146 | 0 | 0.503 | p<0.0001 | 0.271 | p<0.0001 | 17 | 1 | 0.243 | 0.002 |  |  |
| GRID1 | 10q23.1 | 146 | 0 | 0.254 | 0.001 | 0.142 | p<0.0001 | 17 | 0 | 0.49 | p<0.0001 | 0.249 | p<0.0001 |
| FAM190B | 10q23.1 | 146 | 0 | 0.466 | p<0.0001 | 0.445 | p<0.0001 | 16 | 0 | 0.264 | 0.001 | 0.405 | p<0.0001 |
| PHYHIPL | 10q21.1 | 145 | 0 | 0.225 | 0.004 | 0.211 | p<0.0001 | 10 | 0 | No effect |  |  |  |
| MAPK8 | 10q11.22 | 144 | 0 | 0.275 | p<0.0001 | 0.3 | p<0.0001 | 7 | 0 | No effect |  |  |  |
| ZNF248 | 10p11.1 | 143 | 3 | 0.301 | p<0.0001 | 0.296 | p<0.0001 | 5 | 10 | 0.166 | 0.031 | 0.156 | p<0.0001 |
| WAC | 10p12.1 | 143 | 2 | 0.56 | p<0.0001 | 0.52 | p<0.0001 | 5 | 22 | 0.441 | p<0.0001 | 0.442 | p<0.0001 |
| RAB18 | 10p12.1 | 143 | 2 | 0.571 | p<0.0001 | 0.126 | 0.041 | 5 | 22 | 0.433 | p<0.0001 | 0.234 | p<0.0001 |
| UPF2 | 10p14 | 141 | 2 | 0.38 | p<0.0001 | 0.287 | p<0.0001 | 5 | 25 | 0.372 | p<0.0001 | 0.259 | p<0.0001 |
| ZMYND11 | 10p15.3 | 141 | 3 | 0.395 | p<0.0001 | 0.502 | p<0.0001 | 7 | 28 | 0.546 | p<0.0001 | 0.309 | p<0.0001 |
| FRY | 13q13.1 | 56 | 4 | 0.197 | 0.011 | 0.305 | p<0.0001 | 29 | 2 | 0.312 | p<0.0001 | 0.268 | p<0.0001 |
| AKAP6 | 14q12 | 54 | 3 | 0.276 | p<0.0001 | 0.164 | p<0.0001 | 7 | 4 | No effect |  |  |  |
| PNMA1 | 14q24.3 | 52 | 4 | 0.599 | p<0.0001 | 0.158 | 0.001 | 27 | 0 | 0.379 | p<0.0001 | 0.238 | p<0.0001 |
| BCR | 22q11.23 | 52 | 6 | 0.222 | 0.004 | 0.187 | p<0.0001 | 8 | 2 | 0.203 | 0.008 | 0.141 | p<0.0001 |
| GPHN | 14q23.3 | 51 | 5 | 0.573 | p<0.0001 | 0.115 | 0.008 | 27 | 0 | 0.411 | p<0.0001 | 0.184 | p<0.0001 |
| TUB | 11p15.4 | 50 | 3 | No effect |  |  |  | 27 | 0 | 0.269 | p<0.0001 | 0.2 | p<0.0001 |
|  |  |  |  |  |  | Page 2 |  |  |  |  |  | NF1-M | Table S1 |
| DCAF5 | 14q24.1 | 50 | 6 | 0.653 | p<0.0001 | 0.15 | 0.02 | 27 | 0 | 0.372 | p<0.0001 | 0.356 | p<0.0001 |
| PPP2R5C | 14q32.31 | 49 | 4 | 0.671 | p<0.0001 |  |  | 19 | 2 | No effect |  |  |  |
| KPNA5 | 6q22.1 | 48 | 3 | 0.493 | p<0.0001 | 0.203 | p<0.0001 | 18 | 1 | 0.187 | 0.015 | 0.201 | p<0.0001 |
| RNF146 | 6q22.33 | 45 | 3 | 0.633 | p<0.0001 | 0.281 | p<0.0001 | 17 | 1 | No effect |  |  |  |
| SPIRE1 | 18p11.21 | 41 | 3 | No effect |  |  |  | 23 | 3 | 0.279 | p<0.0001 | 0.254 | p<0.0001 |
| GTF2H5 | 6q25.3 | 41 | 4 | 0.414 | p<0.0001 |  |  | 20 | 0 | 0.301 | p<0.0001 | -0.113 | 0.018 |
| WDFY3 | 4q21.23 | 26 | 4 | No effect |  |  |  | 30 | 9 | 0.577 | p<0.0001 | 0.311 | p<0.0001 |
| TERF2 | 16q22.1 | 25 | 4 | 0.462 | p<0.0001 | 0.471 | p<0.0001 | 5 | 2 | 0.41 | p<0.0001 | 0.426 | p<0.0001 |
| FAM192A | 16q13 | 24 | 3 | 0.573 | p<0.0001 | 0.391 | p<0.0001 | 4 | 2 | 0.302 | p<0.0001 | 0.382 | p<0.0001 |
| TMEM170B | 6p24.2 | 23 | 4 | 0.28 | p<0.0001 | 0.416 | p<0.0001 | 8 | 4 | No effect |  |  |  |
| PPP6C | 9q33.3 | 19 | 15 | 0.53 | p<0.0001 | 0.181 | 0.03 | 8 | 7 | No effect |  |  |  |
| NTRK2 | 9q21.33 | 19 | 16 | 0.262 | 0.001 | 0.129 | p<0.0001 | 6 | 3 | -0.199 | 0.01 | 0.058 | 0.003 |
| ALDH5A1 | 6p22.3 | 19 | 5 | 0.239 | 0.002 | 0.23 | p<0.0001 | 6 | 3 | 0.156 | 0.043 | 0.223 | p<0.0001 |
| RFTN2 | 2q33.1 | 18 | 3 | No effect |  |  |  | 3 | 2 | No effect |  |  |  |
| HDAC4 | 2q37.3 | 18 | 3 | No effect |  |  |  | 3 | 2 | No effect |  |  |  |
| SPTAN1 | 9q34.11 | 18 | 18 | 0.25 | 0.001 | 0.317 | p<0.0001 | 8 | 7 | No effect |  |  |  |
| NF1 | 17q11.2 | 18 | 7 | 0.287 | p<0.0001 | 0.301 | p<0.0001 | 2 | 2 | 0.28 | p<0.0001 | 0.434 | p<0.0001 |
| THRA | 17q21.1 | 17 | 6 | 0.3 | p<0.0001 | 0.232 | p<0.0001 | 1 | 2 | No effect |  |  |  |
| WIPF2 | 17q21.1 | 17 | 5 | 0.195 | 0.012 | 0.317 | p<0.0001 | 1 | 2 | -0.269 | p<0.0001 | 0.151 | 0.001 |
| FBXL20 | 17q12 | 17 | 5 | 0.338 | p<0.0001 | 0.226 | p<0.0001 | 0 | 2 | 0.169 | 0.028 | 0.231 | p<0.0001 |
| CALCOCO1 | 12q13.13 | 16 | 11 | 0.179 | 0.021 | 0.483 | p<0.0001 | 17 | 2 | 0.289 | p<0.0001 | 0.249 | p<0.0001 |
| CDH10 | 5p14.2 | 15 | 0 | No effect |  |  |  | 27 | 1 | 0.159 | 0.04 | 0.216 | p<0.0001 |
| DNAJC18 | 5q31.2 | 15 | 10 | 0.16 | 0.04 | 0.358 | p<0.0001 | 9 | 0 | No effect |  |  |  |
| KDM3B | 5q31.2 | 15 | 10 | 0.155 | 0.046 | 0.532 | p<0.0001 | 9 | 1 | No effect |  |  |  |
| C5orf41 | 5q35.1 | 15 | 8 | 0.175 | 0.024 | 0.237 | p<0.0001 | 11 | 2 | 0.303 | p<0.0001 | 0.221 | p<0.0001 |
| KCNIP3 | 2q11.1 | 14 | 0 | No effect |  |  |  | 28 | 1 | No effect |  |  |  |
|  |  |  |  |  |  | Page 3 |  |  |  |  |  | NF1-M | Table S1 |
| GLUD1 | 10q23.2 | 14 | 0 | No effect |  |  |  | 27 | 0 | No effect |  |  |  |
| ADAM22 | 7q21.12 | 14 | 0 | No effect |  |  |  | 27 | 1 | 0.231 | 0.003 | 0.178 | p<0.0001 |
| AP2B1 | 17q12 | 14 | 7 | 0.338 | p<0.0001 | 0.66 | p<0.0001 | 2 | 2 | No effect |  |  |  |
| PCM1 | 8p22 | 14 | 11 | 0.198 | 0.011 | 0.26 | p<0.0001 | 4 | 12 | 0.218 | 0.005 | 0.314 | p<0.0001 |
| RPS15A | 16p12.3 | 13 | 10 | No effect |  |  |  | 15 | 3 | No effect |  |  |  |
| BCL7A | 12q24.31 | 13 | 7 | No effect |  |  |  | 4 | 2 | 0.202 | 0.009 | 0.18 | p<0.0001 |
| CXXC4 | 4q24 | 12 | 7 | No effect |  |  |  | 9 | 1 | No effect |  |  |  |
| WDR48 | 3p22.2 | 12 | 13 | 0.346 | p<0.0001 | 0.603 | p<0.0001 | 9 | 3 | No effect |  |  |  |
| SATB1 | 3p24.3 | 12 | 14 | 0.18 | 0.02 | 0.262 | p<0.0001 | 6 | 7 | No effect |  |  |  |
| ZNF148 | 3q21.2 | 12 | 17 | 0.208 | 0.007 | 0.255 | 0.001 | 12 | 1 | 0.152 | 0.049 | 0.484 | p<0.0001 |
| TMCC1 | 3q22.1 | 12 | 16 | 0.24 | 0.002 | 0.39 | p<0.0001 | 12 | 1 | 0.204 | 0.008 | 0.222 | p<0.0001 |
| MAPT | 17q21.31 | 12 | 7 | 0.243 | 0.002 | 0.212 | p<0.0001 | 2 | 3 | 0.217 | 0.005 | 0.271 | p<0.0001 |
| PID1 | 2q36.3 | 11 | 15 | No effect |  |  |  | 12 | 3 | No effect |  |  |  |
| METTL6 | 3p25.1 | 11 | 14 | 0.362 | p<0.0001 | -0.191 | 0.006 | 6 | 8 | No effect |  |  |  |
| NCOA2 | 8q13.3 | 11 | 11 | 0.221 | 0.004 | 0.201 | p<0.0001 | 2 | 16 | No effect |  |  |  |
| RAP2A | 13q32.1 | 10 | 10 | No effect |  |  |  | 6 | 0 | No effect |  |  |  |
| SLITRK5 | 13q31.2 | 10 | 4 | No effect |  |  |  | 25 | 1 | 0.317 | p<0.0001 | 0.143 | p<0.0001 |
| DENND5B | 12p11.21 | 10 | 16 | 0.156 | 0.045 | 0.289 | p<0.0001 | 1 | 9 | No effect |  |  |  |
| RPS20 | 8q12.1 | 10 | 12 | 0.166 | 0.032 | -0.212 | p<0.0001 | 1 | 16 | 0.183 | 0.017 |  |  |
| GDPD1 | 17q22 | 10 | 11 | 0.357 | p<0.0001 | 0.193 | p<0.0001 | 0 | 4 | 0.172 | 0.026 | 0.267 | p<0.0001 |
| RPL37 | 5p13.1 | 9 | 6 | No effect |  |  |  | 4 | 2 | No effect |  |  |  |
| CSMD3 | 8q23.3 | 9 | 13 | No effect |  |  |  | 1 | 24 | No effect |  |  |  |
| CEP68 | 2p14 | 9 | 6 | No effect |  |  |  | 4 | 1 | -0.153 | 0.048 | 0.461 | p<0.0001 |
| SLC1A4 | 2p14 | 9 | 11 | No effect |  |  |  | 16 | 6 | 0.394 | p<0.0001 |  |  |
| PUM2 | 2p24.1 | 9 | 8 | 0.234 | 0.002 | 0.5 | p<0.0001 | 7 | 3 | No effect |  |  |  |
| KIAA1429 | 8q22.1 | 9 | 21 | 0.319 | p<0.0001 | 0.288 | p<0.0001 | 1 | 27 | 0.249 | 0.001 | 0.213 | p<0.0001 |
|  |  |  |  |  |  | Page 4 |  |  |  |  |  | NF1-M | Table S1 |
| DCAF8 | 1q23.2 | 8 | 17 | No effect |  |  |  | 1 | 30 | No effect |  |  |  |
| PBX1 | 1q23.3 | 8 | 5 | No effect |  |  |  | 14 | 1 | No effect |  |  |  |
| BCAN | 1q23.1 | 7 | 5 | No effect |  |  |  | 10 | 1 | 0.201 | 0.009 | 0.07 | p<0.0001 |
| PPM1L | 3q25.33 | 6 | 5 | No effect |  |  |  | 6 | 1 | No effect |  |  |  |
| JMY | 5q14.1 | 6 | 6 | No effect |  |  |  | 1 | 1 | No effect |  |  |  |
| MBD5 | 2q23.1 | 6 | 7 | 0.164 | 0.035 | 0.389 | p<0.0001 | 4 | 1 | -0.159 | 0.039 | 0.336 | p<0.0001 |
| CUL3 | 2q36.2 | 1 | 24 | No effect |  |  |  | 0 | 10 | No effect |  |  |  |
| MAPK10 | 4q21.3 | 1 | 21 | No effect |  |  |  | 1 | 7 | No effect |  |  |  |
| GSTA4 | 6p12.2 | 1 | 23 | No effect |  |  |  | 0 | 8 | No effect |  |  |  |
| RPRD2 | 1q21.3 | 1 | 21 | 0.197 | 0.011 | 0.364 | p<0.0001 | 1 | 8 | 0.158 | 0.041 | 0.438 | p<0.0001 |
| CNOT6L | 4q21.1 | 0 | 131 | No effect |  |  |  | 0 | 28 | No effect |  |  |  |

|  |  |  |  |  |  | Page 1 |  |  |  |  |  | PTEN-M | Table S1 |
| --- | --- | --- | --- | --- | --- | --- | --- | --- | --- | --- | --- | --- | --- |
| **PTEN-M (n=79)** | **Cytoband** | **RMPA^high^ (n=166)** | | | | | | **RMPA^low^(n=168)** | | | | | |
|  |  | **SCNA(alldata)** | | **Genedosage effect** | | **Modulating effect** | | **SCNA(alldata)** | | **Genedosage effect** | | **Modulating effect** | |
|  |  | **del** | **amp** | **R** | **P** | **β** | **P** | **del** | **amp** | **R** | **P** | **β** | **P** |
| FAM45B | 10q26.11 | 150 | 0 | 0.452 | p<0.0001 | 0.399 | p<0.0001 | 23 | 1 | 0.339 | p<0.0001 | 0.237 | p<0.0001 |
| RAB11FIP2 | 10q26.11 | 150 | 0 | 0.463 | p<0.0001 | 0.623 | p<0.0001 | 24 | 0 | 0.417 | p<0.0001 | 0.566 | p<0.0001 |
| FAM45A | 10q26.11 | 150 | 0 | 0.55 | p<0.0001 | 0.416 | p<0.0001 | 23 | 1 | 0.288 | p<0.0001 | 0.25 | 0.004 |
| ATE1 | 10q26.13 | 150 | 0 | 0.419 | p<0.0001 | 0.36 | p<0.0001 | 24 | 1 | 0.344 | p<0.0001 | 0.219 | p<0.0001 |
| C10orf118 | 10q25.3 | 149 | 1 | 0.37 | p<0.0001 | 0.371 | p<0.0001 | 22 | 0 | 0.265 | 0.001 | 0.38 | p<0.0001 |
| ACADSB | 10q26.13 | 149 | 0 | 0.494 | p<0.0001 | 0.479 | p<0.0001 | 25 | 1 | 0.492 | p<0.0001 | 0.484 | p<0.0001 |
| BTRC | 10q24.32 | 148 | 0 | 0.225 | 0.003 | 0.635 | p<0.0001 | 18 | 0 | 0.348 | p<0.0001 | 0.715 | p<0.0001 |
| SLK | 10q24.33 | 148 | 0 | 0.436 | p<0.0001 | 0.294 | p<0.0001 | 19 | 0 | 0.267 | p<0.0001 | 0.523 | p<0.0001 |
| SHOC2 | 10q25.2 | 148 | 1 | 0.641 | p<0.0001 | 0.531 | p<0.0001 | 21 | 0 | 0.235 | 0.002 | 0.631 | p<0.0001 |
| FAM160B1 | 10q25.3 | 148 | 1 | 0.444 | p<0.0001 | 0.549 | p<0.0001 | 22 | 0 | 0.3 | p<0.0001 | 0.554 | p<0.0001 |
| FBXL15 | 10q24.32 | 147 | 0 | 0.276 | p<0.0001 | 0.158 | 0.006 | 7 | 5 | No effect |  |  |  |
| PPP3CB | 10q22.2 | 147 | 0 | 0.496 | p<0.0001 | 0.495 | p<0.0001 | 11 | 1 | 0.208 | 0.007 | 0.436 | p<0.0001 |
| PTEN | 10q23.31 | 147 | 0 | 0.519 | p<0.0001 | 0.203 | p<0.0001 | 16 | 0 | 0.268 | p<0.0001 | 0.562 | p<0.0001 |
| ATAD1 | 10q23.31 | 147 | 0 | 0.547 | p<0.0001 | 0.272 | p<0.0001 | 17 | 0 | 0.465 | p<0.0001 | 0.429 | p<0.0001 |
| SLC25A16 | 10q21.3 | 146 | 0 | 0.284 | p<0.0001 |  |  | 11 | 0 | No effect |  |  |  |
| HERC4 | 10q21.3 | 146 | 0 | 0.591 | p<0.0001 | 0.400 | p<0.0001 | 0 | 4 | No effect |  |  |  |
| KIAA1279 | 10q22.1 | 146 | 0 | 0.4 | p<0.0001 | 0.689 | p<0.0001 | 6 | 7 | No effect |  |  |  |
| RPP30 | 10q23.31 | 146 | 0 | 0.261 | 0.001 |  |  | 10 | 2 | No effect |  |  |  |
| DNAJC12 | 10q21.3 | 146 | 0 | 0.358 | p<0.0001 | 0.283 | p<0.0001 | 11 | 1 | 0.189 | 0.014 | 0.245 | p<0.0001 |
| NRG3 | 10q23.1 | 146 | 0 | 0.323 | p<0.0001 | 0.157 | p<0.0001 | 14 | 0 | 0.277 | p<0.0001 | 0.195 | p<0.0001 |
| GHITM | 10q23.1 | 146 | 0 | 0.59 | p<0.0001 | 0.386 | p<0.0001 | 16 | 0 | 0.245 | 0.001 | 0.611 | p<0.0001 |
| INPP5A | 10q26.3 | 146 | 1 | 0.189 | 0.015 | 0.402 | p<0.0001 | 27 | 1 | 0.233 | 0.002 | 0.517 | p<0.0001 |
|  |  |  |  |  |  |  |  |  |  |  |  |  |  |
|  |  |  |  |  |  | Page 2 |  |  |  |  |  | Table S1 | PTEN-M |
| PPP2R2D | 10q26.3 | 146 | 1 | 0.307 | p<0.0001 | 0.281 | 0.002 | 26 | 0 | 0.326 | p<0.0001 | 0.714 | p<0.0001 |
| VPS26A | 10q22.1 | 146 | 0 | 0.526 | p<0.0001 |  |  | 11 | 1 | 0.25 | 0.001 |  |  |
| ANXA11 | 10q22.3 | 145 | 0 | 0.659 | p<0.0001 | 0.228 | p<0.0001 | 15 | 1 | No effect |  |  |  |
| MORN4 | 10q24.2 | 145 | 0 | 0.225 | 0.004 | 0.337 | p<0.0001 | 18 | 0 | 0.29 | p<0.0001 | 0.492 | p<0.0001 |
| MAPK8 | 10q11.22 | 144 | 0 | 0.275 | p<0.0001 | 0.407 | p<0.0001 | 7 | 1 | No effect |  |  |  |
| CCNY | 10p11.21 | 144 | 2 | 0.781 | p<0.0001 | 0.653 | p<0.0001 | 5 | 16 | 0.376 | p<0.0001 | 0.546 | p<0.0001 |
| ZNF25 | 10p11.1 | 143 | 3 | 0.379 | p<0.0001 | 0.548 | p<0.0001 | 17 | 0 | No effect |  |  |  |
| KIAA1462 | 10p11.23 | 143 | 2 | 0.197 | 0.011 | 0.209 | p<0.0001 | 5 | 23 | 0.175 | 0.023 | 0.225 | p<0.0001 |
| WAC | 10p12.1 | 143 | 2 | 0.526 | p<0.0001 | 0.724 | p<0.0001 | 5 | 22 | 0.441 | p<0.0001 | 0.24 | 0.012 |
| NMT2 | 10p13 | 141 | 2 | 0.475 | p<0.0001 | 0.437 | p<0.0001 | 4 | 24 | 0.283 | p<0.0001 | 0.314 | p<0.0001 |
| SH3GL2 | 9p22.2 | 80 | 9 | 0.306 | p<0.0001 | 0.153 | p<0.0001 | 42 | 2 | 0.232 | 0.002 | 0.284 | p<0.0001 |
| TEF | 22q13.2 | 62 | 4 | 0.36 | p<0.0001 | 0.313 | p<0.0001 | 11 | 1 | 0.341 | p<0.0001 | 0.378 | p<0.0001 |
| ADRBK2 | 22q12.1 | 54 | 6 | 0.238 | 0.002 | 0.421 | p<0.0001 | 7 | 0 | No effect |  |  |  |
| MYCBP2 | 13q22.3 | 54 | 2 | 0.281 | p<0.0001 | 0.386 | p<0.0001 | 28 | 0 | 0.323 | p<0.0001 | 0.42 | p<0.0001 |
| RUNDC3B | 7q21.12 | 53 | 6 | No effect |  |  |  | 7 | 1 | 0.283 | p<0.0001 | 0.301 | p<0.0001 |
| PPP2R5E | 14q23.2 | 51 | 4 | 0.594 | p<0.0001 |  |  | 27 | 0 | 0.428 | p<0.0001 | 0.176 | 0.016 |
| PPM1A | 14q23.1 | 48 | 4 | 0.536 | p<0.0001 | 0.359 | p<0.0001 | 24 | 0 | 0.164 | 0.034 | 0.619 | p<0.0001 |
| TMOD2 | 15q21.2 | 38 | 4 | 0.242 | 0.002 | 0.267 | p<0.0001 | 12 | 1 | 0.306 | p<0.0001 | 0.471 | p<0.0001 |
| SH3BGRL2 | 6q14.1 | 37 | 4 | 0.218 | 0.005 | 0.358 | p<0.0001 | 11 | 1 | No effect |  |  |  |
| DOPEY1 | 6q14.1 | 37 | 4 | 0.515 | p<0.0001 | 0.31 | p<0.0001 | 11 | 1 | No effect |  |  |  |
| RNF207 | 1p36.31 | 37 | 13 | 0.171 | 0.028 | 0.078 | p<0.0001 | 67 | 1 | 0.21 | 0.006 | 0.075 | p<0.0001 |
| RUNX1T1 | 8q21.3 | 36 | 4 | No effect |  |  |  | 12 | 1 | No effect |  |  |  |
| SGSM1 | 22q11.23 | 28 | 3 | No effect |  |  |  | 12 | 1 | 0.277 | p<0.0001 | 0.252 | p<0.0001 |
| CBFA2T3 | 16q24.3 | 26 | 3 | 0.161 | 0.039 | 0.244 | p<0.0001 | 1 | 29 | No effect |  |  |  |
| SNAP91 | 6q14.2 | 25 | 4 | No effect |  |  |  | 14 | 2 | 0.273 | p<0.0001 | 0.308 | p<0.0001 |
| MADD | 11p11.2 | 25 | 1 | 0.171 | 0.027 | 0.343 | p<0.0001 | 8 | 6 | 0.176 | 0.023 | 0.514 | p<0.0001 |
|  |  |  |  |  |  | Page 3 |  |  |  |  |  | Table S1 | PTEN-M |
| PIAS1 | 15q23 | 24 | 4 | 0.251 | 0.001 | 0.346 | p<0.0001 | 14 | 2 | 0.26 | 0.001 | 0.121 | 0.033 |
| RAB11A | 15q22.31 | 23 | 5 | 0.448 | p<0.0001 |  | no | 14 | 2 | 0.171 | 0.027 |  |  |
| NTRK3 | 15q25.3 | 22 | 5 | 0.189 | 0.015 |  | no | 14 | 5 | 0.295 | p<0.0001 | 0.213 | p<0.0001 |
| ATP6V1G2 | 6p21.33 | 21 | 3 | No effect |  |  |  | 5 | 3 | No effect |  |  |  |
| GABRB3 | 15q12 | 20 | 2 | No effect |  |  |  | 2 | 19 | 0.345 | p<0.0001 | 0.234 | p<0.0001 |
| TBRG1 | 11q24.2 | 20 | 3 | 0.321 | p<0.0001 | 0.744 | p<0.0001 | 2 | 18 | No effect |  |  |  |
| CYFIP2 | 5q33.3 | 19 | 3 | No effect |  |  |  | 1 | 28 | No effect |  |  |  |
| RABGAP1L | 1q25.1 | 19 | 16 | No effect |  |  |  | 6 | 4 | 0.251 | 0.001 | 0.293 | p<0.0001 |
| CDH18 | 5p14.3 | 18 | 3 | No effect |  |  |  | 4 | 2 | No effect |  |  |  |
| KIAA0368 | 9q31.3 | 18 | 16 | 0.403 | p<0.0001 | 0.183 | 0.002 | 17 | 1 | No effect |  |  |  |
| DPP10 | 2q14.1 | 17 | 8 | No effect |  |  |  | 10 | 1 | 0.225 | 0.003 | 0.12 | p<0.0001 |
| C5orf41 | 5q35.1 | 15 | 8 | 0.175 | 0.024 | 0.29 | p<0.0001 | 11 | 2 | 0.303 | p<0.0001 | 0.182 | p<0.0001 |
| HERC1 | 15q22.31 | 14 | 0 | No effect |  |  |  | 27 | 0 | 0.362 | p<0.0001 | 0.504 | p<0.0001 |
| PCDH7 | 4p15.1 | 14 | 1 | 0.175 | 0.024 | 0.123 | p<0.0001 | 11 | 1 | No effect |  |  |  |
| UBE2D3 | 4q24 | 12 | 13 | No effect |  |  |  | 7 | 4 | No effect |  |  |  |
| PLCL2 | 3p24.3 | 12 | 14 | 0.207 | 0.007 | 0.382 | p<0.0001 | 11 | 1 | No effect |  |  |  |
| GABARAPL1 | 12p13.2 | 12 | 13 | 0.308 | p<0.0001 | 0.453 | p<0.0001 | 7 | 6 | No effect |  |  |  |
| SEC62 | 3q26.2 | 11 | 9 | No effect |  |  |  | 11 | 0 | No effect |  |  |  |
| C6orf136 | 6p21.33 | 11 | 9 | No effect |  |  |  | 18 | 2 | 0.289 | p<0.0001 | 0.422 | p<0.0001 |
| ADARB1 | 21q22.3 | 11 | 14 | 0.277 | p<0.0001 | 0.261 | p<0.0001 | 5 | 9 | No effect |  |  |  |
| FOXP1 | 3p13 | 10 | 15 | No effect |  |  |  | 9 | 2 | No effect |  |  |  |
| ARPP21 | 3p22.3 | 10 | 18 | No effect |  |  |  | 10 | 4 | No effect |  |  |  |
| HLF | 17q22 | 9 | 10 | No effect |  |  |  | 15 | 6 | No effect |  |  |  |
| CNRIP1 | 2p14 | 9 | 13 | No effect |  |  |  | 1 | 20 | 0.16 | 0.038 | 0.343 | p<0.0001 |
| C12orf51 | 12q24.13 | 8 | 6 | No effect |  |  |  | 4 | 2 | No effect |  |  |  |
| FBRSL1 | 12q24.33 | 8 | 12 | No effect |  |  |  | 1 | 4 | No effect |  |  |  |
|  |  |  |  |  |  | Page 4 |  |  |  |  |  | Table S1 | PTEN-M |
| DGKE | 17q22 | 8 | 10 | No effect |  |  |  | 1 | 4 | No effect |  |  |  |
| GRAMD1B | 11q24.1 | 7 | 6 | No effect |  |  |  | 1 | 1 | No effect |  |  |  |
| NDRG3 | 20q11.23 | 3 | 50 | 0.22 | 0.004 | 0.411 | p<0.0001 | 12 | 1 | No effect |  |  |  |
| DCUN1D5 | 11q22.3 | 2 | 22 | No effect |  |  |  | 1 | 7 | No effect |  |  |  |
| C9orf125 | 9q31.1 | 0 | 131 | No effect |  |  |  | 0 | 28 | No effect |  |  |  |

|  |  |  |  |  |  | Page 1 |  |  |  |  |  | SPRY-M | Table S1 |
| --- | --- | --- | --- | --- | --- | --- | --- | --- | --- | --- | --- | --- | --- |
| **SPRY-M (n=26)** | **Cytoband** | **RMPA^high^ (n=166)** | | | | | | **RMPA^low^(n=168)** | | | | | |
|  |  | **SCNA(alldata)** | | **Genedosage effect** | | **Modulating effect** | | **SCNA(alldata)** | | **Genedosage effect** | | **Modulating effect** | |
|  |  | **del** | **amp** | **R** | **P** | **β** | **P** | **del** | **amp** | **R** | **P** | **β** | **P** |
| DENND2A | 7q34 | 0 | 126 | 0.505 | p<0.0001 | 0.401 | p<0.0001 | 0 | 40 | 0.235 | 0.002 | 0.173 | p<0.0001 |
| ZYX | 7q34 | 0 | 125 | 0.362 | p<0.0001 | 0.534 | p<0.0001 | 0 | 41 | 0.239 | 0.002 | 0.294 | p<0.0001 |
| PDGFA | 7p22.3 | 1 | 121 | 0.349 | p<0.0001 | 0.335 | p<0.0001 | 7 | 16 | No effect |  |  |  |
| STK17A | 7p13 | 0 | 121 | 0.315 | p<0.0001 | 0.419 | p<0.0001 | 1 | 19 | 0.186 | 0.016 | 0.306 | p<0.0001 |
| JAG1 | 20p12.2 | 4 | 50 | 0.293 | p<0.0001 | 0.434 | p<0.0001 | 3 | 7 | No effect |  |  |  |
| SPRY1 | 4q28.1 | 1 | 22 | No effect |  |  |  | 0 | 7 | No effect |  |  |  |
| LYPLA1 | 8q11.23 | 2 | 22 | No effect |  |  |  | 3 | 4 | No effect |  |  |  |
| VAV3 | 1p13.3 | 3 | 19 | No effect |  |  |  | 65 | 2 | No effect |  |  |  |
| IL1RAP | 3q28 | 20 | 19 | 0.214 | 0.006 | 0.31 | p<0.0001 | 10 | 4 | No effect |  |  |  |
| LINC00152 | 2p11.2 | 12 | 18 | No effect |  |  |  | 11 | 10 | No effect |  |  |  |
| FAM100B | 17q25.1 | 11 | 15 | No effect |  |  |  | 3 | 15 | No effect |  |  |  |
| PDIA6 | 2p25.1 | 11 | 13 | No effect |  |  |  | 1 | 16 | No effect |  |  |  |
| BCAT1 | 12p12.1 | 14 | 9 | No effect |  |  |  | 16 | 1 | 0.232 | 0.002 | 0.123 | p<0.0001 |
| TRIO | 5p15.2 | 10 | 8 | No effect |  |  |  | 11 | 0 | No effect |  |  |  |
| CD151 | 11p15.5 | 16 | 8 | No effect |  |  |  | 10 | 0 | 0.218 | 0.004 | 0.183 | p<0.0001 |
| SLC27A3 | 1q21.3 | 9 | 7 | No effect |  |  |  | 6 | 3 | No effect |  |  |  |
| TRIB2 | 2p24.3 | 8 | 7 | No effect |  |  |  | 6 | 3 | No effect |  |  |  |
| TNFRSF19 | 13q12.12 | 53 | 6 | 0.431 | p<0.0001 | 0.406 | p<0.0001 | 27 | 1 | No effect |  |  |  |
| DUSP6 | 12q21.33 | 6 | 6 | No effect |  |  |  | 8 | 1 | 0.175 | 0.023 | 0.19 | p<0.0001 |
| IGFBP2 | 2q35 | 32 | 5 | No effect |  |  |  | 36 | 8 | No effect |  |  |  |
| WEE1 | 11p15.4 | 28 | 5 | 0.322 | p<0.0001 | 0.367 | p<0.0001 | 30 | 9 | No effect |  |  |  |
| RHOJ | 14q23.2 | 49 | 4 | 0.451 | p<0.0001 | 0.48 | p<0.0001 | 27 | 0 | No effect |  |  |  |
|  |  |  |  |  |  |  |  |  |  |  |  |  |  |
|  |  |  |  |  |  | Page 2 |  |  |  |  |  | SPRY-M | Table S1 |
| FABP7 | 6q22.31 | 45 | 3 | 0.226 | 0.0035 | 0.234 | p<0.0001 | 17 | 1 | No effect |  |  |  |
| SPRY2 | 13q31.1 | 54 | 3 | 0.487 | p<0.0001 | 0.47 | p<0.0001 | 30 | 1 | No effect |  |  |  |
| SPRY4 | 5q31.3 | 16 | 1 | No effect |  |  |  | 30 | 2 | No effect |  |  |  |
| CAMK2D | 4q26 | 17 | 0 | 0.248 | 0.0013 | 0.604 | p<0.0001 | 30 | 0 | 0.316 | p<0.0001 |  |  |

Gene dosage-dependent expression of the members in the NF1-M, PTEN-M and SPRY-M was analyzed using Spearman´s rank correlation, correlation coefficient (R) and their p values are indicated. Linear regression analysis between the expression of each member and the average expression of all members in the corresponding module was also performed. Regression coefficients (β values) and their p values are indicated. Data are derived from the samples in the TCGA mRNA-seq data set.

|  |  |  | Page 1 |  |  |  |  | Table S4 |  |
| --- | --- | --- | --- | --- | --- | --- | --- | --- | --- |
|  |  |  |  |  |  |  |  |  |  |
| **Table S4. Regional chromosomal alteration in RMPA^high^and RMPA^low^ gliomas from the TCGA mRNA-Seq data set.** | | | | | | |  |  |  |
|  |  |  |  |  |  |  |  |  |  |
| **RMPA^high^(n=166)** | |  |  |  |  |  |  |  |  |
| **Amplification** | |  |  |  |  |  |  |  |  |
| **cytoband** | **q value** | **residual q value** | **wide peak boundaries** | **frequency** | **genes in wide peak** | |  |  |  |
| 7p11.2 | 6.5956E-71 | 6.7203E-71 | chr7:55048002-55075819 | 85.54% | EGFR |  |  |  |  |
| 4q12 | 2.9221E-06 | 0.000015959 | chr4:54639958-54655045 | 12.65% | LNX1 |  |  |  |  |
| 12q14.1 | 0.00047933 | 0.004964 | chr12:58125396-58152580 | 24.70% | CDK4 | TSPAN31 | AGAP2 |  |  |
| 5q11.2 | 0.004964 | 0.004964 | chr5:57321587-57332330 | 39.76% | PLK2 |  |  |  |  |
| 20p13 | 0.0081577 | 0.011909 | chr20:1570787-1571324 | 40.96% | SIRPB1 |  |  |  |  |
| 1p31.1 | 0.011997 | 0.015167 | chr1:72788726-72804737 | 51.81% | NEGR1 |  |  |  |  |
| 4q34.1 | 0.011997 | 0.015167 | chr4:172375028-172377287 | 33.13% | GALNTL6 |  |  |  |  |
| 17q21.2 | 0.012026 | 0.015167 | chr17:39413411-39428141 | 37.35% | KRTAP9-9 |  |  |  |  |
| 6p21.32 | 0.015167 | 0.015167 | chr6:32501300-32530143 | 38.55% | HLA-DRB6 |  |  |  |  |
| 7q21.2 | 0.015167 | 0.015167 | chr7:92496797-92499294 | 79.52% | CDK6 |  |  |  |  |
| 9q13 | 0.015167 | 0.015167 | chr9:68183979-68186056 | 25.90% | ANKRD20A3 |  |  |  |  |
| 3q29 | 0.015167 | 0.015822 | chr3:192877903-192882902 | 34.94% | HRASLS |  |  |  |  |
| 8p23.3 | 0.015167 | 0.015822 | chr8:595444-595891 | 27.11% | ERICH1 |  |  |  |  |
| 12p13.31 | 0.016101 | 0.016101 | chr12:9673467-9690974 | 34.34% | KLRB1 |  |  |  |  |
| 4p16.1 | 0.011909 | 0.016664 | chr4:10228881-10229291 | 37.95% | WDR1 |  |  |  |  |
| 1q21.3 | 0.011909 | 0.033147 | chr1:152571170-152579467 | 39.16% | LCE3C |  |  |  |  |
| 5p11 | 0.015167 | 0.033147 | chr5:46271009-46273327 | 24.70% | HCN1 |  |  |  |  |
| 6p12.1 | 0.015167 | 0.033147 | chr6:53931085-53931418 | 24.10% | MLIP |  |  |  |  |
| 7q34 | 0.015167 | 0.033147 | chr7:142474654-142483350 | 63.86% | PRSS2 | TRY6 |  |  |  |
| 8p11.22 | 0.016188 | 0.033147 | chr8:39235604-39256060 | 40.96% | ADAM5P |  |  |  |  |
|  |  |  |  |  |  |  |  |  |  |
|  |  |  | Page 2 |  |  |  |  | Table S4 |  |
|  |  |  |  |  |  |  |  |  |  |
| **Deletion** |  |  |  |  |  |  |  |  |  |
| **cytoband** | **q value** | **residual q value** | **wide peak boundaries** | **frequency** | **genes in wide peak** | |  |  |  |
| 9p21.3 | 2.7807E-196 | 3.3235E-196 | chr9:21959052-21977193 | 71.69% | CDKN2A | C9orf53 |  |  |  |
| 1q21.3 | 3.997E-105 | 8.3628E-84 | chr1:152552820-152586539 | 45.78% | LCE3C |  |  |  |  |
| 10q11.22 | 2.0632E-77 | 2.6354E-72 | chr10:47087831-47142190 | 78.31% | LOC728643 |  |  |  |  |
| 20p13 | 2.411E-63 | 2.411E-63 | chr20:1536809-1600924 | 45.78% | SIRPB1 |  |  |  |  |
| 22q11.23 | 1.1144E-64 | 4.8982E-58 | chr22:24371071-24385545 | 43.37% | GSTT1 | LOC391322 |  |  |  |
| 1p13.3 | 6.3409E-61 | 6.3536E-49 | chr1:110226488-110256506 | 45.18% | GSTM1 |  |  |  |  |
| 11p15.4 | 1.5007E-43 | 4.2821E-34 | chr11:5776624-5841855 | 40.96% | OR52N1 | OR52N5 |  |  |  |
| 17p11.2 | 6.5488E-33 | 1.2326E-32 | chr17:18319863-18426460 | 26.51% | USP32P2 | LOC339240 | LGALS9C |  |  |
| 7q34 | 2.6635E-36 | 7.6643E-30 | chr7:142455689-142483411 | 19.28% | PRSS1 | PRSS2 | TRY6 |  |  |
| 14q24.3 | 1.033E-42 | 6.236E-29 | chr14:73992773-74017520 | 41.57% | ACOT1 |  |  |  |  |
| 11q11 | 9.7149E-28 | 1.5145E-26 | chr11:55363341-55419817 | 31.93% | OR4P4 | OR4C11 | OR4S2 |  |  |
| 8p23.1 | 2.0452E-31 | 3.9739E-25 | chr8:12162783-12260972 | 25.90% | DEFB109P1 | DEFB130 | LOC100133267 | |  |
| 3q22.1 | 7.8808E-31 | 6.2683E-25 | chr3:129695715-129819637 | 7.23% | ALG1L2 |  |  |  |  |
| 10p12.2 | 6.236E-29 | 9.5809E-25 | chr10:23730192-24837906 | 86.14% | hsa-mir-603 | KIAA1217 | MIR603 | PRINS |  |
| 8q13.3 | 8.186E-24 | 4.3151E-23 | chr8:71645688-72759772 | 7.23% | EYA1 | MSC |  |  |  |
| 5q35.3 | 7.0247E-25 | 7.976E-23 | chr5:180374524-180467481 | 23.49% | BTNL3 |  |  |  |  |
| 10p15.1 | 8.1576E-29 | 1.64E-22 | chr10:5557042-5726820 | 86.75% | CALML3 | ASB13 |  |  |  |
| 16q12.2 | 2.6392E-33 | 2.7491E-21 | chr16:55782285-55842353 | 33.13% | CES1P1 |  |  |  |  |
| 4q13.2 | 1.9384E-32 | 1.9374E-20 | chr4:69360490-69528958 | 36.14% | UGT2B17 |  |  |  |  |
| 4q13.2 | 6.0527E-28 | 4.761E-19 | chr4:70076939-70346056 | 37.95% | UGT2B28 |  |  |  |  |
|  |  |  |  |  |  |  |  |  |  |
|  | |  |  |  |  |  |  |  |  |
|  | |  |  |  |  |  |  |  |  |
|  | |  |  |  |  |  |  |  |  |
|  | |  | Page 3 |  |  |  |  | Table S4 |  |
|  | |  |  |  |  |  |  |  |  |
| **RMPA^low^ (n=168)** | |  |  |  |  |  |  |  |  |
| **Amplification** | |  |  |  |  |  |  |  |  |
| **cytoband** | **q value** | **residual q value** | **wide peak boundaries** | **frequency** | **genes in wide peak** | |  |  |  |
| 7p11.2 | 1.0394E-77 | 2.0487E-72 | chr7:54380374-54394400 | 32.14% | HPVC1 |  |  |  |  |
| 1q21.3 | 9.8856E-78 | 1.5034E-58 | chr1:152571938-152579620 | 39.29% | LCE3C |  |  |  |  |
| 5q11.2 | 4.5696E-62 | 5.3496E-58 | chr5:57329848-57332553 | 33.33% | PLK2 |  |  |  |  |
| 20p13 | 1.0193E-49 | 1.0193E-49 | chr20:1571890-1590256 | 41.07% | SIRPB1 |  |  |  |  |
| 4q34.1 | 1.0982E-51 | 5.9234E-42 | chr4:172375028-172377457 | 42.26% | GALNTL6 |  |  |  |  |
| 17q21.2 | 2.7715E-42 | 2.1371E-35 | chr17:39423211-39427849 | 41.67% | KRTAP9-9 |  |  |  |  |
| 4p16.1 | 4.696E-24 | 2.2596E-20 | chr4:10228784-10229275 | 45.83% | WDR1 |  |  |  |  |
| 3q29 | 6.6453E-25 | 7.6958E-18 | chr3:192877963-192880586 | 33.33% | HRASLS |  |  |  |  |
| 8p23.3 | 7.4776E-34 | 2.3316E-17 | chr8:594974-606116 | 27.38% | ERICH1 |  |  |  |  |
| 1p31.1 | 3.9821E-24 | 2.9238E-15 | chr1:72773430-72784153 | 42.26% | NEGR1 |  |  |  |  |
| 10p12.2 | 3.2339E-15 | 3.2309E-14 | chr10:24377676-24377919 | 29.17% | KIAA1217 |  |  |  |  |
| 6p21.32 | 4.44E-20 | 6.7208E-13 | chr6:32518770-32540476 | 35.71% | HLA-DRB6 |  |  |  |  |
| 5p11 | 1.5206E-16 | 7.7831E-13 | chr5:46271009-46278211 | 24.40% | HCN1 |  |  |  |  |
| 2q22.3 | 4.0767E-14 | 2.5227E-12 | chr2:146865328-146866706 | 36.31% | PABPC1P2 |  |  |  |  |
| 9q13 | 1.4622E-12 | 1.5085E-11 | chr9:68183979-68186058 | 19.05% | ANKRD20A3 |  |  |  |  |
| 8p11.22 | 6.6807E-16 | 5.8777E-11 | chr8:39247112-39352686 | 38.69% | ADAM3A | ADAM5P |  |  |  |
| 13q13.3 | 3.6461E-11 | 1.0812E-10 | chr13:38076301-38084731 | 35.12% | LINC00547 |  |  |  |  |
| 15q24.3 | 1.9782E-13 | 2.1716E-10 | chr15:76891512-76902193 | 30.36% | SCAPER |  |  |  |  |
| 3p14.1 | 2.0958E-10 | 5.2867E-10 | chr3:68746260-68748785 | 25.00% | FAM19A4 |  |  |  |  |
| 4p16.1 | 2.4037E-17 | 1.2364E-09 | chr4:9451924-9478848 | 19.64% | DEFB131 |  |  |  |  |
|  |  |  |  |  |  |  |  |  |  |
|  |  |  |  |  |  |  |  |  |  |
|  |  |  |  |  |  |  |  |  |  |
|  |  |  | Page 4 |  |  |  |  | Table S4 |  |
|  |  |  |  |  |  |  |  |  |  |
| **Deletion** |  |  |  |  |  |  |  |  |  |
| **cytoband** | **q value** | **residual q value** | **wide peak boundaries** | **frequency** | **genes in wide peak** | | | | |
| 1q21.3 | 8.7201E-89 | 4.1482E-61 | chr1:152552820-152586539 | 38.10% | LCE3C |  |  |  |  |
| 22q11.23 | 8.9823E-64 | 3.7937E-57 | chr22:24324874-24382761 | 22.02% | GSTTP1 | LOC391322 |  |  |  |
| 20p13 | 4.4817E-57 | 4.4817E-57 | chr20:1536809-1600924 | 32.14% | SIRPB1 |  |  |  |  |
| 11p15.4 | 7.104E-69 | 1.4594E-55 | chr11:5799831-5841855 | 27.38% | OR52N1 |  |  |  |  |
| 1p13.3 | 3.1438E-78 | 3.637E-55 | chr1:110226488-110256623 | 53.57% | GSTM1 |  |  |  |  |
| 14q24.3 | 6.292E-65 | 3.5872E-50 | chr14:73992773-74017520 | 30.95% | ACOT1 |  |  |  |  |
| 17p11.2 | 5.1156E-50 | 4.0567E-49 | chr17:18319863-18426460 | 26.19% | USP32P2 | LOC339240 | LGALS9C |  |  |
| 15q24.3 | 4.1482E-61 | 3.6061E-48 | chr15:76632488-77227699 | 8.93% | SCAPER |  |  |  |  |
| 7q34 | 4.1208E-63 | 1.3305E-45 | chr7:142455689-142483350 | 19.64% | PRSS1 | PRSS2 | TRY6 |  |  |
| 5q35.3 | 2.6661E-53 | 3.2131E-45 | chr5:180374524-180467481 | 22.62% | BTNL3 |  |  |  |  |
| 8p11.22 | 2.3009E-57 | 2.3248E-40 | chr8:39256740-39422050 | 23.21% | ADAM3A |  |  |  |  |
| 11q11 | 2.828E-40 | 2.7271E-38 | chr11:55363341-55419817 | 29.17% | OR4P4 | OR4C11 | OR4S2 |  |  |
| 19q13.41 | 6.3564E-55 | 1.841E-34 | chr19:52131814-52198653 | 61.90% | hsa-mir-125a | MIRLET7E | MIR125A | MIR99B | SIGLEC14 |
| 12p13.2 | 3.5552E-52 | 3.985E-34 | chr12:11236837-11260274 | 4.17% | TAS2R43 |  |  |  |  |
| 8p23.1 | 3.4798E-40 | 8.2191E-33 | chr8:12162783-12260972 | 22.62% | DEFB109P1 | DEFB130 | LOC100133267 | |  |
| 10p12.2 | 2.828E-40 | 4.2409E-32 | chr10:23730192-24837906 | 2.38% | hsa-mir-603 | KIAA1217 | MIR603 | PRINS |  |
| 16q12.2 | 9.163E-44 | 4.7047E-30 | chr16:55782285-55842353 | 25.00% | CES1P1 |  |  |  |  |
| 11p15.4 | 2.1111E-58 | 4.6716E-29 | chr11:4968118-5009562 | 20.83% | OR51A2 |  |  |  |  |
| 19q13.12 | 8.442E-43 | 4.6716E-29 | chr19:35840043-35896967 | 50.00% | FFAR1 | FFAR3 |  |  |  |
| 3q22.1 | 6.3334E-34 | 2.2007E-25 | chr3:129695715-129819637 | 6.55% | ALG1L2 |  |  |  |  |
|  |  |  |  |  |  |  |  |  |  |

The top 20 amplified or deleted peaks according to the residual q values are presented. The SNP6.0 data from the TCGA mRNA-seq data set were analyzed using GISTIC 2.0 at an amplitude threshold of ±0.2 under the supervision of RMPA clustering. Human genome build hg19 was used as the reference.

|  |  |  |  | Page 1 |  |  |  |  | Table S5 |  |
| --- | --- | --- | --- | --- | --- | --- | --- | --- | --- | --- |
|  |  |  |  |  |  |  |  |  |  |  |
| **Table S5.Regional chromosomal alteration in RMPA^high^ and RMPA^low^ gliomas from Rembrandts data set.** | | | | | | | | | | |
|  |  |  |  |  |  |  |  |  |  |  |
| **RMPA^high^ (n=94)** | |  |  |  |  |  |  |  |  |  |
| **Amplification** | |  |  |  |  |  |  |  |  |  |
| **cytoband** | **q value** | **residualq value** | **wide peak boundaries** | **frequency** | **genes in wide peak** | | |  |  |  |
| 7p11.2 | 8.54E-83 | 8.83E-83 | chr7:54667627-55052172 | 82.98% | EGFR |  |  |  |  |  |
| 12q14.1 | 7.87E-10 | 7.87E-10 | chr12:56263308-56745610 | 17.02% | CDK4 | CYP27B1 | B4GALNT1 | KIF5A | METTL1 | TSPAN31 |
| 12q15 | 6.63E-09 | 4.84E-07 | chr12:67295984-67955712 | 15.96% | CPM | MDM2 | RAP1B | CPSF6 | SLC35E3 | NUP107 |
| 1q32.1 | 6.53E-05 | 6.53E-05 | chr1:200569867-201502991 | 20.21% | KISS1 | MDM4 | PIK3C2B | REN | SNRPE | SOX13 |
| 4q12 | 0.007983 | 0.007983 | chr4:54652187-55499118 | 11.70% | KIT | PDGFRA | CHIC2 | GSH2 | LOC402176 | |
| 7q31.2 | 0.00893 | 0.010497 | chr7:115757904-116618770 | 80.85% | CAPZA2 | CAV1 | MET | WNT2 | ST7 | ASZ1 |
| **Deletion** | |  |  |  |  |  |  |  |  |  |
| **cytoband** | **q value** | **residual q value** | **wide peak boundaries** | **frequency** | **genes in wide peak** | | |  |  |  |
| 9p21.3 | 1.66E-77 | 2.55E-74 | chr9:21456646-22071849 | 69.15% | CDKN2A | CDKN2B | MTAP | IFNE1 |  |  |
| 1p36.23 | 1.02E-06 | 1.02E-06 | chr1:7896068-8034405 | 32.98% | TNFRSF9 | PARK7 | ERRFI1 |  |  |  |
| 10q23.31 | 0.00031 | 0.000299 | chr10:89677469-90434738 | 79.79% | LIPF | C10orf59 | LIPL1 |  |  |  |
| 13q14.2 | 0.000517 | 0.000517 | chr13:47287584-50212763 | 39.36% | RCBTB2 | 21 genes including RB1 | | |  |  |
| 9p24.3 | 9.23E-15 | 0.009065 | chr9:1-316129 | 48.94% | FOXD4 | CBWD1 | C9orf66 |  |  |  |
| 6q22.32 | 0.001733 | 0.014806 | chr6:101420223-146164445 | 24.47% | AIM1 | indlucing 185 genes | | |  |  |

|  |  |  |  | Page2 |  |  |  | Table S5 |  |
| --- | --- | --- | --- | --- | --- | --- | --- | --- | --- |
|  | |  |  |  |  |  |  |  |  |
| **RMPA^low^(n=111)** | |  |  |  |  |  |  |  |  |
| **Amplification** | |  |  |  |  |  |  |  |  |
| **cytoband** | **q value** | **Residual q value** | **wide peak boundaries** | **frequency** | **genes in wide peak** | |  |  |  |
| 12q14.1 | 6.79E-09 | 7.89E-09 | chr12:56263308-56745332 | 9.91% | 18 genes including CDK4 | |  |  |  |
| 2p24.3 | 5.7E-06 | 5.7E-06 | chr2:15759534-16359887 | 8.11% | MYCN |  |  |  |  |
| 7p11.2 | 5.7E-06 | 5.7E-06 | chr7:54667627-54912096 | 27.93% | EGFR |  |  |  |  |
| 8q24.22 | 5.7E-06 | 5.7E-06 | chr8:120721318-143040510 | 28.83% | 65 genes |  |  |  |  |
| 12q15 | 0.004757 | 0.00566 | chr12:67295984-67955712 | 7.21% | CPM | MDM2 | RAP1B | CPSF6 |  |
| 7q21.2 | 0.000608 | 0.010566 | chr7:91596494-92389778 | 41.44% | CDK6 | PEX1 | SAMD9 | GATAD1 |  |
| 12p11.22 | 0.006049 | 0.01131 | chr12:29447970-29606382 | 11.71% | TMTC1 | OVCH1 |  |  |  |
| 13q33.3 | 0.011375 | 0.011375 | chr13:108297902-109629837 | 11.71% | COL4A1 | IRS2 | MYR8 |  |  |
| 12q15 | 0.011375 | 0.012576 | chr12:69017391-69233206 | 6.31% | CNOT2 | PTPRB | KCNMB4 |  |  |
| 7q31.33 | 0.001468 | 0.027396 | chr7:90031260-158628139 | 41.44% | 452 genes |  |  |  |  |
| 4q12 | 0.027809 | 0.027809 | chr4:54296980-56021151 | 8.11% | KDR | KIT | PDGFRA | CHIC2 |  |
| 1q31.1 | 0.033463 | 0.033463 | chr1:182886426-183111389 | 14.41% | TPR | PRG4 | OCLM | C1orf27 |  |
| 12p13.32 | 0.017801 | 0.046307 | chr12:3797062-5013396 | 11.71% | 13 genes |  |  |  |  |
| **Deletion** | |  |  |  |  |  |  |  |  |
| **cytoband** | **q value** | **residual q value** | **wide peak boundaries** | **frequency** | **genes in wide peak** | | |  |  |
| 1p36.23 | 7E-18 | 7E-18 | chr1:1-13778974 | 52.25% | 168 genes | |  |  |  |
| 19q13.42 | 1.4E-10 | 1.4E-10 | chr19:60001699-61752384 | 66.67% | 55 genes | 55 genes |  |  |  |
| 9p21.3 | 2.62E-11 | 3.2E-10 | chr9:21456646-22474015 | 30.63% | CDKN2A | CDKN2B | MTAP | DMRTA1 | IFNE1 |
| 13q14.2 | 8.57E-07 | 7.49E-06 | chr13:47994024-57196411 | 31.53% | 35 genes |  |  |  |  |
| 14q24.2 | 0.000623 | 0.000645 | chr14:63956180-80110020 | 26.13% | 128 genes | |  |  |  |
| 5q35.2 | 0.001067 | 0.000996 | chr5:174888401-180857866 | 20.72% | 87 genes |  |  |  |  |
|  |  |  |  |  |  |  |  |  |  |
|  |  |  |  |  |  |  |  |  |  |
|  |  |  |  | Page 3 |  |  |  | Table S5 |  |
|  |  |  |  |  |  |  |  |  |  |
| 11p15.4 | 0.002195 | 0.002141 | chr11:8156793-12747840 | 20.72% | 31 genes |  |  |  |  |
| 9p24.3 | 0.000138 | 0.003995 | chr9:1-782986 | 23.42% | FOXD4 | ANKRD15 | CBWD1 | DOCK8 | C9orf66 |
| 10q26.13 | 0.004658 | 0.004722 | chr10:117695513-135413628 | 39.64% | 112 genes |  |  |  |  |
| 6q27 | 0.009768 | 0.009768 | chr6:102562572-170975699 | 13.51% | 209 genes |  |  |  |  |
| 21q22.3 | 0.015843 | 0.015866 | chr21:45376514-46944323 | 28.83% | 17 genes |  |  |  |  |
| 15q21.2 | 0.039923 | 0.039609 | chr15:33600671-57188190 | 31.53% | 179 genes |  |  |  |  |
|  |  |  |  |  |  |  |  |  |  |

The 50K HindIII SNP array data from the REMBRANDT data set were analyzed using GISTIC 2.0 at an amplitude threshold of ± 0.2 under the supervision of RMPA clustering. Amplified or deleted peaks with significant residual q values are presented. Human genome build hg17 was used as the reference.

|  |  |  |  | Page 1 |  |  |  | Table S6 |
| --- | --- | --- | --- | --- | --- | --- | --- | --- |
| **Table S6. RMPA^high^ and RMPA^low^ glioma-specific focal SCNAs of RTK signaling related genes in the TCGA mRNA-Seq data set.** | | | | | | | | |
|  | | |  | |  |  | |  |
| **RMPA^high^(n=166)** | | |  | |  |  | |  |
|  | **RTK signaling** | | **SCNA (focal data)** | |  | **Gene dosage effect** | |  |
|  |  |  |  |  |  |  |  |  |
|  | **Gene symbol** | **Cytoband** | **del** | **amp** | **mutation** | **R** | **P value** |  |
| **RTK Receptor** | EGFR | 7p11.2 | 2 | **88** | **45** | 0.778 | 6.07532E-35 |  |
|  | PDGFRA | 4q12 | 0 | **25** | **8** | 0.518 | 8.47512E-13 |  |
|  | KIT | 4q12 | 1 | **17** | **4** | 0.282 | 0.0002 |  |
|  | KDR | 4q12 | 1 | **13** | **3** |  |  |  |
|  | EPHB3 | 3q27.1 | 2 | **13** | **1** |  |  |  |
|  | FGFR3 | 4p16.3 | 1 | **10** | **1** |  |  |  |
|  | FGFR1 | 8p11.23 | 6 | **10** | **0** |  |  |  |
|  | MET | 7q31.2 | 3 | 7 | 1 | 0.165 | 0.03 |  |
|  | ERBB3 | 12q13.2 | **9** | 7 | 0 |  |  |  |
|  | EPHA1 | 7q34 | 2 | 6 | 3 |  |  |  |
|  | EPHB4 | 7q22.1 | 0 | 5 | 1 |  |  |  |
|  | EPHB6 | 7q34 | 2 | 5 | 1 |  |  |  |
| **RTK Ligand** | EFNB2 | 13q33.3 | 4 | **11** | 2 |  |  |  |
|  | SEMA3D | 7q21.11 | 0 | **10** | 0 |  |  |  |
|  | PDGFA | 7p22.3 | **10** | **9** | 0 |  |  |  |
|  | SEMA3A | 7q21.11 | 0 | **9** | 0 |  |  |  |
|  | FGF14 | 13q33.1 | 5 | **9** | 1 |  |  |  |
|  | FGF22 | 19p13.3 | 2 | 8 | 0 |  |  |  |
|  | FGF23 | 12p13.32 | 2 | 6 | 0 |  |  |  |
|  |  |  |  | Page 2 |  |  |  | Table S6 |
|  |  |  |  |  |  |  |  |  |
|  |  |  |  |  |  |  |  |  |
|  | FGF6 | 12p13.32 | 2 | 6 | 0 |  |  |  |
|  | NTF3 | 12p13.31 | 2 | 5 | 1 |  |  |  |
|  | EFNA1 | 1q22 | 0 | 4 | 1 |  |  |  |
|  | EFNA4 | 1q22 | 0 | 4 | 0 |  |  |  |
|  | SEMA4A | 1q22 | 1 | 4 | 1 |  |  |  |
| **MAPK**  **pathway** | BRAF | 7q34 | 3 | 4 | 3 |  |  |  |
|  | NF1 | 17q11.2 | 7 | 1 | **12** | 0.254 | 0.0009 |  |
| **AKT**  **pathway** | AKT1 | 14q32.33 | 3 | 7 | 1 |  |  |  |
|  | PTEN | 10q23.31 | **17** | 2 | **40** | 0.519 | 7.628E-13 |  |
|  | PIK3CA | 3q26.32 | 1 | **10** | **9** | 0.216 | 0.005 |  |
|  | PIK3R1 | 5q13.1 | 5 | 6 | **9** |  |  |  |
|  |  |  |  |  |  |  |  |  |

|  |  |  |  | Page 3 |  |  | Table S6 |
| --- | --- | --- | --- | --- | --- | --- | --- |
| **RMPA^low^(n=168)** | |  |  |  |  |  |  |
|  | **RTK signaling** | | **SCNA (focal data)** | |  | **Gene dosage effect** | |
|  |  |  |  |  |  |  |  |
|  | **Gene symbol** | **Cytoband** | **del** | **amp** | **mutation** | **R** | **P value** |
| **RTK Receptor** | MET | 7q31.2 | 5 | **15** | 0 |  |  |
|  | EPHA1 | 7q34 | 0 | **15** | 0 |  |  |
|  | EPHB6 | 7q34 | 0 | **15** | 3 |  |  |
|  | EPHB4 | 7q22.1 | 5 | **14** | 0 | 0.172 | 0.02 |
|  | NTRK1 | 1q23.1 | 1 | **10** | 0 |  |  |
|  | INSRR | 1q23.1 | 1 | **9** | 2 | 0.185 | 0.01 |
|  | PDGFRA | 4q12 | 1 | 7 | 1 |  |  |
|  | KIT | 4q12 | 1 | 7 | 0 |  |  |
|  | KDR | 4q12 | 1 | 7 | 0 |  |  |
|  | EGFR | 7p11.2 | 3 | 4 | 1 |  |  |
|  | EPHB3 | 3q27.1 | 3 | 4 | 0 |  |  |
| **RTK Ligand** | FGF23 | 12p13.32 | 1 | **20** | 1 | 0.282 | 0.0002 |
|  | FGF6 | 12p13.32 | 1 | **20** | 0 |  |  |
|  | NTF3 | 12p13.31 | 1 | **20** | 2 |  |  |
|  | ANGPT1 | 8q23.1 | 0 | **16** | 1 |  |  |
|  | FGF22 | 19p13.3 | 1 | **12** | 0 |  |  |
|  | SEMA4A | 1q22 | 1 | **10** | 0 |  |  |
|  | EFNB2 | 13q33.3 | 4 | 8 | 1 |  |  |
|  | EFNA1 | 1q22 | 1 | 8 | 0 |  |  |
|  | EFNA4 | 1q22 | 1 | 8 | 0 |  |  |
|  |  |  |  |  |  |  |  |
|  |  |  |  |  |  |  |  |
|  |  |  |  | Page 4 |  |  | Table S6 |
|  | SEMA3D | 7q21.11 | 5 | 6 | 0 |  |  |
|  | FGF14 | 13q33.1 | 5 | 6 | 1 |  |  |
|  | SEMA3A | 7q21.11 | 6 | 5 | 0 |  |  |
| **MAPK**  **pathway** | BRAF | 7q34 | 0 | **15** | 0 | 0.191 | 0.01 |
|  | NF1 | 17q11.2 | 1 | 0 | 0 |  |  |
| **AKT**  **pathway** | AKT1 | 14q32.33 | **10** | 0 | 0 |  |  |
|  | PTEN | 10q23.31 | **9** | 2 | 0 | 0.268 | 0.0004 |
|  | PIK3CA | 3q26.32 | 3 | 3 | 1 |  |  |
|  | PIK3R1 | 5q13.1 | 1 | 0 | 3 |  |  |
|  |  |  |  |  |  |  |  |

Focal SCNAs in the RTK signaling-related genes identified in the RMPA^high^ and RMPA^low^ gliomas from the TCGA mRNA-seq data set. Gene dosage-dependent expression of the indicated genes was identified using Spearman´s rank correlation analysis, correlation coefficients (R) and their P values are indicated.

|  |  |  |  |  |  |  | Page 1 |  |  |  |  | Table S7 |  |
| --- | --- | --- | --- | --- | --- | --- | --- | --- | --- | --- | --- | --- | --- |
| **Table S7. Odds ratio of alterations in RTK signaling pathway from RMPA^high^ gliomas** | | | | | | | | |  |  |  |  |  |
|  |  | 7p11.2 | 4q12 | 4q12 | 4q12 | 3q26.32 | 3q27.1 | 4p16.3 | 8p11.23 | 7q31.2 | 17q11.2 | 10q23.31 | 14q32.33 |
|  | **Odds ratio** | EGFR | PDGFRA | KIT | KDR | PIK3CA | EPHB3 | FGFR3 | FGFR1 | MET | NF1 | PTEN | AKT1 |
| 7p11.2 | EGFR |  |  |  |  |  |  |  |  |  |  |  |  |
| 4q12 | PDGFRA | 0.719 |  |  |  |  |  |  |  |  |  |  |  |
| 4q12 | KIT | 0.486 | 73.091 |  |  |  |  |  |  |  |  |  |  |
| 4q12 | KDR | 0.599 | 41.689 | 94.667 |  |  |  |  |  |  |  |  |  |
| 3q26.32 | PIK3CA | 0.853 | 1.694 | 1.255 | 0.970 |  |  |  |  |  |  |  |  |
| 3q27.1 | EPHB3 | 2.294 | 0.651 | 0.433 | 0.558 | 23.333 |  |  |  |  |  |  |  |
| 4p16.3 | FGFR3 | 1.151 | 0.156 | 0.228 | 0.714 | 0.241 | 0.309 |  |  |  |  |  |  |
| 8p11.23 | FGFR1 | 0.519 | 0.651 | 0.433 | 1.286 | 0.460 | 0.247 | 1.805 |  |  |  |  |  |
| 7q31.2 | MET | 1.264 | 1.861 | 1.591 | 2.074 | 0.287 | 2.238 | 1.192 | 0.933 |  |  |  |  |
| 17q11.2 | NF1 | 0.335 | 0.814 | 0.743 | 0.970 | 1.339 | 1.805 | 0.588 | 1.048 | 3.044 |  |  |  |
| 10q23.31 | PTEN | 1.107 | 0.759 | 1.382 | 1.658 | 0.402 | 1.067 | 2.201 | 0.785 | 1.010 | 0.945 |  |  |
| 14q32.33 | AKT1 | 1.264 | 0.454 | 1.591 | 0.869 | 1.691 | 4.069 | 0.459 | 0.368 | 1.450 | 0.716 | 1.010 |  |
|  |  |  |  |  |  |  |  |  |  |  |  |  |  |
|  | **p-value** | EGFR | PDGFRA | KIT | KDR | PIK3CA | EPHB3 | FGFR3 | FGFR1 | MET | NF1 | PTEN | AKT1 |
|  | EGFR |  |  |  |  |  |  |  |  |  |  |  |  |
|  | PDGFRA | 0.273 |  |  |  |  |  |  |  |  |  |  |  |
|  | KIT | 0.095 | 6.71E-14 |  |  |  |  |  |  |  |  |  |  |
|  | KDR | 0.227 | 5.00E-10 | 8.79E-13 |  |  |  |  |  |  |  |  |  |
|  | PIK3CA | 0.460 | 0.254 | 0.480 | 0.664 |  |  |  |  |  |  |  |  |
|  | EPHB3 | 0.124 | 0.444 | 0.368 | 0.492 | 3.09E-07 |  |  |  |  |  |  |  |
|  | FGFR3 | 0.528 | 0.074 | 0.160 | 0.606 | 0.176 | 0.254 |  |  |  |  |  |  |
|  |  |  |  |  |  |  |  |  |  |  |  |  |  |
|  |  |  |  |  |  |  |  |  |  |  |  |  |  |
|  |  |  |  |  |  |  | Page 2 |  |  |  |  | Table S7 |  |
|  | FGFR1 | 0.162 | 0.444 | 0.368 | 0.508 | 0.397 | 0.182 | 0.363 |  |  |  |  |  |
|  | MET | 0.488 | 0.296 | 0.418 | 0.313 | 0.232 | 0.287 | 0.604 | 0.713 |  |  |  |  |
|  | NF1 | 0.022 | 0.523 | 0.520 | 0.664 | 0.445 | 0.299 | 0.518 | 0.603 | 0.131 |  |  |  |
|  | PTEN | 0.444 | 0.342 | 0.325 | 0.233 | 0.085 | 0.553 | 0.140 | 0.447 | 0.611 | 0.560 |  |  |
|  | AKT1 | 0.488 | 0.395 | 0.418 | 0.687 | 0.392 | 0.075 | 0.396 | 0.316 | 0.541 | 0.608 | 0.611 |  |
|  | | | | |  |  |  |  |  |  |  |  |  |
| Fisher's exact test, one side p-value | | | | |  |  |  |  |  |  |  |  |  |
| OR<1 indicates tendency toward mutual exclusivity | | | | | | | |  |  |  |  |  |  |
| OR=1 indicates no association of gene alterations given the number present for each of the two genes | | | | | | | |  |  |  |  |  |  |
| OR>1 indicates tendency toward co-occurrence | | | | | | | |  |  |  |  |  |  |
|  | | | | | | | |  |  |  |  |  |  |

Mutual exclusivity and co-occurrence in RTK signaling-related SCNAs in the RMPA^high^ gliomas. Odds ratio and Fisher´s exact P values are presented.

|  |  |  | |  | |  | | |  | | |  | | | | | Page 1 | | |  | | |  | |  | |  | |  | | Table S8 | |  | |
| --- | --- | --- | --- | --- | --- | --- | --- | --- | --- | --- | --- | --- | --- | --- | --- | --- | --- | --- | --- | --- | --- | --- | --- | --- | --- | --- | --- | --- | --- | --- | --- | --- | --- | --- |
|  | **Table S8. Odds ratio of alterations in RTK signaling pathway from RMPA^low^ gliomas** | | | | | | | | | | | | | | | | | | | | | | | |  | |  | |  | |  | |  | |
|  |  | |  | |  | |  | | |  | | |  | |  | | |  | | |  | | |  | |  | |  | |  | |  | |  |
|  |  | | 7q31.2 | | 7q34 | | 7q22.1 | | | 7q34 | | | 7q34 | | 1q22 | | | 1q23.1 | | | 1q23.1 | | | 19p13.3 | | 12p13.32 | | 12p13.32 | | 12p13.31 | | 8q23.1 | |  |
|  | **Odds ratio** | | MET | | EPHA1 | | EPHB4 | | | EPHB6 | | | BRAF | | SEMA4A | | | NTRK1 | | | INSRR | | | FGF22 | | FGF23 | | FGF6 | | NTF3 | | ANGPT1 | |  |
| 7q31.2 | MET | |  | |  | |  | | |  | | |  | |  | | |  | | |  | | |  | |  | |  | |  | |  | |  |
| 7q34 | EPHA1 | | 343.000 | |  | |  | | |  | | |  | |  | | |  | | |  | | |  | |  | |  | |  | |  | |  |
| 7q22.1 | EPHB4 | | 20.000 | | 159.250 | |  | | |  | | |  | |  | | |  | | |  | | |  | |  | |  | |  | |  | |  |
| 7q34 | EPHB6 | | 84.000 | | 1333.000 | | 62.400 | | |  | | |  | |  | | |  | | |  | | |  | |  | |  | |  | |  | |  |
| 7q34 | BRAF | | 343.000 | | 9517.000 | | 159.250 | | | 1333.000 | | |  | |  | | |  | | |  | | |  | |  | |  | |  | |  | |  |
| 1q22 | SEMA4A | | 0.726 | | 2.462 | | 0.772 | | | 1.958 | | | 2.462 | |  | | |  | | |  | | |  | |  | |  | |  | |  | |  |
| 1q23.1 | NTRK1 | | 0.726 | | 2.462 | | 0.772 | | | 1.958 | | | 2.462 | | 7245.000 | | |  | | |  | | |  | |  | |  | |  | |  | |  |
| 1q23.1 | INSRR | | 1.533 | | 4.000 | | 1.635 | | | 3.133 | | | 4.000 | | 775.000 | | | 775.000 | | |  | | |  | |  | |  | |  | |  | |  |
| 19p13.3 | FGF22 | | 1.384 | | 1.986 | | 1.476 | | | 1.580 | | | 1.986 | | 0.465 | | | 0.465 | | | 0.425 | | |  | |  | |  | |  | |  | |  |
| 12p13.32 | FGF23 | | 3.536 | | 4.000 | | 3.837 | | | 3.009 | | | 4.000 | | 1.522 | | | 1.522 | | | 1.360 | | | 1.227 | |  | |  | |  | |  | |  |
| 12p13.32 | FGF6 | | 3.800 | | 4.281 | | 4.123 | | | 3.221 | | | 4.281 | | 1.614 | | | 1.614 | | | 1.442 | | | 1.301 | | 4200.000 | |  | |  | |  | |  |
| 12p13.31 | NTF3 | | 3.303 | | 3.750 | | 3.584 | | | 2.821 | | | 3.750 | | 1.439 | | | 1.439 | | | 1.286 | | | 1.160 | | 1512.000 | | 2503.000 | |  | |  | |  |
| 8q23.1 | ANGPT1 | | 2.596 | | 3.916 | | 2.790 | | | 3.011 | | | 3.916 | | 0.881 | | | 0.881 | | | 0.795 | | | 1.697 | | 8.698 | | 9.436 | | 8.059 | |  | |  |
|  |  | |  | |  | |  | | |  | | |  | |  | | |  | | |  | | |  | |  | |  | |  | |  | |  |
|  | **p-value** | | MET | | EPHA1 | | EPHB4 | | | EPHB6 | | | BRAF | | SEMA4A | | | NTRK1 | | | INSRR | | | FGF22 | | FGF23 | | FGF6 | | NTF3 | | ANGPT1 | |  |
|  | MET | |  | |  | |  | | |  | | |  | |  | | |  | | |  | | |  | |  | |  | |  | |  | |  |
|  | EPHA1 | | 5.97E-15 | |  | |  | | |  | | |  | |  | | |  | | |  | | |  | |  | |  | |  | |  | |  |
|  | EPHB4 | | 3.67E-24 | | 3.13E-13 | |  | | |  | | |  | |  | | |  | | |  | | |  | |  | |  | |  | |  | |  |
|  | EPHB6 | | 1.09E-12 | | 8.48E-19 | | 2.28E-11 | | |  | | |  | |  | | |  | | |  | | |  | |  | |  | |  | |  | |  |
|  | BRAF | | 5.97E-15 | | 1.04E-21 | | 3.13E-13 | | | 8.48E-19 | | |  | |  | | |  | | |  | | |  | |  | |  | |  | |  | |  |
|  |  | |  | |  | |  | | |  | | |  | |  | | |  | | |  | | |  | |  | |  | |  | |  | |  |
|  |  | |  | |  | |  | | |  | | |  | |  | | |  | | |  | | |  | |  | |  | |  | |  | |  |
|  |  | |  | |  | |  | | |  | | |  | | Page 2 | | |  | | |  | | |  | |  | | Table S8 | |  | |  | |  |
|  | SEMA4A | | 0.614 | | 0.256 | | 0.640 | | | 0.334 | | | 0.256 | |  | | |  | | |  | | |  | |  | |  | |  | |  | |  |
|  | NTRK1 | | 0.614 | | 0.256 | | 0.640 | | | 0.334 | | | 0.256 | | 1.85E-17 | | |  | | |  | | |  | |  | |  | |  | |  | |  |
|  | INSRR | | 0.431 | | 0.078 | | 0.404 | | | 0.123 | | | 0.078 | | 1.91E-13 | | | 1.91E-13 | | |  | | |  | |  | |  | |  | |  | |  |
|  | FGF22 | | 0.475 | | 0.327 | | 0.447 | | | 0.418 | | | 0.327 | | 0.401 | | | 0.401 | | | 0.367 | | |  | |  | |  | |  | |  | |  |
|  | FGF23 | | 0.029 | | 0.030 | | 0.022 | | | 0.065 | | | 0.030 | | 0.436 | | | 0.436 | | | 0.485 | | | 0.531 | |  | |  | |  | |  | |  |
|  | FGF6 | | 0.023 | | 0.025 | | 0.017 | | | 0.054 | | | 0.025 | | 0.411 | | | 0.411 | | | 0.458 | | | 0.503 | | 7.69E-26 | |  | |  | |  | |  |
|  | NTF3 | | 0.036 | | 0.036 | | 0.028 | | | 0.077 | | | 0.036 | | 0.461 | | | 0.461 | | | 0.510 | | | 0.557 | | 1.92E-23 | | 8.84E-25 | |  | |  | |  |
|  | ANGPT1 | | 0.124 | | 0.049 | | 0.106 | | | 0.090 | | | 0.049 | | 6.92E-01 | | | 0.692 | | | 0.652 | | | 0.388 | | 2.78E-04 | | 1.87E-04 | | 4.04E-04 | |  | |  |
|  |  | |  | |  | |  | | |  | | |  | |  | | |  | | |  | | |  | |  | |  | |  | |  | |  |
| Fisher's exact test, one side p-value | | | | | | | |  | | |  | | |  | |  | | |  | | |  | | |  | |  | |  | |  | |  | |
| OR<1 indicates tendency toward mutual exclusivity | | | | | | | | | | |  | | |  | |  | | |  | | |  | | |  | |  | |  | |  | |  | |
| OR=1 indicates no association of gene alterations given the number present for each of the two genes | | | | | | | | | | | | | | | | | | | | | | | | | | |  | |  | |  | |  | |
| OR>1 indicates tendency toward co-occurrence | | | | | | | | | | |  | | |  | |  | | |  | | |  | | |  | |  | |  | |  | |  | |
|  | | | | | | | | | | |  | | |  | |  | | |  | | |  | | |  | |  | |  | |  | |  | |

Mutual exclusivity and co-occurrence in RTK signaling-related SCNAs in the RMPA^low^ gliomas. Odds ratio and Fisher´s exact P values are presented.

**Table S9. Differentially expressed RTKs and ligands between RMPA^high^ and RMPA^low^ gliomas in at least four glioma datasets.**

|  | **Genes enriched in RMPA^high^ gliomas** | | | **Genes enriched in RMPA^low^ gliomas** | | |
| --- | --- | --- | --- | --- | --- | --- |
|  | Gene symbol | Fold change  RMPA^high^ vs. RMPA^low^ | | Gene symbol | Fold change  RMPA^low^ vs. RMPA^high^ | |
|  |  | TCGA | CGGA |  | TCGA | CGGA |
| **1** | ANGPT1 | 3.09 | 2.75 | EFNA3 | 1.82 | 2.09 |
| **2** | ANGPT2 | 3.70 | 5.41 | EFNB3 | 1.26 | 1.19 |
| **3** | AREG | 10.35 | 16.30 | EPHA10 | 4.95 | 3.81 |
| **4** | DDR2 |  | 2.14 | EPHB1 | 2.79 | 2.38 |
| **5** | EFEMP1 | 2.85 | 3.99 | EPHB6 | 3.54 | 4.35 |
| **6** | EFNA4 | 2.60 | 2.28 | ERBB3 | 2.03 | 1.45 |
| **7** | EFNB1 | 1.85 | 2.23 | ERBB4 | 3.28 | 3.12 |
| **8** | EFNB2 | 2.64 | 2.95 | FGF12 | 2.79 | 3.92 |
| **9** | EGF | 1.73 | 2.10 | FGF13 | 4.57 | 5.66 |
| **10** | EPHA2 | 3.03 | 4.06 | FGF14 | 1.28 | 2.46 |
| **11** | EPHA3 |  | 3.14 | FGF9 | 2.88 |  |
| **12** | EPHB2 | 1.64 | 1.12 | IGF1 | 1.22 |  |
| **13** | EPHB4 | 2.84 | 2.78 | L1CAM | 5.09 | 3.82 |
| **14** | ERBB2 | 1.99 | 2.10 | LMTK2 | 2.44 |  |
| **15** | FGFR1 | 1.65 | 1.88 | LMTK3 | 1.92 | 1.93 |
| **16** | FGFRL1 | 2.96 | 2.92 | NRG3 | 5.04 | 4.60 |
| **17** | FLT3LG | 1.93 | 1.54 | NTRK2 | 3.26 | 3.03 |
| **18** | HGF | 2.34 | 3.70 | NTRK3 | 1.23 | 1.77 |
| **19** | MET | 3.90 | 5.27 | PLXNB1 | 1.51 | 1.46 |
| **20** | NRP1 | 2.52 | 3.27 | PLXNB3 | 1.58 | 1.71 |
| **21** | NRP2 |  | 2.00 | SEMA3D | 5.04 | 2.94 |
| **22** | PDGFA | 4.12 | 3.05 | SEMA3G | 2.83 | 1.81 |
| **23** | PDGFD | 4.76 | 4.23 | SEMA4A | 2.54 | 2.76 |
| **24** | PDGFRB | 1.75 | 2.84 | SEMA4D | 2.05 | 1.74 |
| **25** | PDGFRL | 4.53 | 2.75 | SEMA4G | 2.45 | 1.99 |
| **26** | PLXNA3 | 1.30 | 1.64 | TNK2 | 3.21 | 3.30 |
| **27** | PLXNB2 | 1.33 | 1.59 | TYRO3 | 1.44 | 1.44 |
| **28** | PTK7 | 1.83 | 1.92 |  |  |  |
| **29** | ROR1 | 2.55 | 3.57 |  |  |  |
| **30** | RYK | 1.32 | 1.34 |  |  |  |
| **31** | SEMA3A | 3.13 | 3.00 |  |  |  |
| **32** | SEMA3F | 4.43 | 4.54 |  |  |  |
| **33** | VEGFA | 8.99 | 8.87 |  |  |  |

Differentially expressed RTKs and their ligands with a concordant expression pattern in at least four of the five glioma data sets at a p value of p = 10^-6^ and q values ranging between 2.0 x 10^-6^ and 3.9 x 10^-7^ are presented. The fold changes of their average levels between the RMPA^high^ and RMPA^low^ subtypes in the TCGA mRNA-Seq and CGGA mRNA-Seq data sets are shown.

| **Table S10. Percentages of CD45^+^,CD105^+^and CD45^-^CD105^-^ cell populations in RMPA^high^ and RMPA^low^ gliomas.** | | | | | | | | | | | |  |  |  |  |  |  |
| --- | --- | --- | --- | --- | --- | --- | --- | --- | --- | --- | --- | --- | --- | --- | --- | --- | --- |
|  |  |  |  |  |  |  |  |  |  |  |  |  |  |  |  |  |  |
|  | **RMPA^high^** | **CD45^+^** | **CD105^+^** | **CD45^-^CD105^-^** | **CD45^+^CD105^+^** |  | **RMPA^low^** | **CD45^+^** | **CD105^+^** | **CD45^-^CD105^-^** | **CD45^+^CD105^+^** |  |  |  |  |  |  |
| 1 | N11 | **26.52%** | 8.04% | **72.70%** | 7.32% | 1 | N26 | 2.29% | 2.10% | **95.7%** | 0.14% |  |  |  |  |  |  |
| 2 | N5 | 9.07% | **39.97%** | **55.50%** | 4.57% | 2 | N22 | 6.03% | 3.12% | **91.20%** | 0.34% |  |  |  |  |  |  |
| 3 | N33 | **31.11%** | **34.60%** | **55.60%** | **21.10%** | 3 | N2 | **15.13%** | **11.71%** | **82.30%** | 3.59% |  |  |  |  |  |  |
| 4 | N23 | **29.30%** | **64.1%** | **18.60%** | **12.00%** | 4 | N19 | 1.47% | 0.95% | **97.80%** | 0.18% |  |  |  |  |  |  |
| 5 | N12 | **18%** | **24.20%** | **68.80%** | **11.10%** | 5 | N8 | **18.83%** | 1.71% | **80.10%** | 0.73% |  |  |  |  |  |  |
| 6 | N9 | **17.39%** | 8.90% | **78.20%** | 4.49% | 6 | N31 | 2.43% | 2.43% | **95.60%** | 0.51% |  |  |  |  |  |  |
|  |  |  |  |  |  | 7 | N1 | 4.84% | 2.27% | **94.1%** | 1.71% |  |  |  |  |  |  |
|  |  |  |  |  |  | 8 | N15 | 3.26% | 1.35% | **95.60%** | 0.22% |  |  |  |  |  |  |
|  |  |  |  |  |  | 9 | N18 | 0.94% | 1.98% | **97.10%** | 0.04% |  |  |  |  |  |  |
|  |  |  |  |  |  | 10 | N3 | 4.08% | **33.93%** | **64.40%** | 2.33% |  |  |  |  |  |  |
|  |  |  |  |  |  | 11 | N4 | 8.78% | 4.56% | **87.8%** | 1.13% |  |  |  |  |  |  |
|  |  |  |  |  |  | 12 | N14 | 6.72% | **12.24%** | **83.60%** | 2.60% |  |  |  |  |  |  |
|  |  |  |  |  |  | 13 | N30 | 2.45% | 0.739% | **97.30%** | 0.49% |  |  |  |  |  |  |
|  |  |  |  |  |  | 14 | N21 | **21.80%** | **20.8%** | **67.40%** | **10.10%** |  |  |  |  |  |  |
|  |  |  |  |  |  | 15 | N32 | 1.04% | 0.53% | **98.50%** | 0.06% |  |  |  |  |  |  |
|  |  |  |  |  |  | 16 | N7 | **68.40%** | **20.87%** | **29.70%** | **19.00%** |  |  |  |  |  |  |
|  | | | | | | | | | | | |  |  |  |  |  |  |
| Single cells from RMPA^high^ or RMPA^low^ gliomas were co-stained with APC-conjugated anti-CD45 or anti-CD105 mAbs in combination with one of the PE-conjugated anti-RTK mAbs. The percentages of cells with indicated immunophenotypes in living cells negatively stained with 7-AAD are presented. Data are representative of at least two independent flow cytometry analyses performed for each sample. N9 and N33 originated from the RMPA^high^ samples in the CGGA mRNA-Seq data (Figure1), the other samples were the same as those described in Figure5. | | | | | | | | | | | |  |  |  |  |  |  |

| **Table S11. Cell population-based analysis of RTK expressing cells as a fraction of all living cells in glioma.** | | | | | | | | | | | | | | | | | |  |  |
| --- | --- | --- | --- | --- | --- | --- | --- | --- | --- | --- | --- | --- | --- | --- | --- | --- | --- | --- | --- |
|  |  | |  | |  | |  | | |  | | **Page1** | |  | |  | |  | **Table S11** |
|  | |  | |  | | **EGFR** | | **MET** | **VEGFR1** | | **KDR** | | **EPHB4** | | **NRP1** | | **PLXNB2** |  |  |
| **RMPA^high^(n=6)** | | **N11** | | **CD45^+^** | | 19.50 | | 19.90 | 21.80 | | 20.00 | | 21.00 | | 9.85 | | 32.20 |  |  |
|  |  |  | | **CD105^+^** | | 1.78 | | 19.80 | 17.70 | | 15.60 | | 12.70 | | 8.69 | | 17.30 |  |  |
|  |  |  | | **CD45^-^CD105^-^** | | 43.60 | | 0.42 | 2.80 | | 0.58 | | 7.60 | | 2.00 | | 39.60 |  |  |
|  |  | **N5** | | **CD45^+^** | | 13.80 | | 0.31 | 1.86 | | 1.05 | | 3.97 | | 3.88 | | 9.48 |  |  |
|  |  |  | | **CD105^+^** | | 15.10 | | 0.23 | 5.58 | | 2.89 | | 7.26 | | 9.77 | | 11.60 |  |  |
|  |  |  | | **CD45^-^CD105^-^** | | 68.90 | | 0.69 | 2.66 | | 0.79 | | 17.20 | | 21.40 | | 41.70 |  |  |
|  |  | **N33** | | **CD45^+^** | | 2.16 | | 8.81 | 15.40 | | 15.10 | | 19.60 | | 12.20 | | 17.60 |  |  |
|  |  |  | | **CD105^+^** | | 2.57 | | 7.13 | 18.50 | | 23.00 | | 17.20 | | 28.90 | | 26.80 |  |  |
|  |  |  | | **CD45^-^CD105^-^** | | 19.30 | | 1.53 | 14.40 | | 0.82 | | 4.78 | | 4.40 | | 6.50 |  |  |
|  |  | **N23** | | **CD45^+^** | | 1.52 | | 2.11 | 17.50 | | 4.60 | | 21.30 | | 3.20 | | 27.60 |  |  |
|  |  |  | | **CD105^+^** | | null | | null | null | | null | | null | | null | | null |  |  |
|  |  |  | | **CD45^-^CD105^-^** | | 35.50 | | 0.24 | 0.10 | | 0.15 | | 22.10 | | 0.69 | | 39.20 |  |  |
|  |  | **N12** | | **CD45^+^** | | 8.61 | | 1.61 | 3.08 | | 2.20 | | 5.59 | | 4.05 | | 8.20 |  |  |
|  |  |  | | **CD105^+^** | | 5.16 | | 1.15 | 4.42 | | 3.09 | | 6.49 | | 5.95 | | 5.35 |  |  |
|  |  |  | | **CD45^-^CD105^-^** | | 58.70 | | 0.42 | 0.45 | | 0.65 | | 13.10 | | 2.85 | | 18.60 |  |  |
|  |  | **N9** | | **CD45^+^** | | null | | null | null | | null | | null | | null | | null |  |  |
|  |  |  | | **CD105^+^** | | 15.20 | | 9.46 | 13.10 | | 11.70 | | 13.60 | | 8.24 | | 14.20 |  |  |
|  |  |  | | **CD45^-^CD105^-^** | | 38.80 | | 14.70 | 0.53 | | 0.26 | | 12.70 | | 12.10 | | 22.50 |  |  |
| **RMPA^low^(n=16)** | | **N26** | | **CD45^+^** | | 0.30 | | 0.80 | 0.98 | | 0.82 | | 0.82 | | 0.02 | | 0.72 |  |  |
|  |  |  | | **CD105^+^** | | null | | null | null | | null | | null | | null | | null |  |  |
|  |  |  | | **CD45^-^CD105^-^** | | 50.50 | | 0.23 | 2.33 | | 2.40 | | 0.49 | | 0.19 | | 21.90 |  |  |
|  |  | **N22** | | **CD45^+^** | | 1.85 | | 0.09 | 0.76 | | 0.13 | | 0.73 | | 0.17 | | 4.16 |  |  |
|  |  |  | |  | |  | |  |  | |  | | **Page2** | |  | |  |  | **Table S11** |
|  |  |  | | **CD105^+^** | | 6.15 | | 0.00 | 1.44 | | 0.43 | | 0.77 | | 0.41 | | 2.23 |  |  |
|  |  |  | | **CD45^-^CD105^-^** | | 75.30 | | 0.39 | 4.55 | | 5.48 | | 3.40 | | 0.52 | | 60.30 |  |  |
|  |  | **N2** | | **CD45^+^** | | 6.54 | | 10.30 | 10.70 | | 8.23 | | 10.50 | | 6.07 | | 5.73 |  |  |
|  |  |  | | **CD105^+^** | | 0.66 | | 0.96 | 0.86 | | 2.42 | | 0.94 | | 1.28 | | 0.58 |  |  |
|  |  |  | | **CD45^-^CD105^-^** | | 7.08 | | 3.34 | 2.31 | | 3.57 | | 2.88 | | 1.81 | | 6.32 |  |  |
|  |  | **N19** | | **CD45^+^** | | 0.55 | | null | 0.48 | | 0.21 | | 0.59 | | 0.17 | | 1.34 |  |  |
|  |  |  | | **CD105^+^** | | 0.38 | | null | 0.27 | | 0.17 | | 0.52 | | 0.33 | | 0.61 |  |  |
|  |  |  | | **CD45^-^CD105^-^** | | 75.00 | | null | 0.70 | | 0.46 | | 6.09 | | 1.44 | | 60.80 |  |  |
|  |  | **N8** | | **CD45^+^** | | 1.97 | | 0.53 | 1.02 | | 2.13 | | 2.65 | | 0.94 | | 8.34 |  |  |
|  |  |  | | **CD105^+^** | | 2.00 | | 0.21 | 1.34 | | 1.13 | | 1.44 | | 0.92 | | 1.55 |  |  |
|  |  |  | | **CD45^-^CD105^-^** | | 40.90 | | 0.11 | 0.16 | | null | | 0.84 | | 0.07 | | 20.60 |  |  |
|  |  | **N31** | | **CD45^+^** | | 0.21 | | 0.00 | 0.29 | | 0.17 | | 0.16 | | 0.11 | | 0.27 |  |  |
|  |  |  | | **CD105^+^** | | 3.08 | | 0.04 | 0.80 | | 0.47 | | 1.16 | | 0.23 | | 1.60 |  |  |
|  |  |  | | **CD45^-^CD105^-^** | | 52.60 | | 0.30 | 1.23 | | 1.10 | | 1.31 | | 0.72 | | 34.60 |  |  |
|  |  | **N1** | | **CD45^+^** | | 1.18 | | 0.78 | 1.57 | | 0.50 | | 1.05 | | 1.20 | | 4.33 |  |  |
|  |  |  | | **CD105^+^** | | 1.62 | | 0.58 | 0.86 | | 0.59 | | 1.15 | | 0.98 | | 1.89 |  |  |
|  |  |  | | **CD45^-^CD105^-^** | | 9.99 | | 0.65 | 1.16 | | 0.74 | | 6.88 | | 0.79 | | 12.00 |  |  |
|  |  | **N15** | | **CD45^+^** | | 0.59 | | 0.26 | 0.33 | | 0.41 | | 0.76 | | 0.22 | | 0.80 |  |  |
|  |  |  | | **CD105^+^** | | 0.43 | | 0.21 | 0.55 | | 0.34 | | 0.22 | | 0.13 | | 0.12 |  |  |
|  |  |  | | **CD45^-^CD105^-^** | | 7.21 | | 1.92 | 3.99 | | 5.95 | | 2.46 | | 0.45 | | 5.33 |  |  |
|  |  | **N18** | | **CD45^+^** | | 0.20 | | 0.00 | 0.01 | | 0.02 | | 0.14 | | 0.00 | | 0.41 |  |  |
|  |  |  | | **CD105^+^** | | 0.35 | | 0.14 | 0.06 | | 0.04 | | 0.13 | | 0.16 | | 0.04 |  |  |
|  |  |  | | **CD45^-^CD105^-^** | | 97.40 | | 2.61 | 7.93 | | 0.16 | | 6.16 | | 0.24 | | 39.00 |  |  |
|  |  | **N3** | | **CD45^+^** | | 0.58 | | 1.03 | 2.16 | | 2.32 | | 2.15 | | 0.91 | | 2.74 |  |  |
|  |  |  | |  | |  | |  |  | |  | | **Page3** | |  | |  |  | **Table S11** |
|  |  |  | | **CD105^+^** | | 3.29 | | 0.30 | 0.43 | | 0.40 | | 0.71 | | 1.25 | | 1.38 |  |  |
|  |  |  | | **CD45^-^CD105^-^** | | 15.30 | | 1.16 | 0.58 | | 0.63 | | 3.77 | | 0.41 | | 16.30 |  |  |
|  |  | **N4** | | **CD45^+^** | | 2.92 | | 0.89 | 1.34 | | 0.55 | | 2.43 | | 0.51 | | 8.36 |  |  |
|  |  |  | | **CD105^+^** | | 9.84 | | 1.41 | 1.32 | | 0.35 | | 1.77 | | 0.90 | | 3.14 |  |  |
|  |  |  | | **CD45^-^CD105^-^** | | 77.90 | | 4.75 | 20.10 | | 1.91 | | 14.70 | | 0.52 | | 45.50 |  |  |
|  |  | **N14** | | **CD45^+^** | | null | | null | null | | null | | null | | null | | null |  |  |
|  |  |  | | **CD105^+^** | | 5.21 | | 1.99 | 2.88 | | 2.66 | | 4.08 | | 1.81 | | 3.25 |  |  |
|  |  |  | | **CD45^-^CD105^-^** | | 69.10 | | 3.05 | 4.85 | | 6.19 | | 8.72 | | 2.43 | | 31.60 |  |  |
|  |  | **N30** | | **CD45^+^** | | null | | null | null | | null | | null | | null | | null |  |  |
|  |  |  | | **CD105^+^** | | 0.15 | | 0.17 | 0.36 | | 0.28 | | 0.38 | | 0.23 | | 0.11 |  |  |
|  |  |  | | **CD45^-^CD105^-^** | | 10.10 | | 0.11 | 0.11 | | 0.11 | | 0.23 | | 0.34 | | 5.94 |  |  |
|  |  | **N21** | | **CD45^+^** | | 7.45 | | null | 6.97 | | 5.44 | | 9.07 | | 7.78 | | 25.20 |  |  |
|  |  |  | | **CD105^+^** | | 11.30 | | null | 6.98 | | 6.73 | | 10.70 | | 11.50 | | 15.10 |  |  |
|  |  |  | | **CD45^-^CD105^-^** | | 36.80 | | null | 2.43 | | 2.77 | | 2.10 | | 2.76 | | 33.30 |  |  |
|  |  | **N32** | | **CD45^+^** | | 3.48 | | null | 0.07 | | 0.08 | | 0.26 | | 0.96 | | 2.49 |  |  |
|  |  |  | | **CD105^+^** | | 8.47 | | null | 0.33 | | 0.22 | | 1.09 | | 1.67 | | 5.03 |  |  |
|  |  |  | | **CD45^-^CD105^-^** | | 86.30 | | null | 0.18 | | 0.24 | | 1.52 | | 22.10 | | 83.00 |  |  |
|  |  | **N7** | | **CD45^+^** | | null | | null | null | | null | | null | | null | | null |  |  |
|  |  |  | | **CD105^+^** | | 7.15 | | null | 5.83 | | 5.36 | | 4.25 | | 5.67 | | 6.80 |  |  |
|  |  |  | | **CD45^-^CD105^-^** | | 28.80 | | null | 0.30 | | 0.07 | | 1.68 | | 0.22 | | 9.04 |  |  |

The percentages of RTK expressing cells in respective subpopulations of the RMPA^high^ or RMPA^low^ gliomas as defined in Figure S8 are shown, RTK expression in the indicated cell populations was calculated in all living cells, angiogenesis-related RTKs are frequently expressed in both CD45+ and CD105+ cell populations in the RMPA^high^ gliomas.
